# Synthesis and Characterization of Platinum(II) Complexes with Bis(3-(trifluoromethyl)-pyrazolyl)-borate Auxiliary Ligands

**DOI:** 10.1021/acs.inorgchem.5c01716

**Published:** 2025-07-30

**Authors:** Tim Riesebeck, Thomas Strassner

**Affiliations:** Physikalische Organische Chemie, 9169 Technische Universität Dresden, Dresden 01069, Germany

## Abstract

Five novel square-planar platinum­(II) complexes with electron-deficient bis­(3-(trifluoromethyl)-pyrazolyl)-borate auxiliary ligands were synthesized in yields ranging from 31% to 76%. The complexes feature cyclometalating C^C*-ligands based on phenyl imidazopyridines with different substituents (H, 3-Me, 4-Me, 4-EtO, and 4-CF_3_O). All five complexes emit light in the green-blue region with photoluminescence quantum yields between 32% and 56% (2 wt % in PMMA).

## Graphical abstract

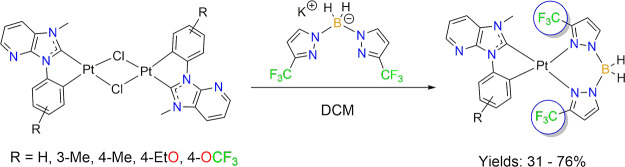

## Introduction

Platinum­(II) complexes have gained significant attention in recent years due to their remarkable photophysical properties, particularly their efficient phosphorescence,^1–9^ which makes them promising candidates for optoelectronic applications such as organic light-emitting diodes (OLEDs).^10,11^ Especially the use of cyclometalating phenylimidazole C^C*-ligands plays a crucial role in modulating their electronic structures, enhancing their emission efficiency, and tuning their emission wavelengths, as the strong σ-donating properties of the ligand effectively destabilize dark metal-centered states.^12–14^ In particular, C^C*-ligands based on imidazopyridines^15–22^ and imidazopyrazines^23–25^ demonstrated excellent photophysical properties with blue phosphorescent emission. However, complexes with such C^C*-ligands and β-diketonate auxiliary ligands show stacking effects in the solid-state leading to a significant redshift of the emission color.^15^ In addition to traditional ligand frameworks, borate-based ligands^26–29^ have emerged as an attractive class of anionic ligands since their bulkiness can effectively suppress molecular distortions in the excited state.^20^ However, the electronic effects of such borate ligands are less investigated. Although the synthesis of potassium bis­(3-(trifluoromethyl)-1*H*-pyrazol-1-yl)­borate is known,^30,31^ phosphorescent platinum­(II) complexes with such electron-poor borate auxiliary ligands have not been synthesized yet. In this study, we present the synthesis, structural characterization, and photophysical analysis of a series of phosphorescent platinum­(II) complexes featuring C^C*-ligands based on the imidazopyridine motif and trifluoromethyl substituted bispyrazolyl borate ancillary ligands.

## Results and Discussion

### Synthesis

The bis­(3-(trifluoromethyl)-1*H*-pyrazol-1-yl)­borate ligand was synthesized according to a modified published procedure.^30^ The synthesis of the imidazo-pyridine C^C*-ligand **2a** started from the *N* ^2^-phenylpyridine-2,3-diamine **1a**, followed by ring closure with formic acid, while ligands **2b**–**2e** were synthesized from commercially available 2-chloro-3-nitropyridine which was reacted with different *para*-substituted anilines yielding 3-nitro-*N*-arylpyridin-2-amines (**1b**–**1e**). The ring closure to the desired imidazo-pyridines (**2b**–**2e**) was accomplished with an excess of ammonium chloride in the presence of reductive iron-powder^19^ or with formic acid.^32^ The resulting suspensions, which were difficult to stir, were refluxed for at least 2 days. After purification by column chromatography, the desired imidazopyridines (**2a**–**2e**) were obtained in yields ranging from 40% to 92%.

The corresponding C^C*-ligand precursors (**3a**–**3e**) were obtained through quaternization reactions with methyl iodide. The yields for **3a**–**3d** are in the range of 91%–98%, while imidazolium salt **3e** was isolated in quantitative yield (Figure 1). For the synthesis of the monometallic platinum complexes, the μ-chloro-bridged bimetallic precursors (**4a**–**4e**) had to be prepared first by initial deprotonation of the imidazolium salts (**3a**–**3e**) with Ag_2_O. The addition of Pt­(COD)­Cl_2_ led to transmetalation.

**Figure 1:**
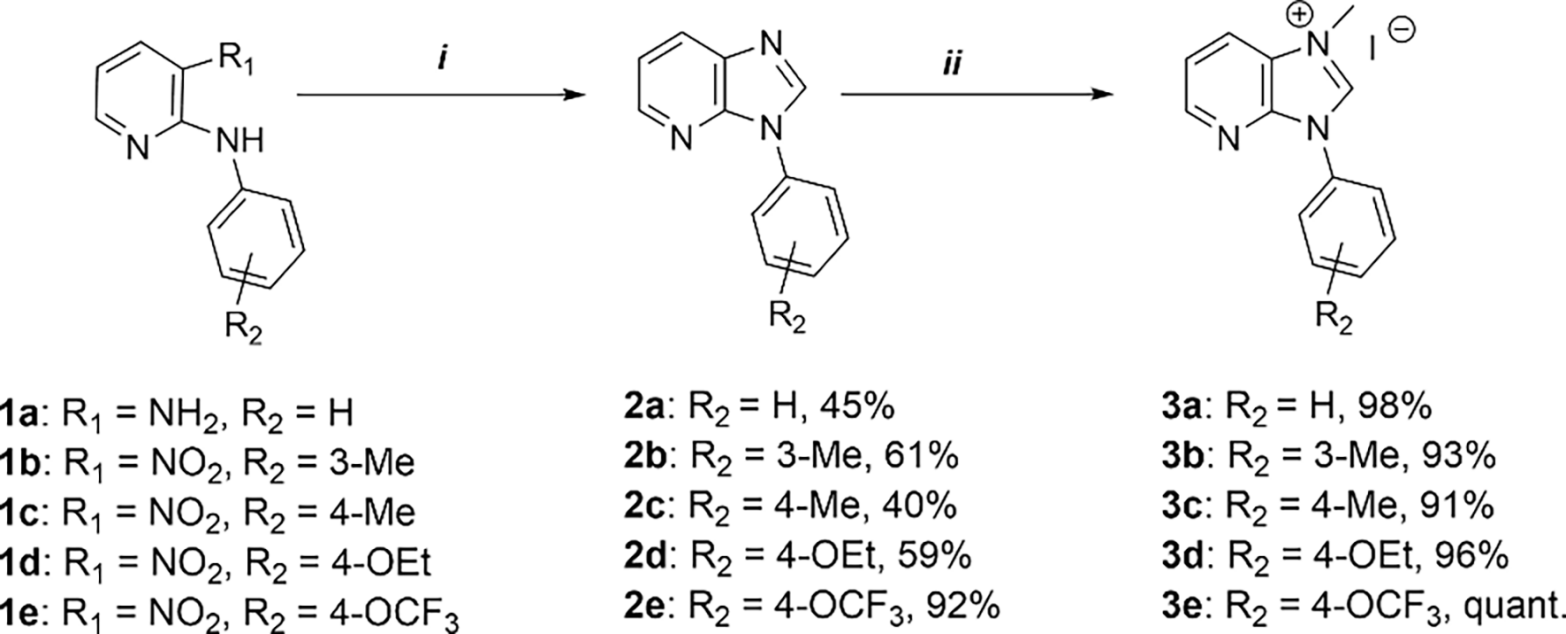
Synthesis of the ligand precursors **3a**–**3e**. (i) **2a**: HCOOH; **2b**–**2e**: NH_4_Cl, Fe, HCOOH, and isopropanol. (ii) MeI, THF, 90 °C.

Under reflux conditions in ethoxyethanol, the chloride-bridged precursors (**4a**–**4e**) formed as poorly soluble precipitates, which could be easily filtered off. Due to their poor solubility, it was not possible to sufficiently characterize these intermediates. They were used without further purification for the synthesis of the platinum­(II) complexes **5a**–**5e**. The platinum­(II) complexes were obtained by adding potassium dihydrobis­(3-(trifluoromethyl)-1*H*-pyrazol-1-yl)­borate to a suspension of the respective μ-chloro precursor in DCM and stirring the mixture at room temperature for 24 h (Figure 2). The platinum complexes were then isolated by column chromatography in yields ranging from 31% to 76%.

**Figure 2:**
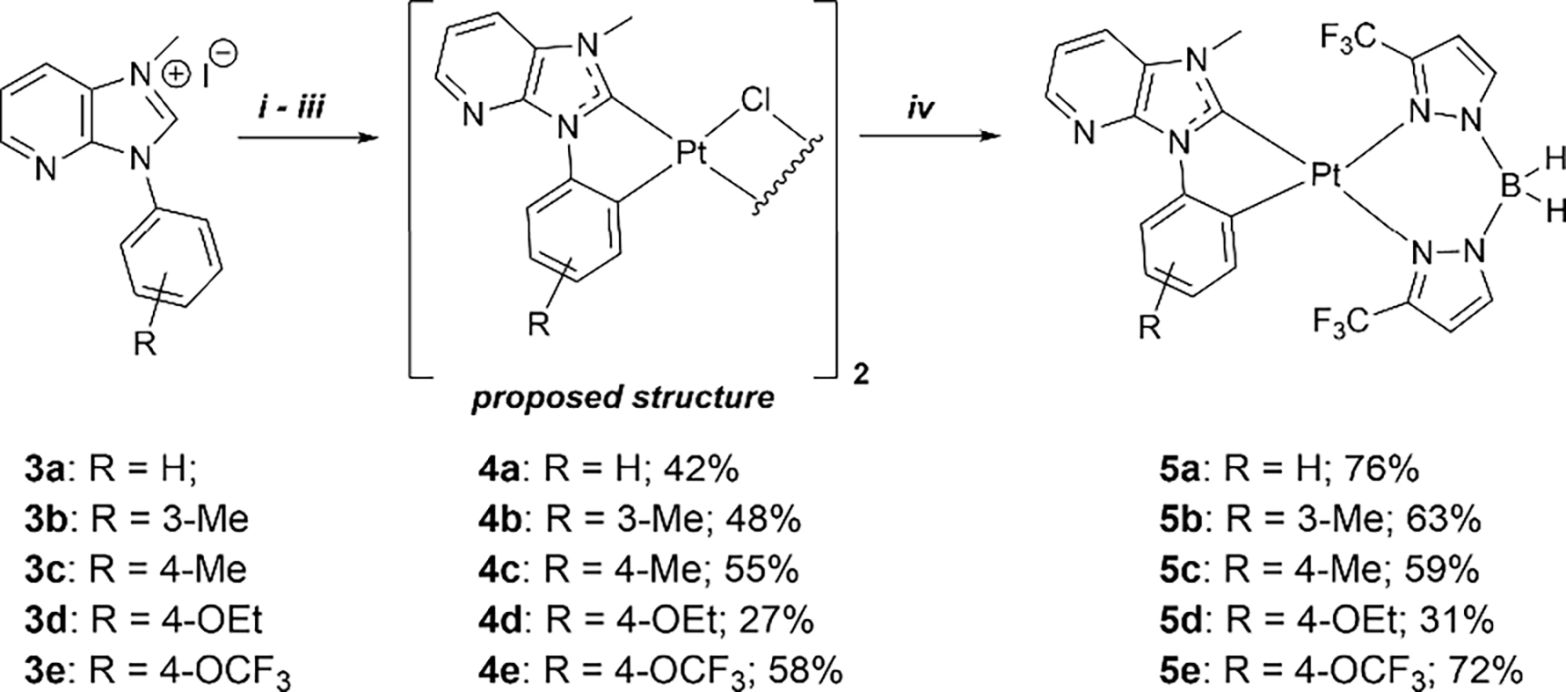
(i) Ag_2_O, DCM. (ii) Pt­(COD)­Cl_2_. (iii) Ethoxyethanol, reflux, 3 h. (iv) Potassium dihydrobis­(3-(trifluoromethyl)-1*H*-pyrazol-1-yl)­borate (**6**), DCM, 24 h.

### Structural Characterization

Complexes **5a**–**5e** were fully characterized by EA, HRMS, and NMR and additionally by two X-ray structures for complexes **5b** and **5e**. For the ligand precursors **3a**–**3c** and **3e**, the hydrogen atoms bound to the carbene carbon atom can be observed at 10.4 ppm in the ^1^H NMR spectrum. For compound **3d**, the respective signal is observed at 10.3 ppm, suggesting a stronger shielded carbene proton due to the strong electron donating properties of the *para*-ethyloxyphenyl ring bound to the NHC. During the synthesis of the respective platinum complexes, these signals can no longer be observed in the ^1^H NMR spectrum, as the free carbene is initially formed by deprotonation before it coordinates to the central metal atom.

The ^19^F NMR spectra further support the successful synthesis of complexes **5a**–**5e**, as two distinct, well-resolved signals are observed between −57 and −61 ppm. Moreover, the ^1^H NMR spectra indicate that the 3-trifluoromethylpyrazolylborate ligand remains intact during the synthesis, as two broad signals for the BH_2_-group were detected between 3 and 5 ppm. Compared to previously synthesized platinum­(II) complexes^33,34^ with bis­(pyrazolyl)-borate ligands, the hydride signals of the 3-trifluoromethylpyrazolylborate ligand are more deshielded.

Complexes **5b** and **5e** crystallize in the space groups *C*2/*c* and *P*-1. The central Pt­(II) atom is coordinated by two ligands, where the two carbon atoms of the C^C*-ligand and the two nitrogen atoms of the pyrazolyl rings of the borate ligand are bound to the platinum­(II) center.

The Pt–C and Pt–N bond lengths in the solid-state structure of complex **5b** (cf. Figure 3 and Figure S78) are 1.955(3) Å for Pt(1)–C(1), 2.006(3) Å for Pt(1)–C(9), 2.113(2) Å for Pt(1)–N(4), and 2.083(2) Å for Pt(1)–N(7), while for complex **5e** (cf. Figure 4 and Figure S79), they were found to be 1.953(3) Å for Pt(1)–C(1), 2.006(4) Å for Pt(1)–C(9), 2.101(3) Å for Pt(1)–N(7), and 2.099(2) Å for Pt(1)–N(4). These values are typical for a square-planar coordination of cyclometalated NHC-platinum­(II) complexes with bispyrazolylborates.^20^ The bond length between the carbon atom of the methyl group and the carbon atom of the cyclometalated ring (**5b**: C(12)–C(14)) was determined to be 1.512(5) Å. The bond length of the trifluoromethoxy group to the aromatic ring (C(11)–O(1)) for complex **5e** is 1.424(5) Å. The bond angles between the ligands for complex **5b** are 101.6(1)° for the C(1)–Pt(1)–N(4) angle (**5e**: C(1)–Pt(1)–N(7) 99.3(1)°), 81.24(8)° for N(4)–Pt(1)–N(7) (**5e**: N­(4)–Pt­(1)–N­(7) 82.6(1)°), 97.2(1)° for C(9)–Pt(1)–N(7) (**5e**: C(9)–Pt(1)–N(4) 98.7(1)°), and 79.9(1)° for C(1)–Pt(1)–C(9) (**5e**: C­(1)–Pt­(1)–C­(9) 79.6(1)°). The bond lengths and angles show slight deviations from the ideal angles of a square-planar geometry.

**Figure 3:**
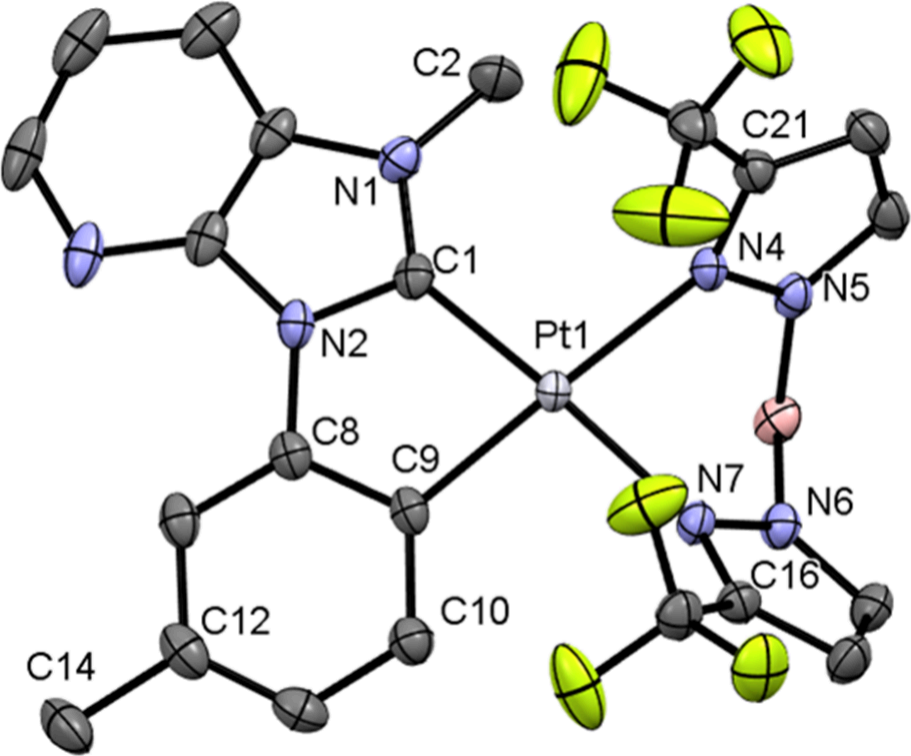
Crystal structure of complex **5b** (*C*2/*c*) with thermal ellipsoids at 50% probability. Relevant bond lengths [Å], angles, and torsion angles [°]: Pt(1)–C(1) 1.955(3), Pt(1)–C(9) 2.006(3), Pt(1)–N(4) 2.113(2), Pt(1)–N(7) 2.083(2), C(12)–C(14) 1.512(5), N(1)–C(2) 1.452(2), C(1)–Pt(1)–N(4) 101.6(1), N(4)–Pt(1)–N(7) 81.24(8), C(9)–Pt(1)–N(7) 97.2(1), C(1)–Pt(1)–C(9) 79.9(1), N(1)–C(1)–Pt(1)–N(4) 15.1(3), N(7)–Pt(1)–C(9)–C(10)−5.0(3).

**Figure 4:**
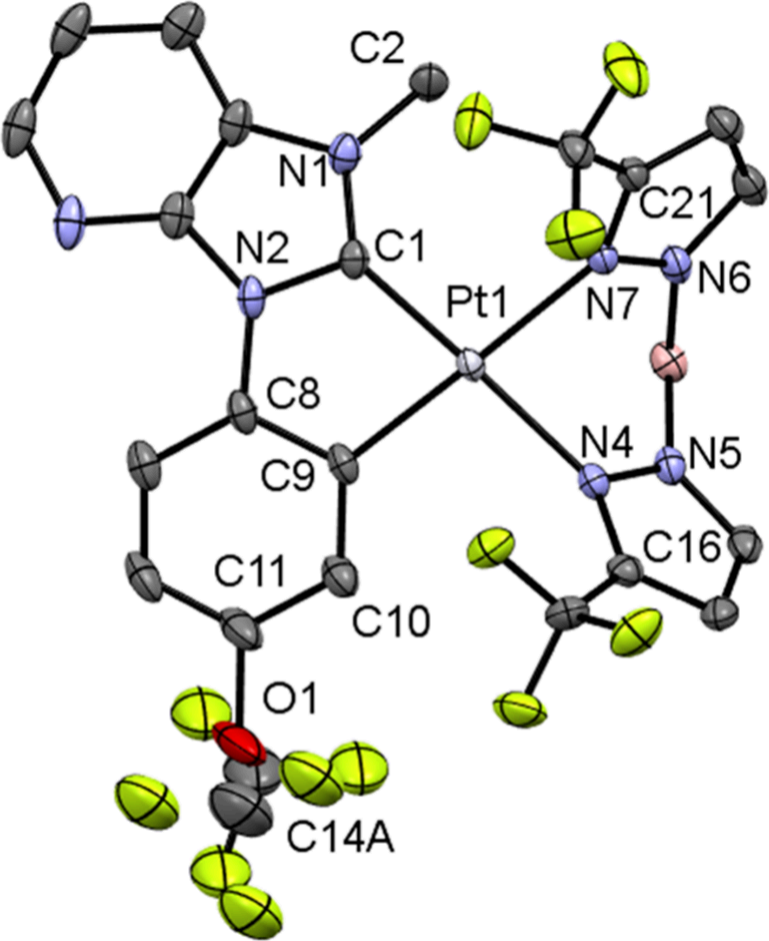
Crystal structure of complex **5e** (*P*-1) with thermal ellipsoids at 50% probability. Relevant bond lengths [Å], angles, and torsion angles [°]: Pt(1)–C(1) 1.953(3), Pt(1)–C(9) 2.006(4), Pt(1)–N(7) 2.101(3), Pt(1)–N(4) 2.099(2), C(11)–O(1) 1.424(5), N(1)–C(2) 1.349(4), C(1)–Pt(1)–N(7) 99.3(1), N(4)–Pt(1)–N(7) 82.6(1), C(9)–Pt(1)–N(4) 98.7(1), C(1)–Pt(1)–C(9) 79.6(1), C(11)–O(1)–C­(14A) 110.5(7), N(1)–C(1)–Pt(1)–N(7) 18.3(4), N(4)–Pt(1)–C(9)–C(10) −4.8(3). The disordered OCF_3_-group was modeled over two positions with site occupancies refined to 51.6 and 48.4%. Equivalent anisotropic displacement parameters (EADP) were applied to corresponding atoms in both conformations.

This distortion can be attributed to steric or electronic effects of the ligands, as the trifluoromethyl groups of the bispyrazolylborate, in particular, require more space compared to, for example, the methyl groups of similar borate ligands.^20^ The dihedral angles for complex **5b**, particularly 15.1(3)° for N(1)–C(1)–Pt(1)–N(4) (18.3(4)° for **5e**) and –5.0(3)° for N(7)–Pt(1)–C(9)–C(10)(−4.8(3) for **5e**), indicate that the ligands are slightly twisted around the coordination plane of the platinum atom, leading to a slight distortion of the molecular structure. The shortest Pt–Pt distances in the packing diagram (Figures S80 and S81) of complex **5b** are 7.722 Å, while for complex **5e**, they are found to be 8.560 Å. These distances are significantly larger than the sum of the van der Waals radii of platinum (3.5 Å), suggesting that Pt–Pt interactions can be excluded. Furthermore, the angles between the C^C*-ligand and the borate ligand were determined (Figures S83 and S84) as 50.42° for complex **5b** and 49.53° for complex **5e**. The angles are comparatively large due to the bulky CF_3_-groups which in turn reduce the through-space distance between the central Pt-atom to the closer H atom of the BH_2_-fragment of the borate ligand. For complex **5b** a distance of 2.88 Å was measured and for **5e** 2.84 Å, respectively. The through-space Pt–H-distances are smaller than the sum of the van-der-Waals radii of platinum and hydrogen (2.95 Å).

### Photophysical Characterization

The UV/vis spectra of **5a**–**5e** are shown in Figure 5. All complexes begin to absorb in the range between 350 and 375 nm, followed by an absorption band at 325 nm in the spectra of all complexes, with the highest intensity observed for complex **5a**. In the absorption spectrum of complex **5d**, however, this band can only be observed as a shoulder. Additionally, the absorption spectra of all other complexes are characterized by two additional bands at 310 and 290 nm.

**Figure 5:**
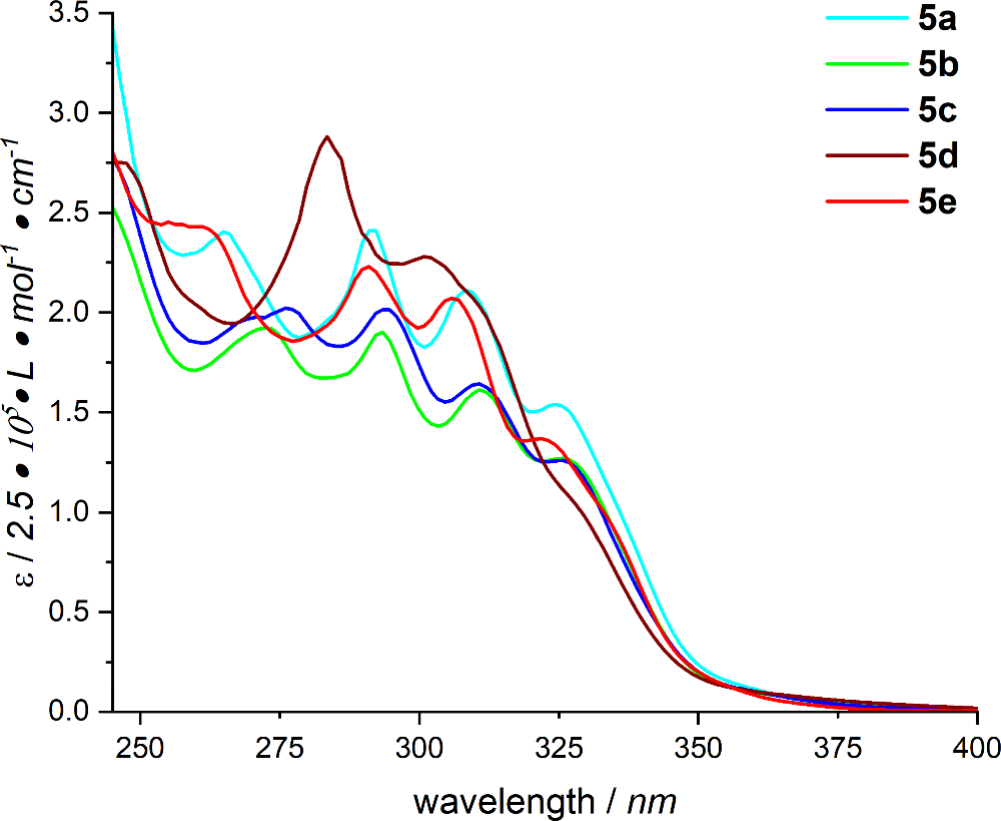
UV/vis absorption spectra of the platinum complexes **5a**–**5e** recorded in dichloromethane solutions (*c* = 5 × 10^–5^ M) in a quartz glass cuvette (*d* = 1 cm) at room temperature.

In the absorption spectra of complexes **5b** and **5c**, these bands are blue-shifted by 5 nm. Another band can be observed in the range between 250 and 275 nm for all complexes except complex **5d**. The intensity of this band is higher for complexes **5a** and **5e** compared to the two methylphenyl-substituted platinum complexes (**5b** and **5c**). The absorption spectrum of complex **5d** structurally differs from those of the other complexes. It is less defined, particularly in the range between 300 and 325 nm, and exhibits higher intensities. A particularly intense band can be observed at 275 nm. Notably, complexes with a phenyl or *para*-trifluoromethoxyphenyl substituent at the imidazopyridine ligand generally show a slight blue shift compared to more electron-rich C^C*-ligands, such as **5b** and **5c.**

The position of the methyl group on the phenyl ring of complexes **5b** and **5c** does not have a significant influence on the absorption behavior, as both complexes show nearly identical absorption spectra.

The emission properties of the new platinum­(II) complexes were investigated in a PMMA matrix using photoluminescence spectroscopy at room temperature and in 2-MeTHF at 77 K. The excited-state lifetimes were estimated using time-correlated single-photon counting (TCSPC).

To model the emission behavior of the complexes as potential emitters for OLED applications, 60 μm thick PMMA layers containing 2 wt % emitter were deposited onto a quartz glass substrate. The photoluminescence spectra of complexes **5a**–**5e** are shown in Figure 6. All complexes emit in the blue-green region of the visible light spectrum.

**Figure 6:**
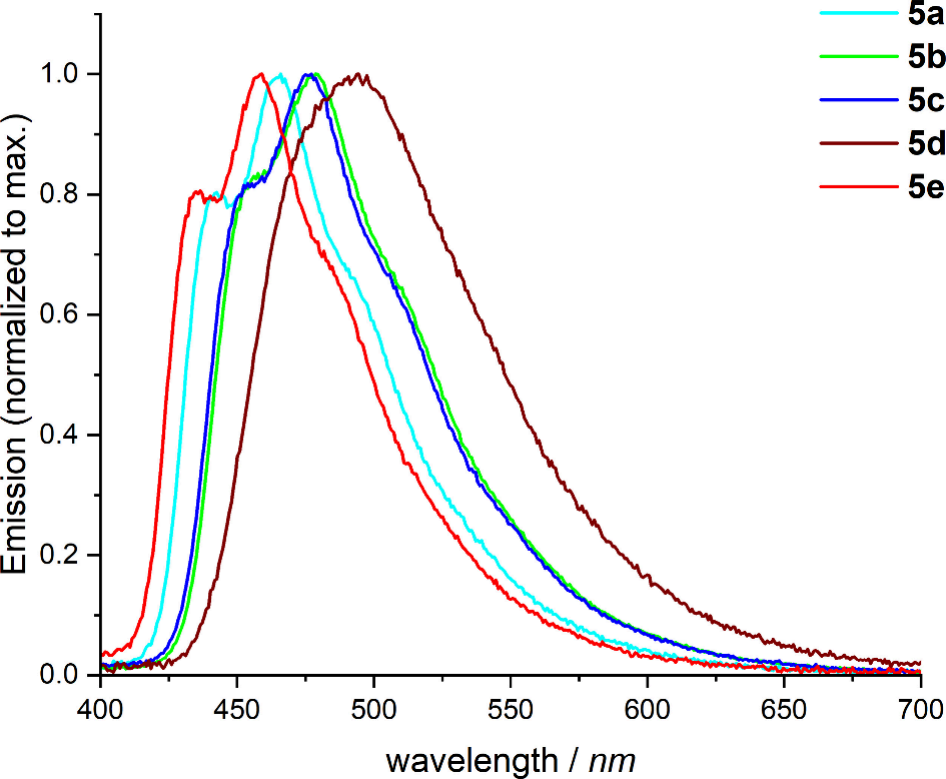
Emission spectra of complexes **5a**–**5e** in a PMMA matrix (2 wt % emitter load) measured at room temperature with an excitation wavelength of 305 nm.

The emission spectra of complexes **5b** and **5c** are identical as well as their absorption spectra (Figure 5). The emission maximum is located at 477 nm. Due to the poorly resolved vibronic fine structure of the spectra, additional bands are only recognizable as shoulders at 453 and 500 nm. Compared to the emission spectra of the methyl-substituted C^C*-platinum­(II) complexes, the emission maxima of complexes **5a** and **5e** are at 465 and 458 nm, respectively. In contrast, the emission spectrum of complex **5d** is featureless and red-shifted compared to the spectra of the other complexes, with an emission maximum at 493 nm. The emission spectra of the more electron-deficient platinum­(II) complexes are more structured than that of complex **5d**. Changes in the emission spectra with increasing emitter concentration in the PMMA-matrix were not observed.

Therefore, we propose that stacking effects in the solid-state do not play a role. In comparison to similar complexes with β-diketonate auxiliary ligands,^15,19^ the CF_3_-substituted bispyrazolyl borate ligand effectively suppresses stacking.

The quantum yield and other photophysical data of all complexes were determined (cf. Table 1). The highest quantum yield measured in this series of platinum­(II) complexes is 56% for the *meta*-methyl derivative (**5b**), followed by 51% for the *para*-methyl derivative (**5c**). Complex **5a**, featuring the unsubstituted phenylimidazo-pyridine C^C*-ligand, has a quantum yield of 41%, while for the ethoxy derivative (**5d**), a quantum yield of 37% was found. The lowest quantum yield was measured for the *para*-trifluoromethoxy derivative (**5e**) with 32%. The excited-state lifetimes are in the range of 13 μs (**5b**), 15 μs (**5a**, **5c**), and 19 μs (**5e**), while the longest lifetime was measured for **5d** with 21 μs. The relatively low quantum yields and high nonradiative decay rates suggest that, in addition to radiative decay from a charge-transfer excited state, competing nonradiative decay channels are operative and deactivate the excited state.

**Table 1:** Photoluminescence Data of **5a**–**5e** ^g^

	Φ [%]^a^	λ_em_ [nm]^b^	CIE *x*;*y* ^c^	τ_0_ [μs]^d^	*k*_r_ [10^3^·s^–1^]^e^	*k*_nr_ [10^3^·s^–1^]^f^
**5a**	41	465	0.16;0.18	15	68	98
**5b**	56	477	0.17;0.26	13	77	60
**5c**	51	477	0.17;0.25	15	65	62
**5d**	37	493	0.21;0.39	21	48	81
**5e**	32	458	0.16;0.15	19	52	111

a PLQY at room temperature and λ_exc_ = 305 nm.

b Emission wavelength with maximum intensity at room temperature.

c CIE color coordinates.

d τ_0_ = τ_exp_/Φ.

e *k*_r_ = Φ/ τ_exp_.

f *k*_nr_ = (1 – Φ)/τ_exp_.

g PL in a PMMA matrix (2 wt % emitter load, λ_exc_ = 305 nm).

To suppress thermally induced transitions and vibrations, the photoluminescence spectra of complexes **5a**–**5e** were measured at cryogenic temperatures. For this purpose, solutions of the complexes in 2-methyltetrahydrofuran (2-MeTHF) with a concentration of 5 × 10^–4^ M were prepared and measured at 77 K. The spectra are shown in Figure 7. Compared to the PL spectra recorded at room temperature (see Figure 6), the PL spectra at 77 K are blue-shifted by 10 nm. The measured quantum yields at 77 K are all 100%, which is typically observed for phosphorescent platinum complexes. All spectra measured at 77 K feature four distinguishable vibronic transitions of varying intensities. The emission maxima in the PL spectra of the complexes at 77 K are comparable to those at room temperature.

**Figure 7:**
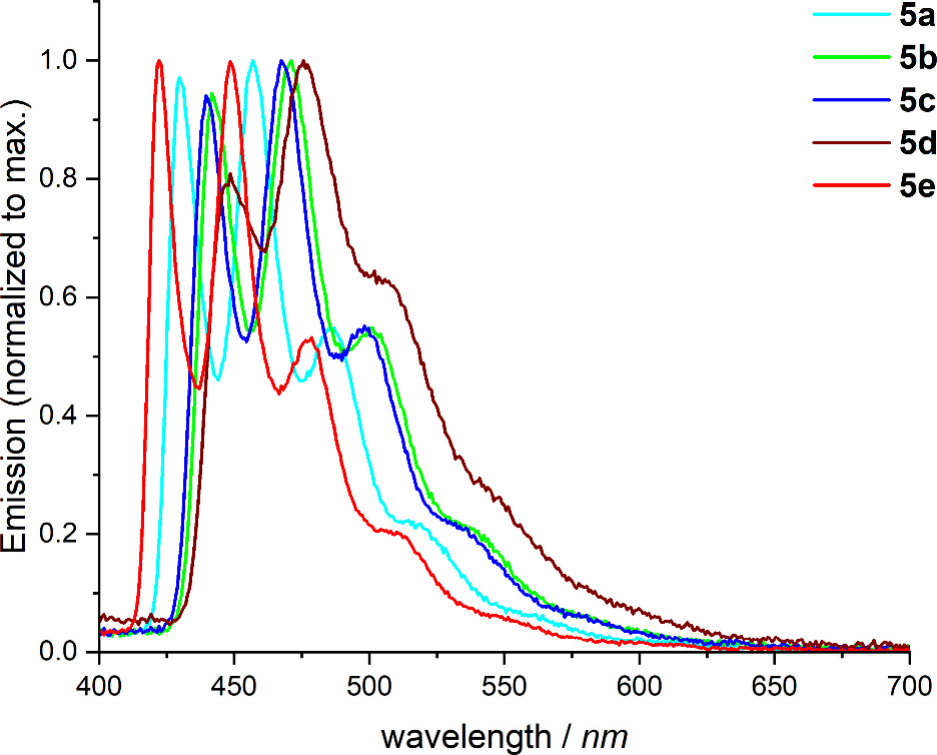
Emission spectra of complexes **5a**–**5e** measured in 2-MeTHF (*c* = 5 × 10^–4^ M) at 77 K with an excitation wavelength of 305 nm.

For example, the emission maxima of **5e** (red spectrum, Figure 6) are located at 435, 458, and 483 nm in PMMA films at room temperature, whereas in 2-MeTHF at 77 K (Figure 7), they appear at 422, 448, 478, and 510 nm, with the lowest-energy transition only visible as a small shoulder. Vibronic progressions with spacings between 1300–1400 cm^–1^ are observed in the 77 K PL spectra of all complexes. These progressions are indicative of intraligand (C^C*) ^3^π–π* transitions.^35–37^ While at room temperature the central maximum is the most intense, the two highest-energy transitions exhibit equal intensity at 77 K. Again, the spectrum of complex **5d**, recorded at 77 K, differs from the emission spectra of the other complexes. The PL spectrum of **5d** features a single global maximum at 477 nm, with the other peaks being less pronounced which suggests that other transitions contribute more intensively to the emission (cf. NTO-analysis of **5d** in Figure S88). The PL spectra, especially those recorded at 77 K, reveal strong vibronic progressions, which suggest an excited state with ^3^ILCT character. The relatively long excited state lifetimes and moderate quantum yields, however, indicate a rather weak ^3^MLCT contribution to the excited state.

### Quantum Chemical Calculations

In addition to the experimental investigations, DFT calculations were performed. For this purpose, the Gaussian16 software package was used.^38,39^ The calculations were carried out using the hybrid functional B3LYP^40–44^ in combination with the triple-ζ basis set 6-311++G** ^45–47^ and dispersion correction (D3BJ).^48,49^ Additionally, platinum was described using a LANL2TZ effective core potential (ECP).^50–54^ The geometries of the ground state and the first excited triplet state were optimized for complexes **5a**–**5e**. Based on these calculations, the HOMOs and LUMOs of the complexes were visualized and analyzed. The spin density was also evaluated for the excited state (for complex **5b** see Figure 8, other complexes see Figures S84–S87). For all five complexes in this series, the HOMOs, LUMOs, and spin density are evenly distributed, with no significant differences observed. The HOMO and LUMO are located on the C^C*-ligand and the central metal atom. The HOMO is primarily distributed over the cyclometalated phenylring and the platinum center, with a small fraction also extending onto the NHC ligand. The LUMO is mainly localized on the imidazopyridine motif and the metal atom. The HOMO exhibits dπ character, while the LUMO has predominantly dπ* character. Electron-donating substituents such as ethoxy and methyl substituents destabilize the HOMO which results in a smaller HOMO–LUMO gap and a red-shifted emission.

**Figure 8:**
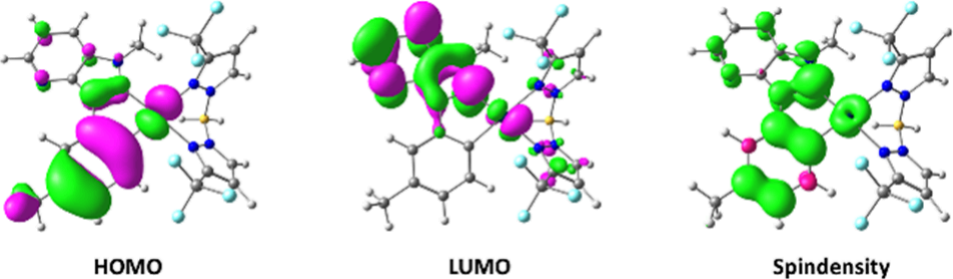
Optimized ground-state geometry with HOMO and LUMO distribution plots (isovalue = 0.03) of complex **5b**. Optimized excited-state geometry and spin density distribution plot (isovalue = 0.004) of complex **5b**.

In contrast, the −OCF_3_-group exerts a strong negative inductive effect stabilizing the HOMO which leads to blue-shifted emission spectrum of complex **5e.**

The spin density is also mainly distributed over the C^C*-ligand and the metal center, with the imidazopyridine motif being less populated than the cyclometalated phenylring. The geometries of the ground state and the excited state do not differ significantly. Therefore, the relatively low quantum yields do not necessarily result from major structural changes in the molecular geometry.

To gain a deeper understanding of the excited state of complexes **5a**–**5e**, TD-DFT calculations were performed at the B3LYP­(D3)/6-311++G** level to compute the first 50 excited singlet states. Based on these calculations, the experimentally recorded UV/vis spectrum of complex **5b** was compared with the computed spectrum (Figure 9).

**Figure 9:**
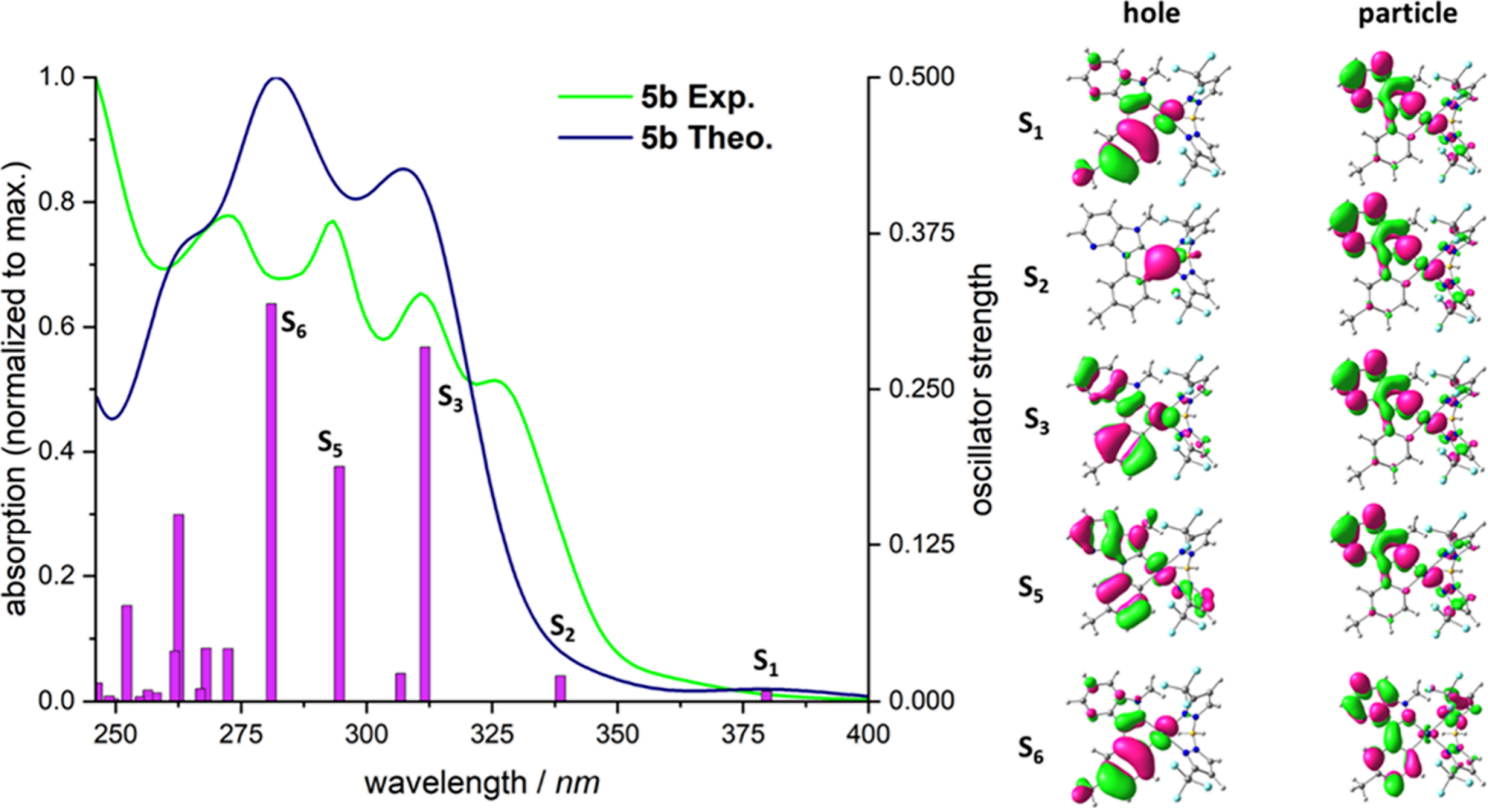
NTO analysis of complex **5b**.

The calculated and the experimentally recorded spectrum of complex **5b** are in good agreement. The experimental spectrum is characterized by three strong transitions at 290, 310, and 325 nm. The simulated transitions (Figure 9 and Figures S3, S5, and S6) with corresponding large oscillator strengths are slightly blue-shifted compared to the experimentally observed bands. Specifically, the S3 transition corresponds to the experimentally observed band at 325 nm, S5 to the band at 310 nm, and S6 to the band at 290 nm. The simulated spectrum begins at 380 nm with a weak transition corresponding to the band gap, representing the charge transfer from the HOMO to the LUMO, which is mainly localized on the imidazopyridine moiety. The S2 transition is also relatively weak (oscillator strength = 0.02) and involves a transition from a d_*z*_ orbital to the LUMO, indicating a metal-to-ligand charge transfer (^1^MLCT). The S3 transition occurs from a dπ orbital, located on the entire C^C*-ligand to the LUMO which is mainly located on the imidazopyridine motif. This transition can be ascribed as an intraligand charge transfer (^1^ILCT) with ligand-centered (^1^LC) admixture and metal contribution. A similar observation applies to the S5 transition, which also involves excitation from a dπ orbital, though originating from a different metal d orbital. Here as well, the excitation occurs into the LUMO, confirming an ^1^ILCT/LC character. The S6 transition can be described as a ππ* transition with minimal involvement of the metals d orbitals. This LC transition predominantly occurs on the C^C*-ligand. Unlike the other complexes, the experimentally recorded spectra of complex **5d** are different. The emission spectrum, measured in PMMA at room temperature is structureless with a large full width at half-maximum (fwhm). The absorption spectrum is also less structured compared to the absorption spectra of the other complexes. This suggests that the electronic transitions also differ from those of the other complexes (see Figure S88). The NTO-analysis for complex **5d** reveals that the S1 and S2 transitions occur at the same wavelength and with similar weak intensities as for the other complexes. However, the calculated S3 transition appears significantly weaker (oscillator strength = 0.08) and is also red-shifted. This transition exhibits ^1^ILCT character, where electron density is transferred from the *para*-ethoxyphenyl substituent and the metal into the LUMO. The S5 transition is relatively strong (oscillator strength = 0.44) but still occurs around 300 nm. It corresponds to a dπ to dπ* transition (^1^MLCT/LC). The S6 transition is also pronounced (oscillator strength = 0.41), has ππ* character, and is primarily located on the C^C*-ligand.

## Conclusions

Five novel platinum phosphors featuring C^C*-ligands, based on differently substituted phenyl imidazopyridines and bis­(3-(trifluoromethyl)-1*H*-pyrazol-1-yl)­borate ligands were synthesized with yields ranging between 31% and 76% via bimetallic μ-chloro precursors. The solid-state structures of **5b** and **5e** suggest that the distance between platinum center and the H atom of the BH_2_-group is only 2.88 Å and 2.84 Å, respectively. All complexes emit light in the green-blue region of the visible light spectrum with quantum yields ranging between 32% and 56%. The decay rates of the emission are between 13 and 21 μs. DFT calculations indicate that the emissive state is mainly dominated by the C^C*-ligand.

## Experimental Section

### Materials and Methods

All compounds were synthesized in oven-dried Schlenk tubes under an atmosphere of argon unless otherwise noted. Solvents of at least 99.0% purity were used in all reactions in this study. Dimethylformamide (DMF) was dried using standard techniques and stored over molecular sieve (3 Å). Dichloro­(cycloocta-1,5-diene)-platinum­(II) was prepared according to a modified literature procedure.^55^ All other chemicals were obtained from common suppliers and used without further purification. ^1^H, ^13^C, ^19^F and ^195^Pt NMR spectra were acquired on Bruker Avance 300 and Bruker Avance 600 NMR spectrometers. Chemical shifts are given in ppm relative to TMS (^1^H, ^13^C), CCl_3_F (^19^F) and Na_2_PtCl_6_ (^195^Pt). The residual solvent signals were used as reference resonances (^1^H: 5.32 ppm, ^13^C 54.0 ppm for CD_2_Cl_2_; ^1^H: 2.50 ppm, ^13^C: 39.43 ppm for DMSO-*d*_6_). Universal calibration was used for ^19^F (Ξ = 0.94094011) and ^195^Pt (Ξ = 0.21496784) measurements.^56^ Chemical shifts are given in ppm, coupling constants in Hz. Elemental analyses were performed by the microanalytical laboratory of our institute on a FlashSmart elemental analyzer from Thermo Scientific. Melting points were determined by using a Wagner and Munz PolyTherm A system and are not corrected. Mass spectra were recorded using an LC-MS coupled device by Hewlett-Packard (HPLC: 1100, mass detector: Esquire-LC, electrospray ionization).

### Computational Methods

The Gaussian16 ^38^ (Rev C.03) package was used to perform all quantum chemical calculations employing standard functionals, basis sets, and models. Detailed information on the computational settings along with literature references can be found below. All given ground state and excited state structures were verified as true minima by vibrational frequency analysis and the absence of imaginary vibrational frequencies and the absence of negative eigenvalues. UV/vis spectra and electronic transitions were calculated using TD-DFT methods (singlets, nstates =50, CPCM, solvent = dichloromethane) as implemented in the Gaussian16 package. The obtained excited state geometries were verified as true minima by vibrational frequency analysis. Calculated geometries were visualized and compared with GaussView^39^ and ChemCraft.^57^ The NTO analyses were carried out with the Multiwfn software package.^58^

### X-ray Diffraction

Preliminary examination and data collection for single crystals of compounds **5b** and **5e** were carried out on a Bruker D8 VENTURE (KAPPA goniometer, PHOTON detector) single crystal-diffractometer equipped with an Oxford Cryosystem (Cryostream 800) cooling system at the window of a sealed X-ray tube using monochromatic Mo–K_α_ (λ = 0.71073 Å) radiation (Incoatec IμS 3.0 microfocus source equipped with multilayer optics). Intensity data were extracted using the APEX3 suite^59^ including the SAINT software package.^60^ The reflections were merged and corrected from Lorentz, polarization, and decay effects, and an absorption correction was applied based on multiple scans.^61^ The structure was solved by a combination of dual space,^62^ direct methods with the aid of difference Fourier synthesis and were refined against all data using SHELXTL-XTMP. Hydrogen atoms were assigned to ideal positions using the SHELXTL-XTMP riding model. All non-hydrogen atoms were refined with anisotropic displacement parameters. Full-matrix least-squares refinements were carried out by minimizing Σw­(F_o_^2^-F_c_^2^)^2^ with the SHELXTL-XTMP weighting scheme. Neutral-atom scattering factors for all atoms and anomalous dispersion corrections for the non-hydrogen atoms were taken from the International Tables for Crystallography.^63^ All calculations were performed with the APEX3 suite^59^ including the SAINT software package,^60^ the SHELX program package,^64^ PLATON^65^ and Mercury.^66^

### Photoluminescence Measurements

The PMMA emitter films were prepared by doctor blading a solution of an emitter in a 10 wt % PMMA solution in dichloromethane on a quartz substrate with a 60 μm doctor blade. The film was dried and the emission was measured under a nitrogen atmosphere (including a nitrogen purge prior to sample measurement). Measurements at 77 K were conducted in degassed 2-methyltetrahydrofuran (analyte concentration: 5 × 10^–4^ mol/L) in a quartz cuvette. The solution was frozen, and inserted in a quartz finger dewar containing liquid nitrogen. Excitation was conducted in a wavelength range of 250–400 nm (Xe lamp with a monochromator), and the emission and quantum yields were measured with the absolute photoluminescence quantum yield spectrometer Quantaurus from Hamamatsu (model C11347-01) with an integrating sphere. Absorption spectra were measured on a PerkinElmer Lambda 365 UV/vis spectrometer in dichloromethane solutions with an analyte concentration of 2.5 × 10^–5^ mol/L. The phosphorescence decay times were measured on an Edinburgh Instruments mini-Tau. The excitation was carried out with pulses of an EPLED (360 nm, 20 kHz) and time-resolved photon counting (TCSPC).

### Syntheses

*N* ^2^-Phenylpyridine-2,3-diamine (**1a**) was synthesized according to a published procedure.^67^

#### 3-Nitro-*N*-(3-methylphenyl)­pyridine-2-amine (**1b**)

In a 500 mL round-bottom flask, 9.7 g (60 mmol, 1 equiv) of 2-chloro-3-nitropyridine and 6.6 mL (60 mmol, 1 equiv) of 3-methylaniline were dissolved in 100 mL of *n*-butanol and refluxed for 5 days. The solution was cooled to room temperature, during which an orange solid precipitated and was subsequently filtered off. The orange needles were washed with a small amount of cold ethanol and ether and dried under high vacuum. Yield 9.7 g (71%). ^1^H NMR (300 MHz, CDCl_3_) δ = 10.07 (s, 1H), 8.63–8.29 (m, 2H), 7.53–7.39 (m, 2H), 7.35–7.21 (m, 1H), 7.07–6.95 (m, 1H), 6.82 (dd, *J* = 8.3, 4.5 Hz, 1H), 2.39 (d, *J* = 0.7 Hz, 3H) ppm. ^13^C NMR (75 MHz, CDCl_3_) δ = 155.5, 150.6, 139.1, 137.8, 135.7, 129.0, 125.9, 125.0, 123.5, 120.0, 113.9, 21.7 ppm. ^13^C NMR DEPT-135 (75 MHz, CDCl_3_) δ = 155.5, 135.7, 129.0, 125.9, 123.5, 120.0, 113.9, 21.7 ppm. HRMS (ESI^+^) calcd [M + H]^+^ 230.0924 found 230.0930. m.p.: 80–84 °C.

#### 3-Nitro-*N*-(4-methylphenyl)­pyridine-2-amine (**1** **c**)

In a 500 mL round-bottom flask, 6 g (37 mmol, 1 equiv) of 2-chloro-3-nitropyridine and 4 g (37 mmol, 1 equiv) of 4-methylaniline were dissolved in 100 mL of isopropanol and refluxed for 5 days. The solution was cooled to room temperature, allowing an orange solid to precipitate. The solid was subsequently filtered off. The orange needles were washed with a small amount of cold ethanol and ether and dried *in vacuo*. Yield: 4 g (48%). ^1^H NMR (300 MHz, CDCl_3_) δ = 10.03 (s, 1H), 8.51 (dd, *J* = 8.3, 1.8 Hz, 1H), 8.46 (dd, *J* = 4.5, 1.8 Hz, 1H), 7.54–7.43 (m, 2H), 7.20 (d, *J* = 8.2 Hz, 1H), 6.79 (dd, *J* = 8.3, 4.5 Hz, 1H), 2.36 (s, 3H) ppm. ^13^C NMR (75 MHz, CDCl_3_) δ = 155.6, 150.7, 135.7, 135.2, 135.0, 129.7, 123.1, 123.0, 113.7, 21.1 ppm. ^13^C NMR DEPT-135 (75 MHz, CDCl_3_) δ = 155.6, 135.7, 129.7, 123.1, 113.7, 21.1 ppm. HRMS (ESI^+^) calcd [M + H]^+^ 230.0924 found 230.0931. m.p.: 61–63 °C.

#### *N*-(4-Ethoxyphenyl)-3-nitropyridine-2-amine (**1d**)

In a 100 mL round-bottom flask, 4.9 g (30 mmol, 1 equiv) of 2-chloro-3-nitropyridine and 3.9 mL (30 mmol, 1 equiv) of 4-ethoxyaniline were dissolved in 80 mL of isopropanol and refluxed for 24 h. The solution was left to cool, during which a red solid crystallized out and was subsequently filtered off. The red needles were washed with a small amount of cold isopropanol and dried *in vacuo*. Yield 5.5 g (71%). ^1^H NMR (300 MHz, CDCl_3_) δ = 9.96 (s, 1H), 8.50 (dd, *J* = 8.4, 1.8 Hz, 1H), 8.44 (dd, *J* = 4.5, 1.8 Hz, 1H), 7.54–7.41 (m, 2H), 7.00–6.87 (m, 2H), 6.77 (dd, *J* = 8.3, 4.5 Hz, 1H), 4.05 (q, *J* = 7.0 Hz, 2H), 1.42 (t, *J* = 7.0 Hz, 3H) ppm. ^13^C NMR (75 MHz, CDCl_3_) δ = 156.6, 155.7, 151.0, 135.7, 130.6, 128.4, 124.9, 124.8, 115.0, 113.5, 63.9, 15.0 ppm. ^13^C NMR DEPT-135 (75 MHz, CDCl_3_) δ = 155.7, 135.7, 124.9, 115.0, 113.5, 63.8, 15.0 ppm. HRMS (ESI^+^) calcd [M + H]^+^ 260.1030 found 260.1035. m.p.: 82–86 °C.

#### 3-Nitro-*N*-(4-(trifluormethoxy)­phenyl)­pyridine-2-amine (**1e**)

In a 100 mL round-bottom flask, 2.4 g (15 mmol, 1 equiv) of 2-chloro-3-nitropyridine and 2.1 mL (15 mmol, 1 equiv) of 4-(trifluoromethoxy)­aniline were dissolved in 40 mL of isopropanol and refluxed for 24 h. The solution was cooled to room temperature, when an orange solid crystallized out and was subsequently filtered off. The red needles were washed with a small amount of cold isopropanol and dried *in vacuo*. Yield: 2.7 g (59%). ^1^H NMR (300 MHz, CDCl_3_) δ = 10.13 (s, 1H), 8.54 (dd, *J* = 8.3, 1.8 Hz, 1H), 8.49 (dd, *J* = 4.5, 1.8 Hz, 1H), 7.73–7.65 (m, 2H), 7.28–7.19 (m, 2H), 6.88 (dd, *J* = 8.3, 4.5 Hz, 1H) ppm. ^13^C NMR (75 MHz, CDCl_3_) δ = 155.2, 150.1, 145.7 (q, *J*_C–F_ = 1.8 Hz), 136.7, 135.8, 128.9, 123.6, 121.8, 120.6 (q, *J*_C–F_ = 256.9 Hz), 114.5 ppm. ^13^C NMR DEPT-135 (75 MHz, CDCl_3_) δ = 155.2, 136.7, 135.8, 123.6, 121.8, 114.5 ppm. ^19^F NMR (282 MHz, CDCl_3_) δ = −58.1 ppm. HRMS (ESI^+^) calcd [M + H]^+^ 300.0591 found 300.0595. m.p.: 59–63 °C.

#### 3-Phenyl-3*H* *-*imidazo­[4,5-*b*]­pyridine (**2a**)

In a 100 mL round-bottom flask, 0.4 g (2.2 mmol, 1 equiv) of *N* ^2^-phenylpyridine-2,3-diamine (**1a**) was dissolved in 20 mL of formic acid and refluxed for 24 h. The reaction was then cooled to room temperature. The solvent was removed *in vacuo*, and the crude product was taken up in dichloromethane. The organic phase was washed with a saturated sodium bicarbonate solution. The organic phase was dried over magnesium sulfate, filtered, and dried under vacuum. The gray crude product was purified by column chromatography using pure ethyl acetate, yielding a red oil that later crystallized forming a brown solid. Yield: 189 mg (45%). ^1^H NMR (300 MHz, DMSO-*d*_6_) δ = 8.91 (s, 1H), 8.44 (dd, *J* = 4.8, 1.5 Hz, 1H), 8.22 (dd, *J* = 8.0, 1.5 Hz, 1H), 8.00–7.89 (m, 2H), 7.66–7.56 (m, 2H), 7.51–7.43 (m, 1H), 7.40 (dd, *J* = 8.0, 4.7 Hz, 1H) ppm. ^13^C NMR (75 MHz, DMSO-*d*_6_) δ = 146.4, 144.4, 144.2, 135.6, 135.2, 129.5, 128.0, 127.4, 123.1, 118.9 ppm. ^13^C NMR DEPT-135 (75 MHz, DMSO-*d*_6_) δ = 144.4, 144.2, 129.5, 128.0, 127.4, 123.1, 118.9 ppm. HRMS (ESI^+^) calcd [M + H]^+^ 196.0869 found 196.0882. m.p.: 40–44 °C.

#### 3-(3-Methylphenyl)-3*H*-imidazo­[4,5-*b*]­pyridine (**2b**)

In a 250 mL round-bottom flask, 2.5 g (10.9 mmol, 1 equiv) of 3-nitro-*N*-(3-methylphenyl)­pyridine-2-amine (**1b**), 6.7 g (120 mmol, 11 equiv) of iron powder, 6.4 g (120 mmol, 11 equiv) of ammonium chloride, and 40 mL (1.1 mol, 100 equiv) of formic acid were refluxed in 55 mL of isopropanol for 72 h. The reaction mixture was cooled to room temperature and then decanted into a round-bottom flask. The residue was rinsed with isopropanol. The solvents were removed using a rotary evaporator. The residue was diluted with a small amount of distilled water and adjusted to basic pH using a saturated sodium bicarbonate solution. The aqueous phase was extracted 3 times with 100 mL portions of dichloromethane. The combined organic phases were washed with a saturated sodium chloride solution, dried over magnesium sulfate, and filtered through a paper filter. The filtrate was dried *in vacuo*. The crude product was purified by column chromatography (ethyl acetate: iso-hexane 1:1 v/v). A brown oil was obtained. Yield 1.4 g (61%). ^1^H NMR (300 MHz, CDCl_3_) δ = 8.48–8.44 (m, 1H), 8.31 (s, 1H), 8.18–8.11 (m, 1H), 7.57–7.50 (m, 2H), 7.45 (td, *J* = 7.4, 1.0 Hz, 1H), 7.31 (dd, *J* = 8.0, 4.8 Hz, 1H), 7.28–7.22 (m, 1H), 2.47 (s, 3H) ppm. ^13^C NMR (75 MHz, CDCl_3_) δ = 147.1, 145.1, 143.4, 140.1, 135.2, 129.7, 129.0, 128.5, 124.5, 121.0, 119.0, 21.6 ppm. ^13^C NMR DEPT-135 (75 MHz, CDCl_3_) δ = 145.1, 143.4, 129.7, 129.0, 128.5, 124.5, 121.0, 119.0, 21.6 ppm. HRMS (ESI^+^) calcd [M + H]^+^ 210.1026 found 210.1040.

#### 3-(4-Methylphenyl)-3*H*-imidazo­[4,5-*b*]­pyridine (**2c**)

In a 250 mL round-bottom flask, 2.5 g (10.9 mmol, 1 equiv) of 3-nitro-*N*-(4-methylphenyl)­pyridine-2-amine (**1c**), 6.7 g (120 mmol, 11 equiv) of iron powder, 6.4 g (120 mmol, 11 equiv) of ammonium chloride, and 40 mL (1.1 mol, 100 equiv) of formic acid were refluxed in 55 mL of isopropanol for 72 h. The reaction mixture was cooled to room temperature and then decanted into a round-bottom flask. The residue was rinsed with isopropanol. The solvents were removed using a rotary evaporator. The residue was diluted with a small amount of distilled water and adjusted to basic pH using a saturated sodium bicarbonate solution. The aqueous phase was extracted 3 times with 100 mL portions of dichloromethane. The combined organic phases were washed with a saturated sodium chloride solution, dried over magnesium sulfate, and filtered through a paper filter. The filtrate was dried *in vacuo*. The crude product was purified by column chromatography (ethyl acetate: iso-hexane 1:1 v/v). A brown solid was obtained. Yield 0.9 g (40%). ^1^H NMR (300 MHz, CDCl_3_) δ = 8.44 (dd, *J* = 4.8, 1.3 Hz, 1H), 8.29 (s, 1H), 8.14 (dd, *J* = 8.1, 1.3 Hz, 1H), 7.60 (d, *J* = 8.3 Hz, 2H), 7.36 (d, *J* = 8.3 Hz, 2H), 7.29 (dd, *J* = 8.1, 4.8 Hz, 1H), 2.43 (s, 3H) ppm. ^13^C NMR (75 MHz, CDCl_3_) δ = 147.1, 145.0, 143.3, 138.1, 135.9, 132.7, 130.5, 128.4, 123.8, 118.9, 21.2 ppm. ^13^C NMR DEPT-135 (75 MHz, CDCl_3_) δ = 145.1, 143.3, 130.5, 128.4, 123.8, 118.9, 21.2 ppm. HRMS (ESI^+^) calcd [M + H]^+^ 210.1026 found 210.1038. m.p.: 67–70 °C.

#### 3-(4-Ethoxyphenyl)-3*H*-imidazo­[4,5-*b*]­pyridine (**2d**)

In a 250 mL round-bottom flask, 3 g (10.6 mmol, 1 equiv) of *N*-(4-ethoxyphenyl)-3-nitropyridin-2-amine (**1d**), 7 g (127 mmol, 11 equiv) of iron powder, 6.8 g (127 mmol, 11 equiv) of ammonium chloride, and 44 mL (1.16 mol, 100 equiv) of formic acid were refluxed in 55 mL of isopropanol for 48 h. The reaction mixture was cooled to room temperature and then decanted into a round-bottom flask. The residue was rinsed with isopropanol and the solvents were concentrated using a rotary evaporator. The residue was diluted with a small amount of distilled water and adjusted to basic pH using a saturated sodium bicarbonate solution. The aqueous phase was extracted 3 times with 100 mL portions of dichloromethane. The combined organic phases were washed with a saturated sodium chloride solution, dried over magnesium sulfate, and filtered through a paper filter. The filtrate was dried *in vacuo*. The crude product was purified by column chromatography (ethyl acetate: iso-hexane 1:2 → 1:1 → 2:1 v/v). A brown solid was obtained. Yield: 1.6 g (59%). ^1^H NMR (300 MHz, CDCl_3_) δ = 8.45 (dd, *J* = 4.8, 1.5 Hz, 1H), 8.26 (s, 1H), 8.15 (dd, *J* = 8.1, 1.5 Hz, 1H), 7.64–7.55 (m, 2H), 7.30 (dd, *J* = 8.1, 4.8 Hz, 1H), 7.12–7.03 (m, 2H), 4.10 (q, *J* = 7.0 Hz, 2H), 1.46 (t, *J* = 7.0 Hz, 3H) ppm. ^13^C NMR (75 MHz, CDCl_3_) δ = 158.8, 147.2, 145.1, 143.5, 135.8, 128.4, 127.9, 125.5, 118.9, 115.7, 64.1, 14.9 ppm. ^13^C NMR DEPT-135 (75 MHz, CDCl_3_) δ = 145.1, 143.5, 128.4, 125.5, 118.9, 115.7, 64.1, 14.9 ppm. HRMS (ESI^+^) calcd [M + H]^+^ 240.1131 found 240.1148. m.p.: 92–96 °C.

#### 3-(4-(Trifluormethoxy)­phenyl)-3*H*-imidazo­[4,5-*b*]­pyridine (**2e**)

In a 250 mL round-bottom flask, 2 g (6.68 mmol, 1 equiv) of 3-nitro-*N*-(4-(trifluoromethoxy)­phenyl)-pyridin-2-amine (**1e**), 4.1 g (73.5 mmol, 11 equiv) of iron powder, 3.9 g (73.5 mmol, 11 equiv) of ammonium chloride, and 25 mL (0.67 mol, 100 equiv) of formic acid were refluxed in 55 mL of isopropanol for 48 h. The reaction mixture was cooled to room temperature and then decanted into a round-bottom flask. The residue was rinsed with isopropanol and the solvent was concentrated using a rotary evaporator. The residue was diluted with a small amount of distilled water and adjusted to basic pH using a saturated sodium bicarbonate solution. The aqueous phase was extracted 3 times with 100 mL portions of dichloromethane. The combined organic phases were washed with a saturated sodium chloride solution, dried over magnesium sulfate, and filtered through a folded filter. The filtrate was dried *in vacuo*. The crude product was purified by column chromatography (ethyl acetate: iso-hexane 1:1 v/v). A brown solid was obtained. Yield: 1.7 g (92%). ^1^H NMR (300 MHz, CDCl_3_) δ = 8.47 (dd, *J* = 4.8, 1.5 Hz, 1H), 8.34 (s, 1H), 8.18 (dd, *J* = 8.1, 1.5 Hz, 1H), 7.90–7.73 (m, 2H), 7.51–7.40 (m, 2H), 7.34 (dd, *J* = 8.1, 4.8 Hz, 1H) ppm. ^13^C NMR (75 MHz, CDCl_3_) δ = 148.5 (d, *J*_C–F_ = 2.1 Hz), 146.8, 145.3, 142.8, 136.0, 133.8, 128.8, 125.1, 122.6, 120.6 (d, *J*_C–F_ = 258.0 Hz), 119.4 ppm. ^13^C NMR DEPT-135 (75 MHz, CDCl_3_) δ = 148.5 (d, *J*_C–F_ = 2.1 Hz), 146.8, 145.3, 142.8, 136.0, 133.8, 128.8, 125.1, 122.6, 120.6 (d, *J*_C–F_ = 258.0 Hz), 119.4 ppm. ^19^F NMR (282 MHz, CDCl_3_) δ = −58.0 ppm. HRMS (ESI^+^) calcd [M + H]^+^ 280.0692 found 280.0702. m.p.: 73–78 °C.

#### 1-Methyl-3-phenyl-3*H*-imidazo­[4,5-*b*]­pyridine-1-ium iodide (**3a**)

In a pressure tube, 1.8 g (9.22 mmol, 1 equiv) of 3-phenyl-3*H*-imidazo­[4,5-*b*]­pyridine (**2a**) was suspended in 7 mL of THF, then 1.2 mL (18.4 mmol, 2 equiv) of methyl iodide was added via syringe and the mixture was stirred for 24 h at 90 °C. The reaction mixture was cooled to room temperature. After adding ether, a beige solid precipitated, which was then filtered off and washed with ether and a small amount of THF. The solid was then dried *in vacuo*. Yield: 3.1 g (98%). ^1^H NMR (300 MHz, DMSO-*d*_6_) δ = 10.43 (s, 1H), 8.82 (dd, *J* = 4.7, 1.3 Hz, 1H), 8.71 (d, *J* = 8.3 Hz, 1H), 7.96–7.91 (m, 2H), 7.89 (dd, *J* = 8.4, 4.8 Hz, 1H), 7.81–7.60 (m, 3H), 4.21 (s, 3H) ppm. ^13^C NMR (75 MHz, DMSO-*d*_6_) δ = 148.7, 144.2, 142.8, 132.4, 130.2, 129.9, 125.3, 125.1, 123.8, 122.6, 34.2 ppm. ^13^C NMR DEPT-135 (75 MHz, DMSO-*d*_6_) δ = 148.7, 144.2, 130.2, 129.9, 125.1, 123.8, 122.6, 34.2 ppm.

#### 1-Methyl-3-(3-methylphenyl)-3*H*-imidazo­[4,5-*b*]­pyridin-1-ium­(**3b**)

In a pressure tube, 1 g (4.78 mmol, 1 equiv) of 3-(3-methylphenyl)-3*H*-imidazo­[4,5-*b*]-pyridine (**2b**) was suspended in 5 mL of THF, then 0.6 mL (9.56 mmol, 2 equiv) of methyl iodide was added via syringe, and the mixture was stirred for 24 h at 90 °C. The reaction mixture was cooled to room temperature. After adding ether, a beige solid precipitated, which was then filtered off and washed with ether and a small amount of THF. The solid was then dried *in vacuo*. Yield: 1.57 g (94%). ^1^H NMR (300 MHz, DMSO-*d*_6_) δ = 10.37 (s, 1H), 8.82 (dd, *J* = 4.7, 1.4 Hz, 1H), 8.69 (dd, *J* = 8.4, 1.3 Hz, 1H), 7.88 (dd, *J* = 8.4, 4.7 Hz, 1H), 7.76–7.69 (m, 2H), 7.62 (td, *J* = 7.6, 0.9 Hz, 1H), 4.19 (s, 3H), 2.46 (s, 3H) ppm. ^13^C NMR (75 MHz, DMSO-*d*_6_) δ = 148.7, 144.2, 142.7, 139.7, 132.4, 130.8, 129.8, 125.3, 125.3, 123.8, 122.5, 122.1, 34.1, 20.9 ppm. ^13^C NMR DEPT-135 (75 MHz, DMSO-*d*_6_) δ = 148.7, 144.2, 130.8, 129.8, 125.3, 123.8, 122.5, 122.2, 34.1, 20.9 ppm. HRMS (ESI^+^) calcd [M–I]^+^ 224.1182 found 224.1191. m.p.: 195–199 °C.

#### 1-Methyl-3-(4-methylphenyl)-3*H*-imidazo­[4,5-*b*]­pyridin-1-ium (**3c**)

In a pressure tube, 0.87 g (4.17 mmol, 1 equiv) of 3-(4-methylphenyl)-3*H*-imidazo­[4,5-*b*]-pyridine (**2c**) was suspended in 5 mL of THF, then 0.5 mL (8.34 mmol, 2 equiv) of methyl iodide was added via syringe, and the mixture was stirred for 24 h at 90 °C. The reaction mixture was cooled to room temperature. After adding ether, a beige solid precipitated, which was then filtered off and washed with ether and a small amount of THF. The solid was then dried *in vacuo*. Yield: 1.33 g (91%). ^1^H NMR (300 MHz, DMSO-*d*_6_) δ = 10.36 (s, 1H), 8.81 (dd, *J* = 4.7, 1.4 Hz, 1H), 8.69 (dd, *J* = 8.4, 1.4 Hz, 1H), 7.87 (dd, *J* = 8.4, 4.8 Hz, 1H), 7.84–7.77 (m, 2H), 7.62–7.47 (m, 2H), 4.19 (s, 3H), 2.45 (s, 3H) ppm. ^13^C NMR (75 MHz, DMSO-*d*_6_) δ = 148.6, 144.1, 142.8, 140.0, 130.3, 129.9, 125.2, 124.8, 123.7, 122.5, 34.0, 20.8 ppm. ^13^C NMR DEPT-135 (75 MHz, DMSO-*d*_6_) δ = 148.6, 144.1, 130.2, 124.8, 123.7, 122.5, 34.0, 20.8 ppm. HRMS (ESI^+^) calcd [M–I]^+^ 224.1182 found 224.1193. m.p.: 271–274 °C.

#### 3-(4-Ethoxyphenyl)-1-methyl-3*H*-imidazo­[4,5-*b*]­pyridin-1-iumiodide (**3d**)

In a pressure tube, 0.8 g (3.34 mmol, 1 equiv) of 3-(4-ethoxyphenyl)-3*H*-imidazo­[4,5-*b*]-pyridine (**2d**) was suspended in 5 mL of THF, then 0.4 mL (6.69 mmol, 2 equiv) of methyl iodide was added via syringe, and the mixture was stirred for 24 h at 90 °C. The reaction mixture was cooled to room temperature. After adding ether, a gray solid precipitated, which was then filtered off and washed with ether and a small amount of THF. The solid was dried *in vacuo*. Yield: 1.23 g (97%). ^1^H NMR (300 MHz, DMSO-*d*_6_) δ = 10.30 (s, 1H), 8.80 (dd, *J* = 4.7, 1.4 Hz, 1H), 8.67 (dd, *J* = 8.4, 1.4 Hz, 1H), 7.87 (dd, *J* = 8.4, 4.7 Hz, 1H), 7.83–7.75 (m, 2H), 7.31–7.20 (m, 2H), 4.18 (s, 3H), 4.14 (q, *J* = 7.0 Hz, 2H), 1.39 (t, *J* = 7.0 Hz, 3H) ppm. ^13^C NMR (75 MHz, DMSO-*d*_6_) δ = 159.5, 148.5, 144.1, 142.9, 126.6, 125.1, 124.9, 123.6, 122.4, 115.4, 63.7, 34.0, 14.5 ppm. ^13^C NMR DEPT-135 (75 MHz, DMSO-*d*_6_) δ = 148.5, 144.1, 126.6, 123.6, 122.4, 115.4, 63.7, 34.0, 14.5 ppm. Anal. calcd for C_15_H_16_N_3_OI: C, 47.26%; H, 4.23%; N, 11.02%. Found C, 47.45%; H, 4.10%; N, 10.84%. HRMS (ESI^+^) calcd [M–I]^+^ 254.1288 found 254.1300. m.p.: 276–279 °C.

#### 3-(4-(Trifluormethoxy)­phenyl)-1-methyl-3*H*-imidazo­[4,5-*b*]­pyridin-1-iumiodide (**3e**)

In a pressure tube, 1.16 g (4.17 mmol, 1 equiv) of 3-(4-(trifluoromethoxy)­phenyl)-3*H*-imidazo­[4,5-*b*]­pyridine (**2e**) was suspended in 8 mL of THF, then 0.5 mL (8.34 mmol, 2 equiv) of methyl iodide was added via syringe, and the mixture was stirred for 24 h at 90 °C. The reaction mixture was then cooled to room temperature. While adding ether, a gray solid precipitated, which was then filtered off and washed with ether and a small amount of THF. The solid was dried *in vacuo*. Yield 1.8 g (quant.). ^1^H NMR (300 MHz, DMSO-*d*_6_) δ = 10.43 (s, 1H), 8.82 (dd, *J* = 4.8, 1.4 Hz, 1H), 8.71 (dd, *J* = 8.4, 1.4 Hz, 1H), 8.15–8.00 (m, 2H), 7.90 (dd, *J* = 8.4, 4.8 Hz, 1H), 7.84–7.75 (m, 2H), 4.22 (s, 3H) ppm. ^13^C NMR (75 MHz, DMSO-*d*_6_) δ = 148.9, 148.7, 144.4, 142.7, 131.3, 127.4, 125.2, 123.9, 122.7, 122.6, 120.0 (q, *J*_C–F_ = 257.5 Hz), 34.2 ppm. ^13^C NMR DEPT-135 (75 MHz, DMSO-*d*_6_) δ = 148.7, 127.4, 123.9, 122.7, 122.6, 34.2 ppm. ^19^F NMR (282 MHz, DMSO-*d*_6_) δ = −58.2 ppm. Anal. calcd for C_14_H_11_F_3_N_3_OI: C, 39.93%; H, 2.63%; N, 9.98%; Found: C, 40.01%; H, 2.53%; N, 9.80%; HRMS (ESI^+^) calcd [M–I]^+^ 294.0849 found 294.0855. m.p.: 225–229 °C.

#### General Procedure for the Synthesis of μ-Chloro Precursors (**4a**–**4e**)

In an oven-dried Schlenk tube, 0.55 equiv of silver­(I) oxide were suspended together with 1 equiv of imidazolium salt (**3a**–**3e**) in dry dichloromethane (0.04 M) and stirred in the dark for 1 h at room temperature. One equivalent of dichloro­(1,5-cyclooctadiene)-platinum­(II) was added, and the mixture was stirred for another 18 h. The reaction mixture was filtered through diatomaceous earth and evaporated. The residue was dried under vacuum and transferred into a Schlenk tube connected to a reflux condenser. The atmospheres were exchanged three times with argon. Then, dry ethoxyethanol (0.06 M) was added, and the mixture was boiled for 3 h. The reaction was cooled to room temperature and filtered through a glass frit. The filter cake was washed with dichloromethane and ether and used further without further purification for the syntheses of platinum complexes.

#### Complex **5** **a**

In a Schlenk tube, 200 mg (0.23 mmol, 0.5 equiv) of **4a** and 147 mg (0.46 mmol, 1 equiv) of potassium dihydrobis­(3-(trifluoromethyl)-1*H*-pyrazol-1-yl)­borate (**6**) were placed under argon and suspended in 10 mL of dry dichloromethane. The mixture was stirred for 24 h at room temperature. The suspension was then filtered through diatomaceous earth. The filtrate was dried *in vacuo*. The residue was purified by column chromatography (DCM: isohexane 1:2 → 1:1). The resulting yellow solid was dried *in vacuo*. Yield: 237 mg (76%). ^1^H NMR (600 MHz, DCM-*d*_2_) δ = 8.52 (dd, *J* = 4.9, 1.4 Hz, 1H), 8.40 (dd, *J* = 7.8, 1.2 Hz, 1H), 7.77 (ddd, *J* = 19.4, 2.4, 0.8 Hz, 2H), 7.71 (dd, *J* = 8.1, 1.4 Hz, 1H), 7.34 (dd, *J* = 8.1, 4.9 Hz, 1H), 7.19 (td, *J* = 7.6, 1.4 Hz, 1H), 7.00–6.96 (m, 1H), 6.91 (td, *J* = 7.4, 1.3 Hz, 1H), 6.63 (dd, *J* = 6.9, 2.3 Hz, 2H), 5.07–4.37 (bs, 1H), 3.94–3.31 (bs, 1H), 3.52 (s, 3H) ppm. ^13^C NMR (151 MHz, DCM-*d*_2_) δ = 167.6, 147.7, 145.9, 145.5, 143.2 (q, *J*_C–F_ = 38.8 Hz), 142.9 (q, *J*_C–F_ = 38.6 Hz), 139.2, 139.1, 136.0, 128.4, 126.9, 125.3, 124.5, 121.3 (q, *J*_C–F_ = 270.3 Hz), 121.0 (q, *J*_C–F_ = 269.9 Hz), 119.3, 119.1, 114.6, 107.0 (d, *J*_C–F_ = 2.8 Hz), 106.8 (d, *J*_C–F_ = 2.5 Hz), 34.2 ppm. ^13^C NMR DEPT-135 (151 MHz, DCM-*d*_2_) δ = 145.9, 139.2, 139.1, 136.0, 125.3, 124.6, 119.3, 119.1, 114.6, 107.0 (d, *J*_C–F_ = 2.5 Hz), 106.8 (d, *J*_C–F_ = 2.4 Hz), 34.2 ppm. ^19^F NMR (282 MHz, DCM-*d*_2_) δ = −57.7, −60.6 ppm. ^195^Pt NMR (129 MHz, DCM-*d*_2_) δ = −3819.9 ppm. Anal. calcd for C_21_H_16_BF_6_N_7_Pt: C, 36.75%; H, 2.35%; N, 14.29%. Found C, 36.93%; H, 2.23; N, 14.41%; HRMS (ESI^+^) calcd [M + H]^+^ 687.1185 found 687.1182. m.p.: Decomposition at 233 °C.

#### Complex **5b**

In a Schlenk tube, 141 mg (0.16 mmol, 0.5 equiv) of **4b** and 100 mg (0.31 mmol, 1 equiv) of potassium dihydrobis­(3-(trifluoromethyl)-1*H*-pyrazol-1-yl)­borate (**6**) were placed under argon and suspended in 10 mL of dry dichloromethane. The mixture was stirred for 24 h at room temperature. The suspension was then filtered through a plug of SiO_2_ and eluted with 50 mL of dichloromethane. The solvent was removed, and the resulting yellow solid was dried *in vacuo*. Yield: 136 mg (63%). ^1^H NMR (600 MHz, DCM-*d*_2_) δ = 8.53 (dd, *J* = 4.9, 1.4 Hz, 1H), 8.27 (d, *J* = 1.6 Hz, 1H), 7.77 (ddd, *J* = 20.7, 2.3, 0.8 Hz, 2H), 7.72 (d, *J* = 1.4 Hz, 1H), 7.34 (dd, *J* = 8.1, 4.9 Hz, 1H), 6.84 (dd, *J* = 7.6, 1.4 Hz, 1H), 6.74 (ddd, *J* = 7.7, 1.8, 0.8 Hz, 1H), 6.62 (dd, *J* = 4.8, 2.4 Hz, 2H), 5.04–4.38 (bs, 1H), 3.93–3.12 (bs, 1H), 3.52 (s, 3H), 2.40 (s, 3H) ppm. ^13^C NMR (151 MHz, DCM-*d*_2_) δ = 167.9, 147.8, 145.9, 145.5, 143.3–142.5 (m), 143.0–142.0 (m), 139.1, 139.0, 135.7, 135.2, 128.5, 125.2, 123.2–121.6 (m), 121.0–120.2 (m), 119.2, 119.0, 115.5, 106.97 (d, *J*_C–F_ = 2.2 Hz), 106.8 (d, *J*_C–F_ = 2.5 Hz), 34.2, 21.7 ppm. ^13^C NMR DEPT-135 (151 MHz, DCM-*d*_2_) δ = 145.9, 139.1, 139.0, 135.7, 125.2, 119.2, 119.0, 115.5, 107.0 (d, *J*_C–F_ = 2.2 Hz), 106.8 (d, *J*_C–F_ = 2.5 Hz), 34.2, 21.7 ppm. ^19^F NMR (565 MHz, DCM-*d*_2_) δ = −57.6, −60.7 ppm. ^195^Pt NMR (129 MHz, DCM-*d*_2_) δ = 3825.6 ppm. Anal. calcd for C_22_H_18_BF_6_N_7_Pt: C, 37.73%; H, 2.59%; N, 14.00%. Found: C, 37.75%; H, 2.47%; N, 13.96%; HRMS (ESI^+^) calcd [M + H]^+^ 701.1341 found 701.1341. m.p.: Decomposition at 258 °C.

#### Complex **5c**

In a Schlenk tube, 141 mg (0.16 mmol, 0.5 equiv) of **4c** and 100 mg (0.31 mmol, 1 equiv) of potassium dihydrobis­(3-(trifluoromethyl)-1*H*-pyrazol-1-yl)­borate (**6**) were added under argon and suspended in 10 mL of dry dichloromethane. The reaction mixture was stirred at room temperature for 24 h. The suspension was then filtered through a plug of SiO_2_ and eluted with 50 mL of dichloromethane. The solvent was removed, and the resulting yellow solid was dried *in vacuo*. Yield 129 mg (59%). ^1^H NMR (600 MHz, DCM-*d*_2_) δ = 8.50 (d, *J* = 4.9 Hz, 1H), 8.25 (d, *J* = 7.9 Hz, 1H), 7.82–7.75 (m, 2H), 7.69 (d, *J* = 8.1 Hz, 1H), 7.32 (dd, *J* = 8.0, 4.9 Hz, 1H), 7.02–6.98 (m, 1H), 6.82 (s, 1H), 6.64 (dd, *J* = 14.4, 2.3 Hz, 2H), 5.03–4.25 (bs, 1H), 3.96–3.21 (bs, 1H), 3.51 (s, 3H), 2.25 (s, 3H) ppm. ^13^C NMR (151 MHz, DCM-*d*_2_) δ = 167.3, 145.9, 145.4, 145.3, 143.6–142.4 (m), 139.1, 139.0, 136.8, 134.1, 128.4, 126.8, 125.5, 121.3 (q, *J*_C–F_ = 269.2 Hz), 121.0 (q, *J*_C–F_ = 270.2 Hz), 119.2, 119.0, 114.2, 107.1–107.0 (m), 106.9–106.7 (m), 34.1, 21.3 ppm. ^13^C NMR DEPT-135 (151 MHz, DCM-*d*_2_) δ = 145.9, 139.1, 139.0, 136.8, 125.5, 119.1, 119.0, 114.2, 107.2–107.0 (m), 106.8 (d, *J*_C–F_ = 2.2 Hz), 34.1, 21.3 ppm. ^19^F NMR (565 MHz, DCM-*d*_2_) δ = −57.6, −60.6 ppm. ^195^Pt NMR (129 MHz, DCM-*d*_2_) δ = −3818.7 ppm. Anal. calcd for C_22_H_18_BF_6_N_7_Pt: C, 37.73%; H, 2.59%; N, 14.00%. Found: C, 37.89%; H, 2.39%; N, 13.78%. HRMS (ESI^+^) calcd [M + H]^+^ 701.1341 found 701.1344. m.p.: Decomposition at 220 °C.

#### Complex **5d**

In a Schlenk tube, 150 mg (0.16 mmol, 0.5 equiv) of **4d** and 100 mg (0.31 mmol, 1 equiv) of potassium dihydrobis­(3-(trifluoromethyl)-1*H*-pyrazol-1-yl)­borate (**6**) were added under argon and suspended in 10 mL of dry dichloromethane. The reaction mixture was stirred at room temperature for 24 h. The suspension was then filtered through a plug of SiO_2_ and eluted with 50 mL of dichloromethane. The solvent was removed, and the resulting yellow solid was dried *in vacuo*. Yield: 70 mg (31%). ^1^H NMR (600 MHz, DCM-*d*_2_) δ = 8.50 (dd, *J* = 4.9, 1.4 Hz, 1H), 8.30 (d, *J* = 8.5 Hz, 1H), 7.80–7.74 (m, 2H), 7.69 (dd, *J* = 8.1, 1.4 Hz, 1H), 7.31 (dd, *J* = 8.1, 4.9 Hz, 1H), 6.71 (dd, *J* = 8.6, 2.7 Hz, 1H), 6.63 (dd, *J* = 12.8, 2.3 Hz, 2H), 6.52 (dd, *J* = 2.6, 1.3 Hz, 1H), 5.07–4.36 (bs, 1H), 4.05–3.88 (m, 2H), 3.78–3.26 (bs, 1H), 3.50 (s, 3H), 1.33 (t, *J* = 7.0 Hz, 3H) ppm. ^13^C NMR (151 MHz, DCM-*d*_2_) δ = 166.2, 155.9, 145.8, 145.1, 143.2, 142.8, 141.3, 139.2, 139.1, 128.4, 128.3, 123.0, 121.3 (q, *J*_C–F_ = 269.4 Hz), 121.0 (q, *J*_C–F_ = 170.1 Hz) 119.1, 119.0, 115.0, 110.0, 107.1 (d, *J*_C–F_ = 2.2 Hz), 106.8, 64.0, 34.1, 15.3 ppm. ^13^C NMR DEPT-135 (151 MHz, DCM-*d*_2_) δ = 145.8, 139.2, 139.1, 123.0, 119.1, 119.0, 115.0, 110.0, 107.1 (d, *J*_C–F_ = 2.2 Hz), 106.8, 64.0, 34.1 (d, *J*_C–F_ = 1.9 Hz), 15.3 ppm. ^19^F NMR (565 MHz, DCM-*d*_2_) δ = −57.5, −60.6 ppm. ^195^Pt NMR (129 MHz, DCM-*d*_2_) δ = −3799.7 ppm. Anal. calcd for C_23_H_20_BF_6_N_7_OPt: C, 37.83%; H, 2.76%; N, 13.42%. Found: C, 38.21%; H, 2.62%; N, 12.99%; HRMS (ESI^+^) calcd [M + H]^+^ 731.1447 found 731.1447. m.p.: Decomposition at 207 °C.

#### Complex **5e**

In a Schlenk tube, 162 mg (0.16 mmol, 0.5 equiv) of **4e** and 100 mg (0.31 mmol, 1 equiv) of potassium dihydrobis­(3-(trifluoromethyl)-1*H*-pyrazol-1-yl)­borate (**6**) were added under argon, suspended in 10 mL of dry dichloromethane, and stirred at room temperature for 24 h. The suspension was then filtered through a plug of SiO_2_ and eluted with 50 mL of dichloromethane. The solvent was removed, and the resulting yellow solid was dried *in vacuo*. Yield: 172 mg (72%). ^1^H NMR (600 MHz, DCM-*d*_2_) δ = 8.53 (d, *J* = 4.8 Hz, 1H), 8.46 (d, *J* = 8.6 Hz, 1H), 7.81–7.76 (m, 2H), 7.73 (d, *J* = 8.1 Hz, 1H), 7.36 (dd, *J* = 8.1, 4.9 Hz, 1H), 7.08 (ddd, *J* = 8.6, 2.5, 1.1 Hz, 1H), 6.83 (s, 1H), 6.65 (dd, *J* = 12.2, 2.3 Hz, 2H), 5.06–4.32 (bs, 1H), 3.98–3.15 (bs, 1H), 3.53 (s, 3H) ppm. ^13^C NMR (151 MHz, DCM-*d*_2_) δ = 166.5, 146.3, 146.0, 145.2, 145.1, 143.4, 143.1, 142.8, 139.2, 139.1, 129.3, 128.4, 128.2, 121.8 (q, *J*_C–F_ = 255.8 Hz) 121.0 (q, *J*_C–F_ = 269.3 Hz), 120.6 (q, *J*_C–F_ = 270.0 Hz) 119.4, 119.1, 117.4, 114.8, 107.1 (d, *J*_C–F_ = 2.2 Hz), 106.8 (d, *J*_C–F_ = 2.2 Hz), 34.1 ppm. ^13^C NMR DEPT-135 (151 MHz, DCM-*d*_2_) δ = 146.0, 139.2, 139.1, 128.4, 119.4, 119.1, 117.4, 114.8, 107.1 (d, *J*_C–F_ = 2.3 Hz), 106.8, 34.1 ppm. ^19^F NMR (565 MHz, DCM-*d*_2_) δ = −57.9, −58.0, −60.6 ppm. ^195^Pt NMR (129 MHz, DCM-*d*_2_) δ = −3788.2 ppm. Anal. calcd for C_22_H_15_BF_9_N_7_OPt: C, 34.30%; H, 1.96%; N, 12.73%. Found: C, 34.44%; H, 1.97%; N, 12.72%; HRMS (ESI^+^) calcd [M + H]^+^ 771.1008 found 771.1003. m.p.: Decomposition at 210 °C.

#### Potassium Dihydrobis­(3-(trifluoromethyl)-1*H*-pyrazol-1-yl)­borate (**6**)

In a predried Schlenk tube, 1.3 g (25 mmol, 1 equiv) of finely ground potassium borohydride and 13.6 g (0.1 mol, 4 equiv) of freshly sublimed 3-(trifluoromethyl)­pyrazole were suspended in 5 mL of dry toluene under an argon atmosphere. The suspension was refluxed for 6 h, during which gas evolution was observed. When no further gas evolution was observed after 6 h, the reaction mixture was cooled to room temperature. A colorless solid was precipitated from the reaction solution by the addition of *n*-hexane, filtered off, and washed with *n*-hexane. The crude product was then recrystallized from a mixture of dichloromethane and *n*-hexane (1:1 v/v). Colorless, hygroscopic needles were obtained. Yield: 3.3 g (41%). ^1^H NMR (300 MHz, CDCl_3_) δ = 7.52 (dd, *J* = 2.1, 1.1 Hz, 2H), 6.43–6.34 (m, 2H), 3.71 (bs, 1H), 3.36 (bs, 1H) ppm. ^13^C NMR (75 MHz, CDCl_3_) δ = 142.6 (q, *J*_C–F_ = 35.9 Hz), 137.0, 122.5 (q, *J*_C–F_ = 267.5 Hz), 103.5 ppm. ^13^C NMR DEPT-135 (75 MHz, CDCl_3_) δ = 137.0, 103.5 ppm. ^19^F NMR (282 MHz, CDCl_3_) δ = −60.8 ppm. HRMS (ESI^–^) calcd [M–K]^−^ 283.0595 found 283.0601. m.p.: 122–126 °C.

## Supplementary Material



## Notes

NMRs, FMOs, and spin densities of compounds, details of the solid-state structure determination, and *xyz* coordinates of the optimized geometries (PDF)
