# Supplementary material for: Synthesis and Characterization of Platinum(II) Complexes with Bis(3-(trifluoromethyl)-pyrazolyl)-borate Auxiliary Ligands
Source: Inorg Chem. 2025 Jul 30;64(31):15881–91. doi: 10.1021/acs.inorgchem.5c01716 (PMC12344770; doi:10.1021/acs.inorgchem.5c01716)
Supplement: Supplementary file 1 [file ic5c01716_si_001.pdf]

Supporting Information

Synthesis and Characterization of Platinum(II) Complexes with Bis(3-(trifluoromethyl)-pyrazolyl) Borate Auxiliary Ligands

Tim Riesebeck and Thomas Strassner*

Physikalische Organische Chemie, Technische Universität Dresden, 01069 Dresden, Germany

*Prof. Dr. Thomas Strassner
Tel: (+49) 351-463-38571
E-mail: thomas.strassner@tu-dresden.de

Table of Contents

NMR Spectra S3

Solid State Structures S44

Computational Details S50

NMR Spectra

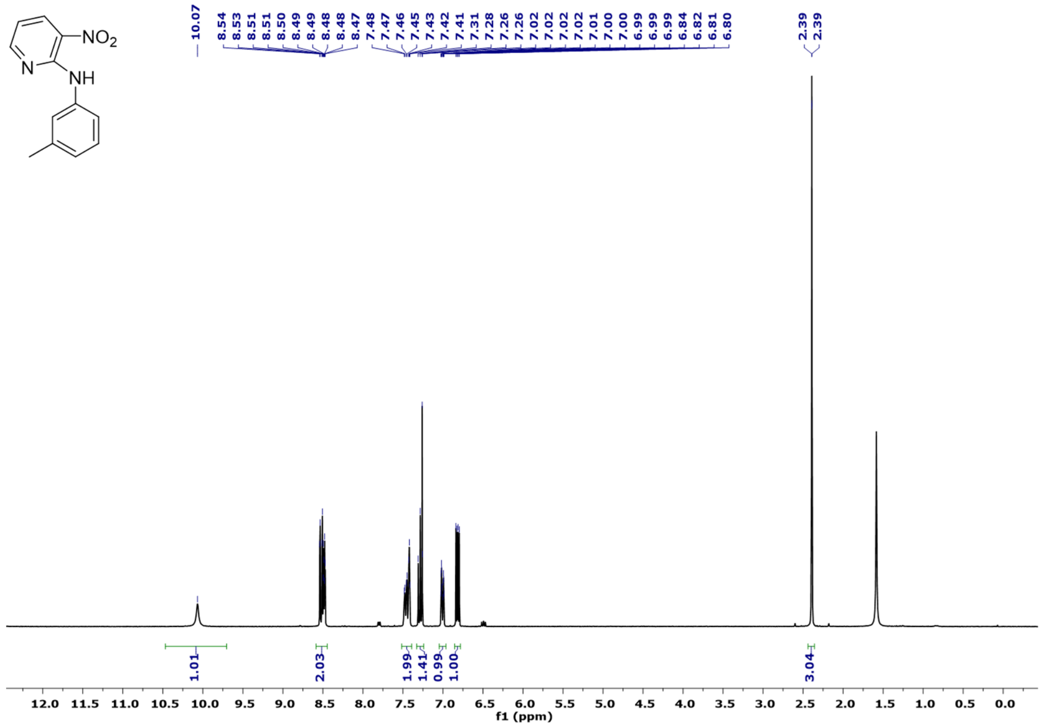

Figure S1: ^{1}H NMR spectrum (300 MHz) of **1b** in $CDCl_3$ at room temperature.

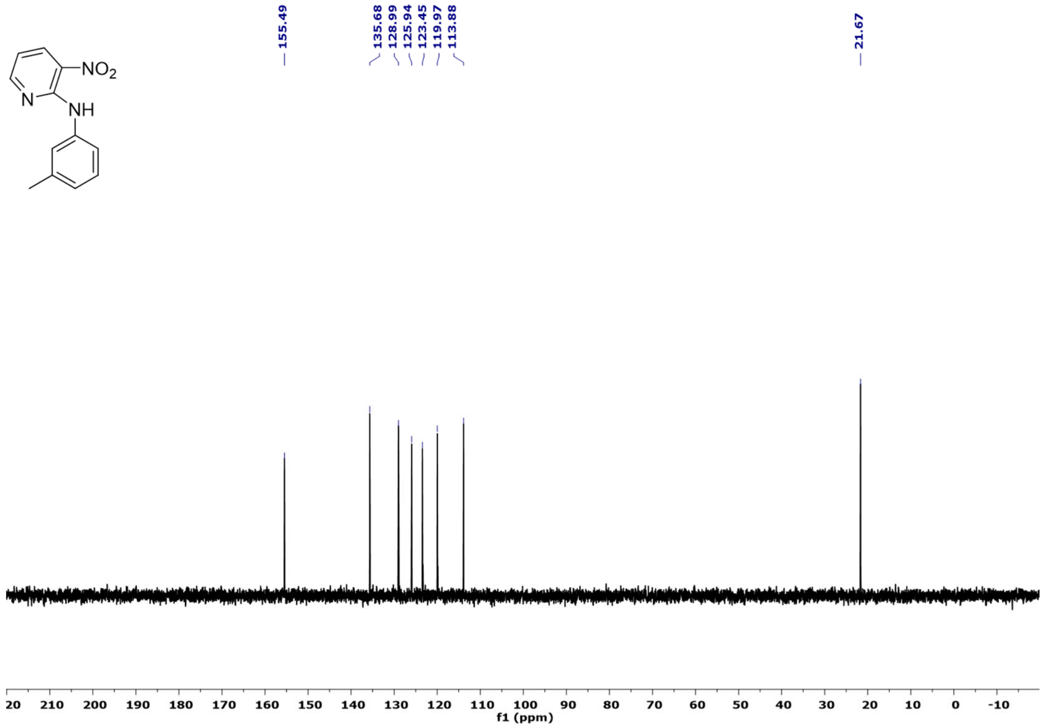

Figure S2: ^{13}C NMR spectrum (75 MHz) of **1b** in $CDCl_3$ at room temperature.

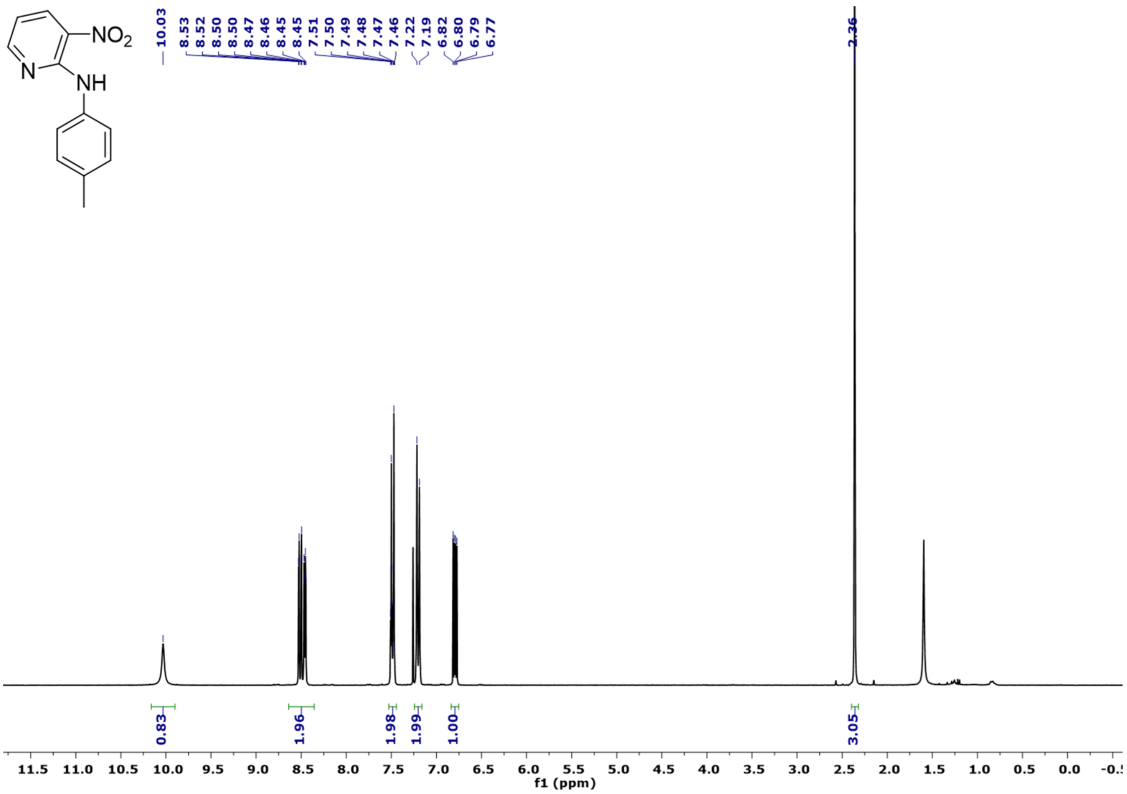

Figure S3: ^{1}H NMR spectrum (300 MHz) of **1c** in $CDCl_3$ at room temperature.

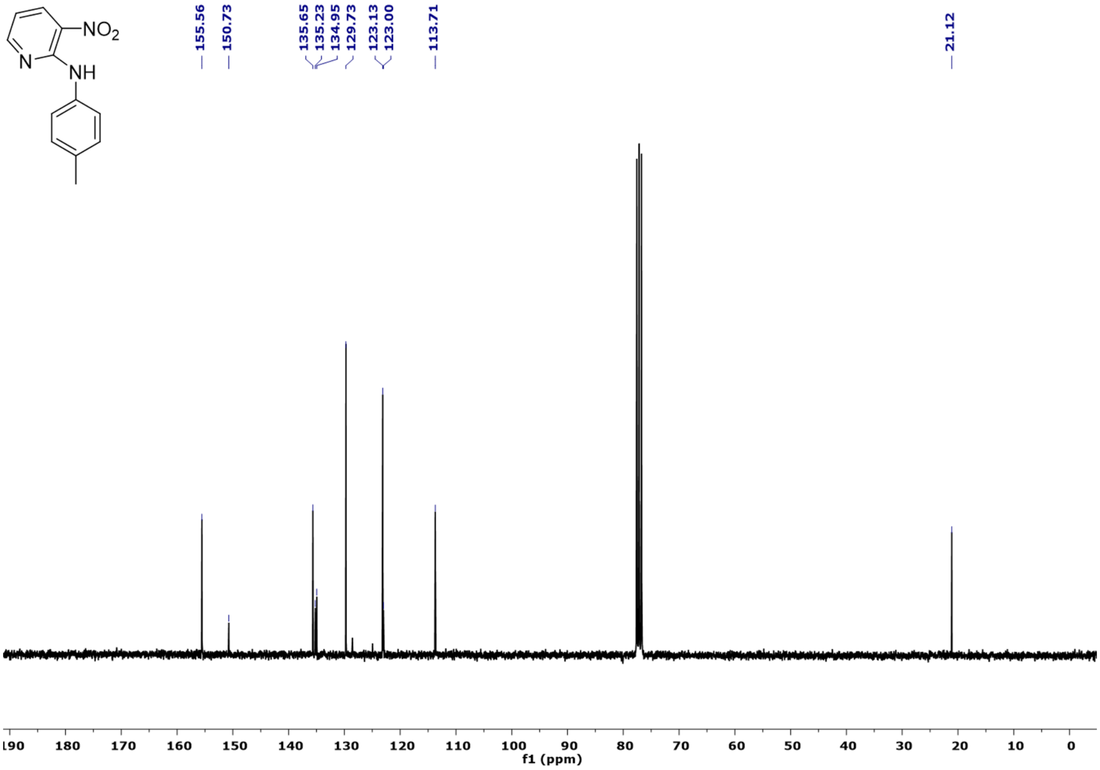

Figure S4: ^{13}C NMR spectrum (75 MHz) of **1c** in $CDCl_3$ at room temperature.

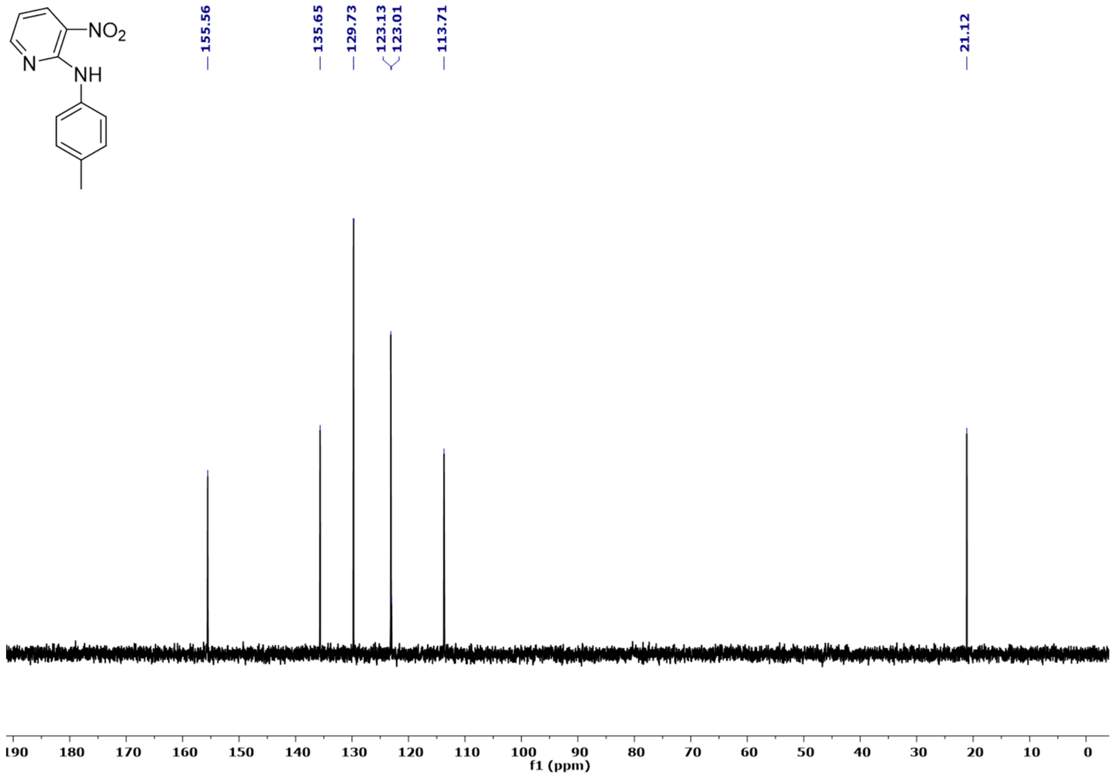

Figure S5: ^{13}C NMR (DEPT-135) spectrum (75 MHz) of **1c** in $CDCl_3$ at room temperature.

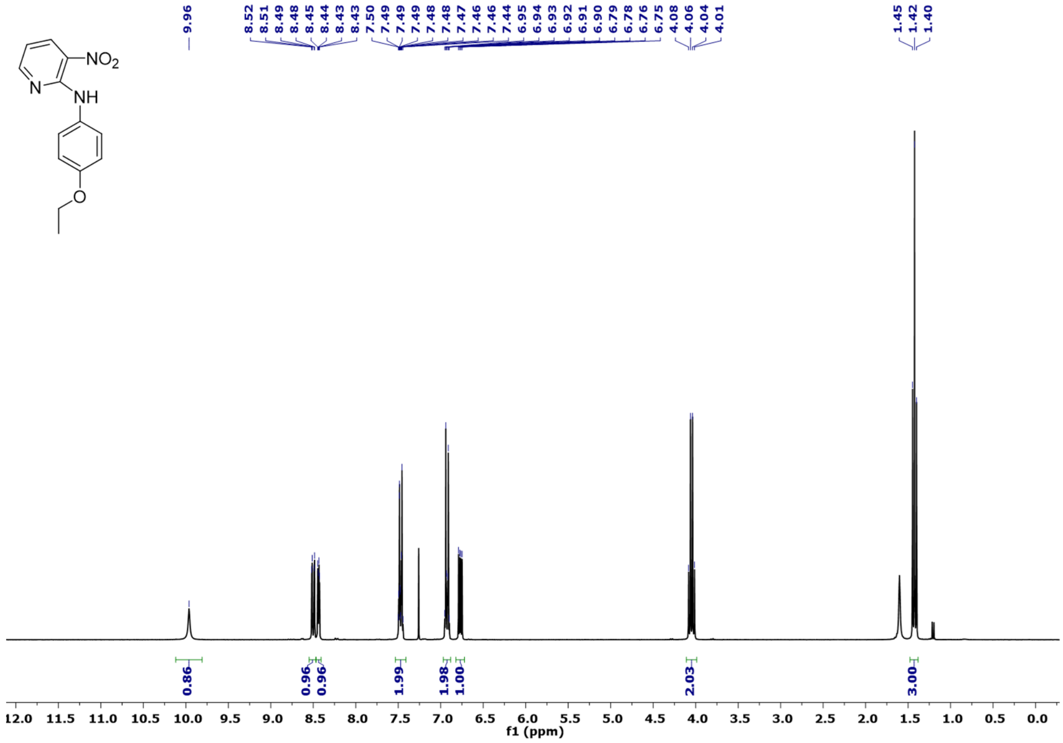

Figure S6: ^{1}H NMR spectrum (300 MHz) of **1d** in $CDCl_3$ at room temperature.

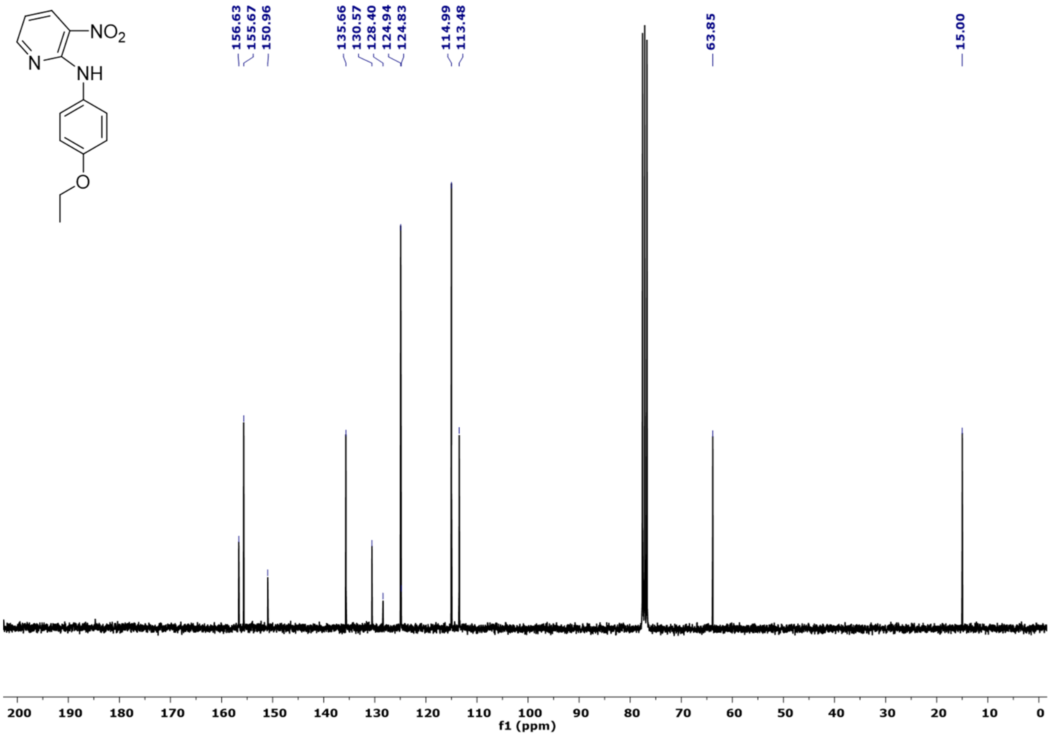

Figure S7: ^{13}C NMR spectrum (75 MHz) of **1d** in $CDCl_3$ at room temperature.

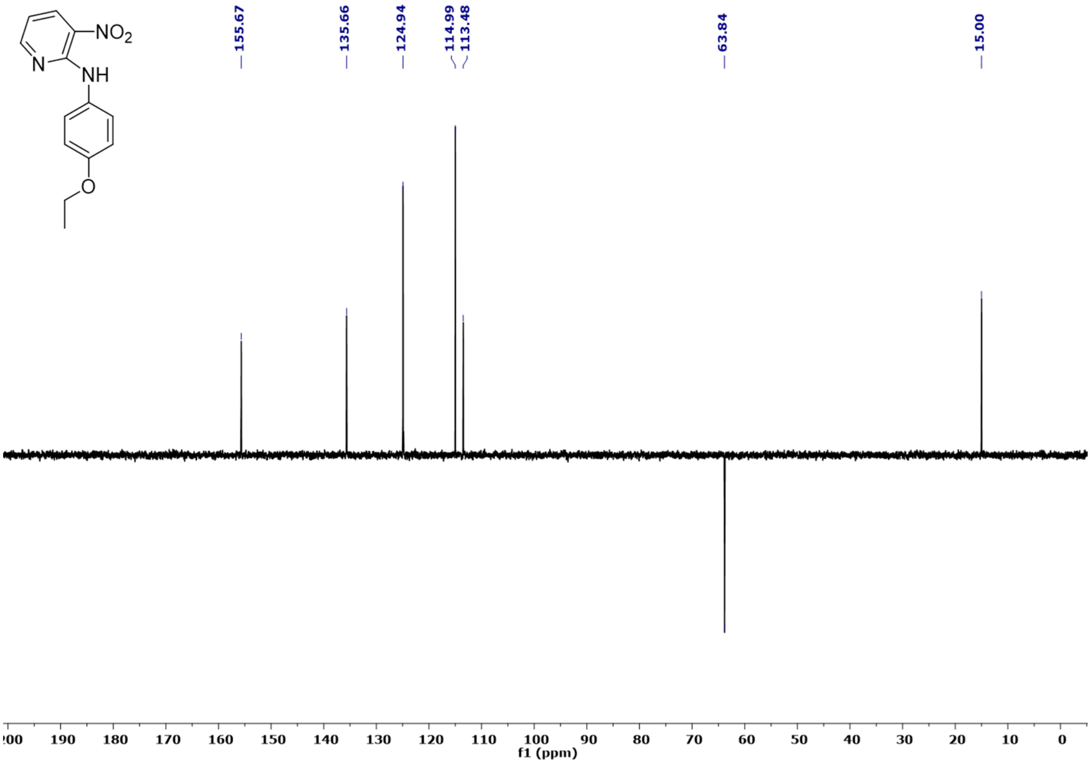

Figure S8: ^{13}C NMR (DEPT-135) spectrum (75 MHz) of **1d** in $CDCl_3$ at room temperature.

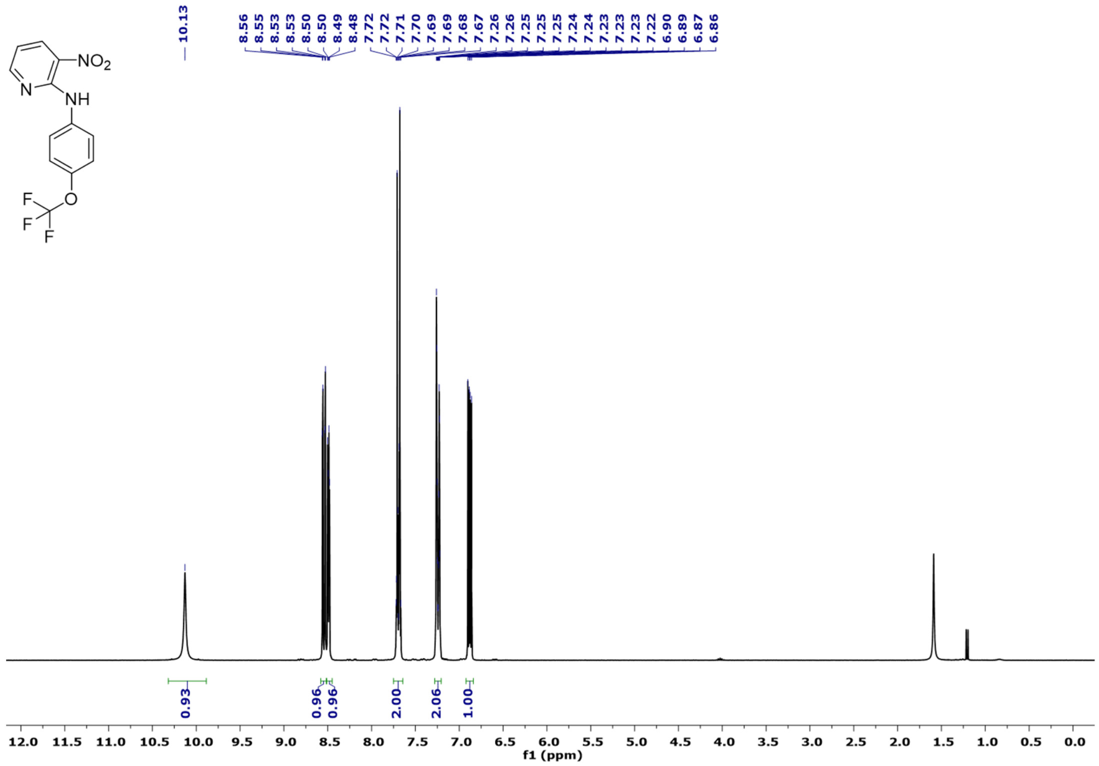

Figure S9: ^{1}H NMR spectrum (300 MHz) of **1e** in $CDCl_3$ at room temperature.

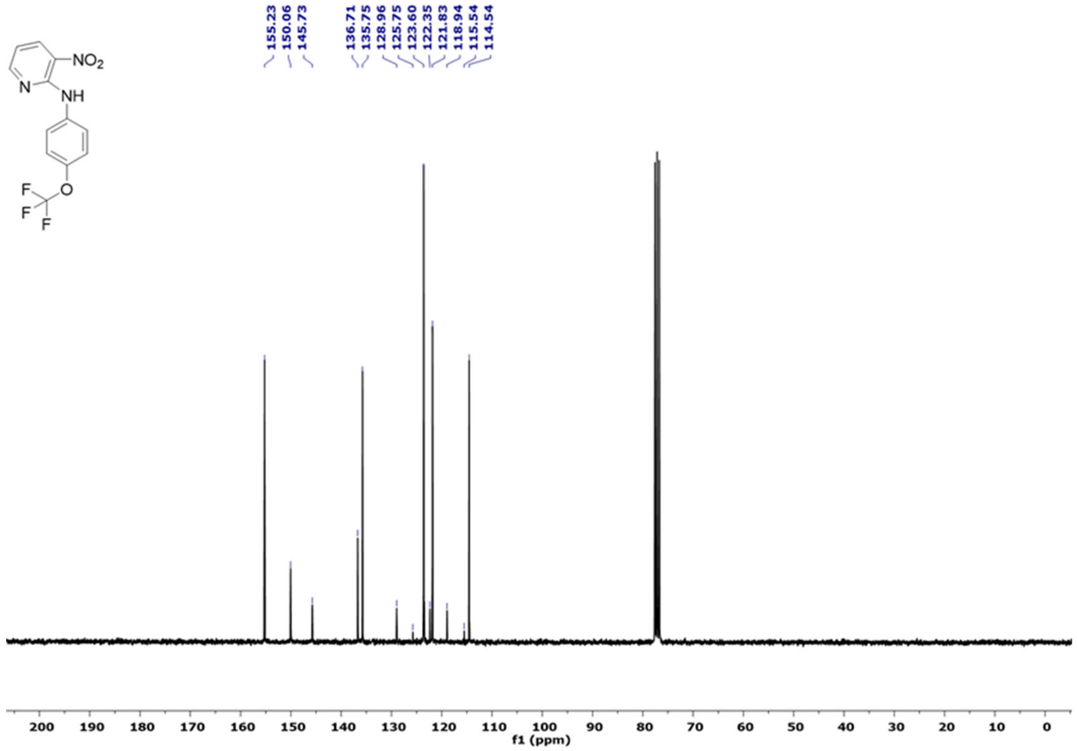

Figure S10: ^{13}C NMR spectrum (75 MHz) of **1e** in $CDCl_3$ at room temperature.

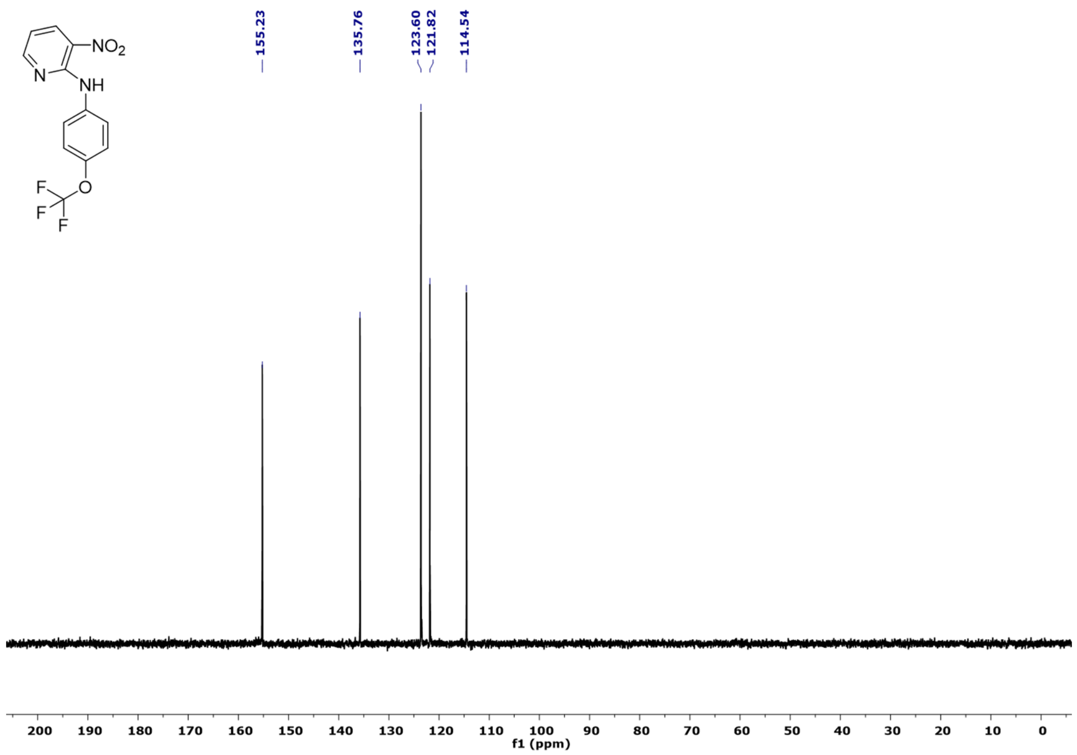

Figure S11: ^{13}C NMR (DEPT-135) spectrum (75 MHz) of **1e** in $CDCl_3$ at room temperature.

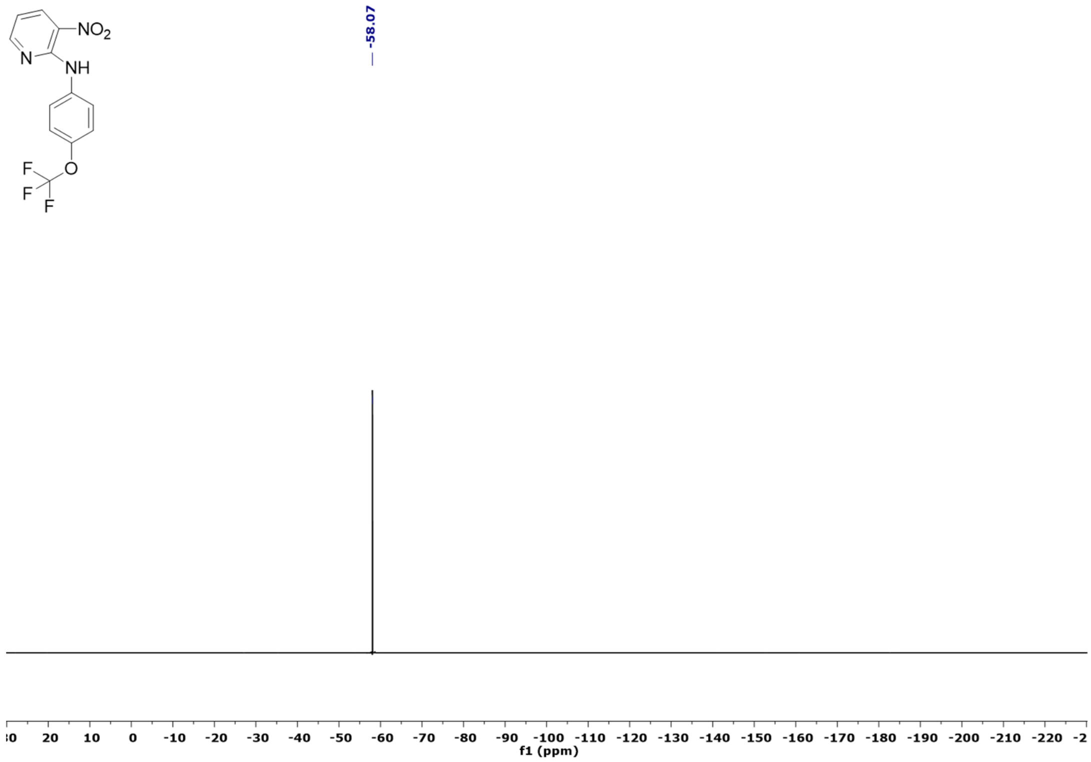

Figure S12: ^{19}F NMR spectrum (282 MHz) of **1e** in $CDCl_3$ at room temperature.

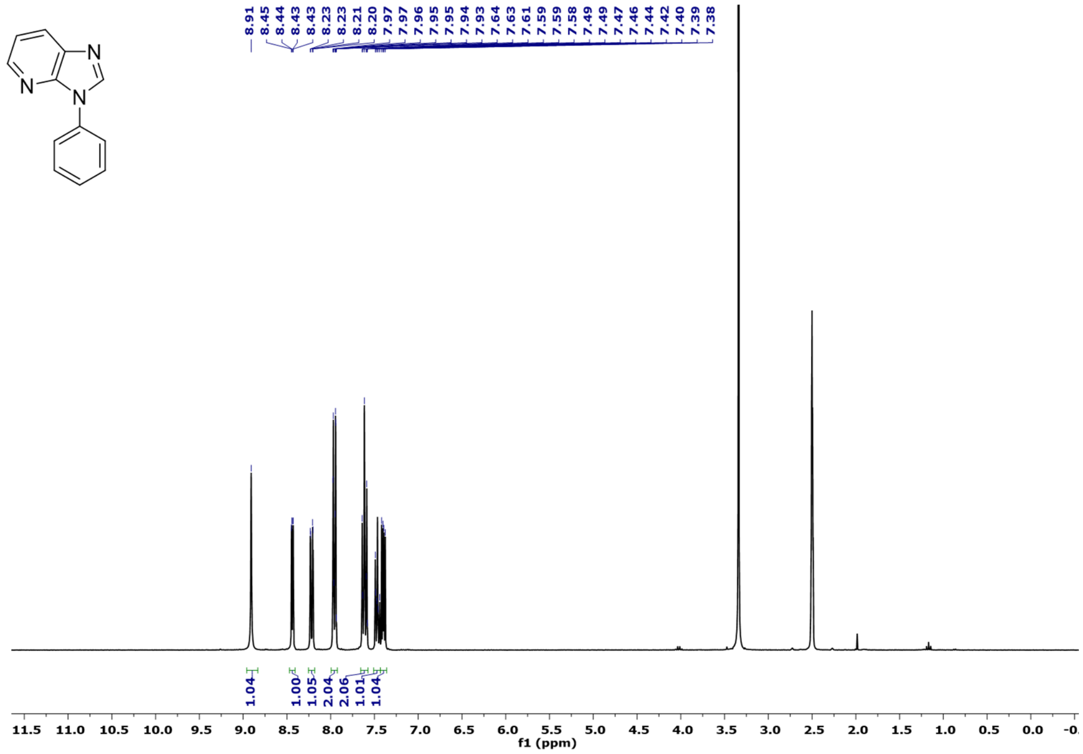

Figure S13: ^{1}H NMR spectrum (300 MHz) of **2a** in DMSO-d_6 at room temperature.

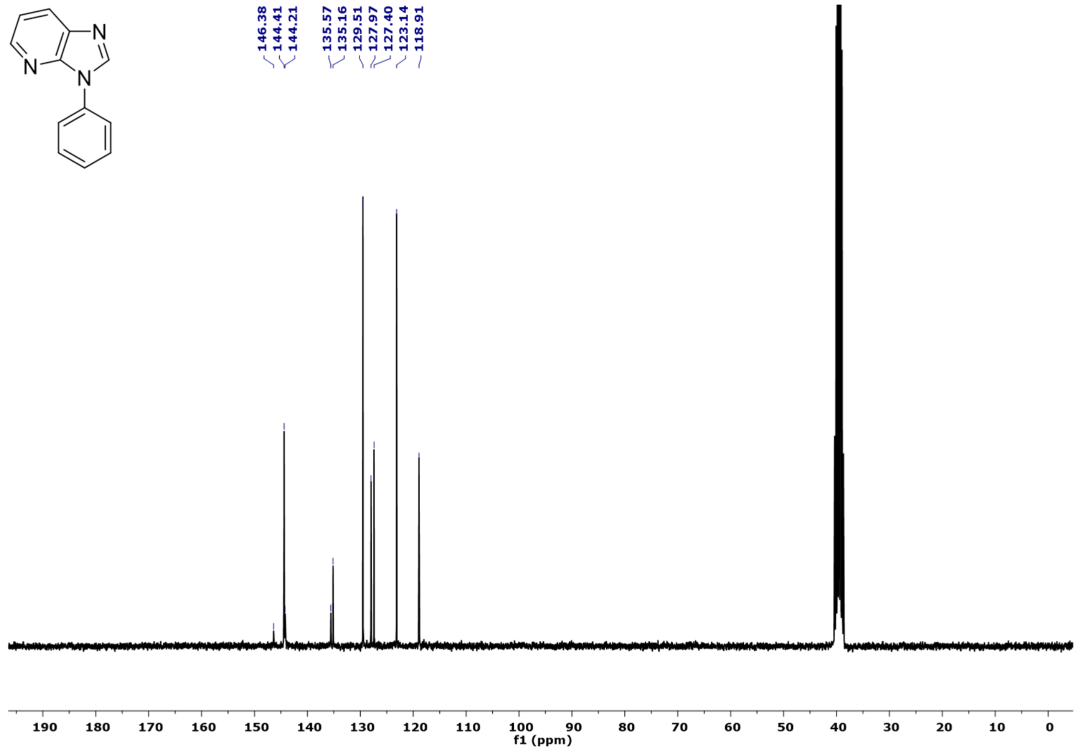

Figure S14: ^{13}C NMR spectrum (75 MHz) of **2a** in DMSO-d_6 at room temperature.

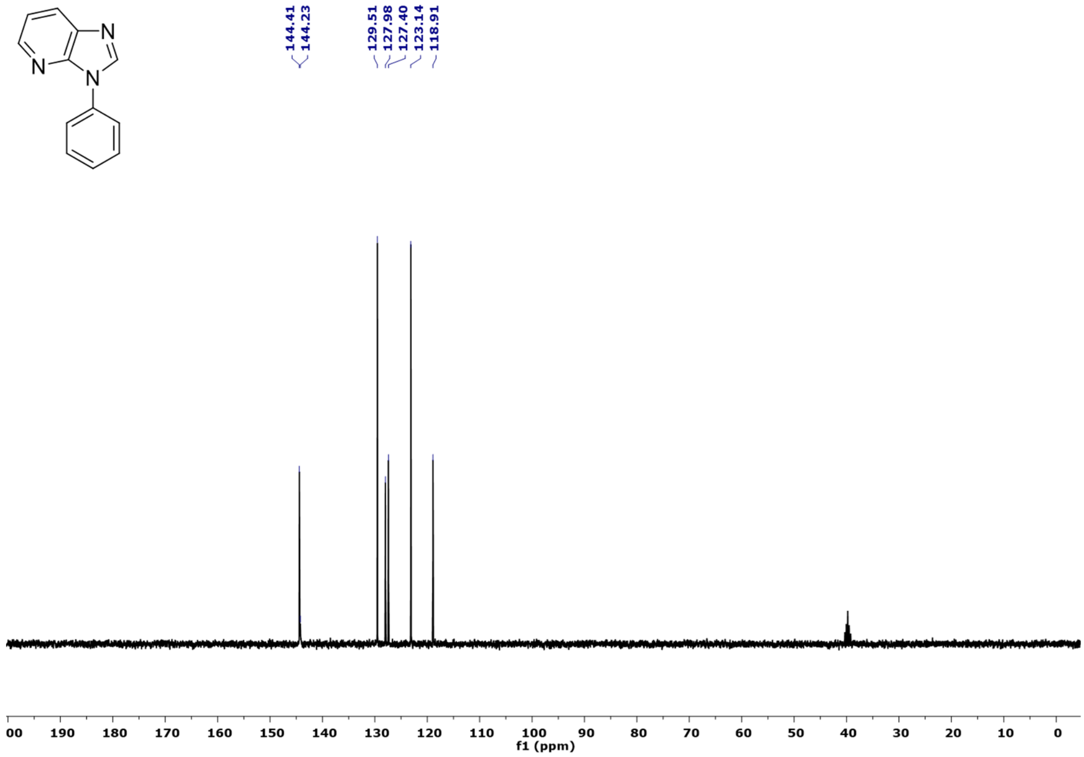

Figure S15: ^{13}C NMR (DEPT-135) spectrum (75 MHz) of **2a** in DMSO-d_6 at room temperature.

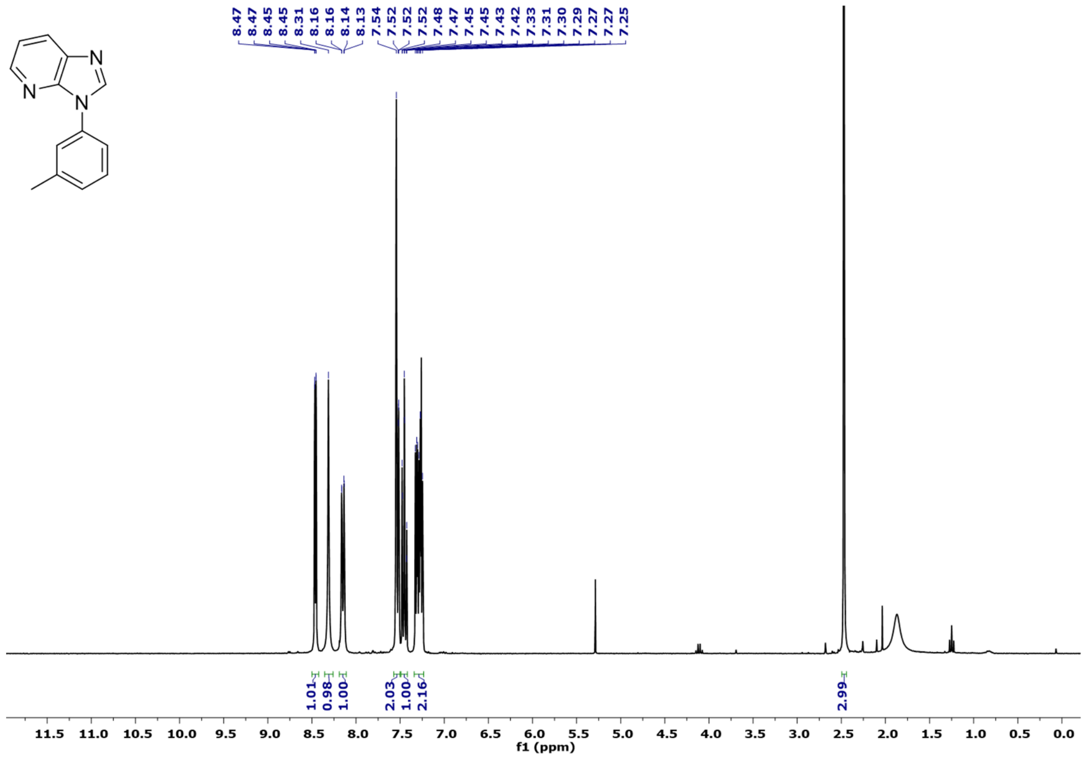

Figure S16: ^{1}H NMR spectrum (300 MHz) of **2b** in $CDCl_3$ at room temperature.

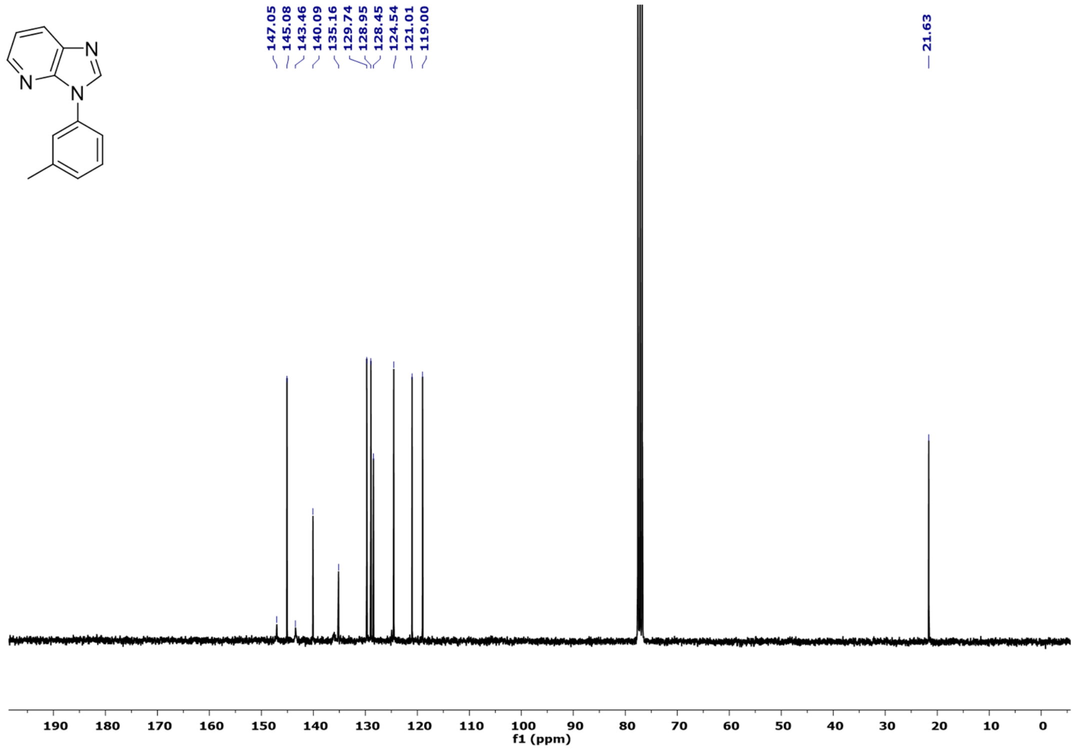

Figure S17: ^{13}C NMR spectrum (75 MHz) of **2b** in $CDCl_3$ at room temperature.

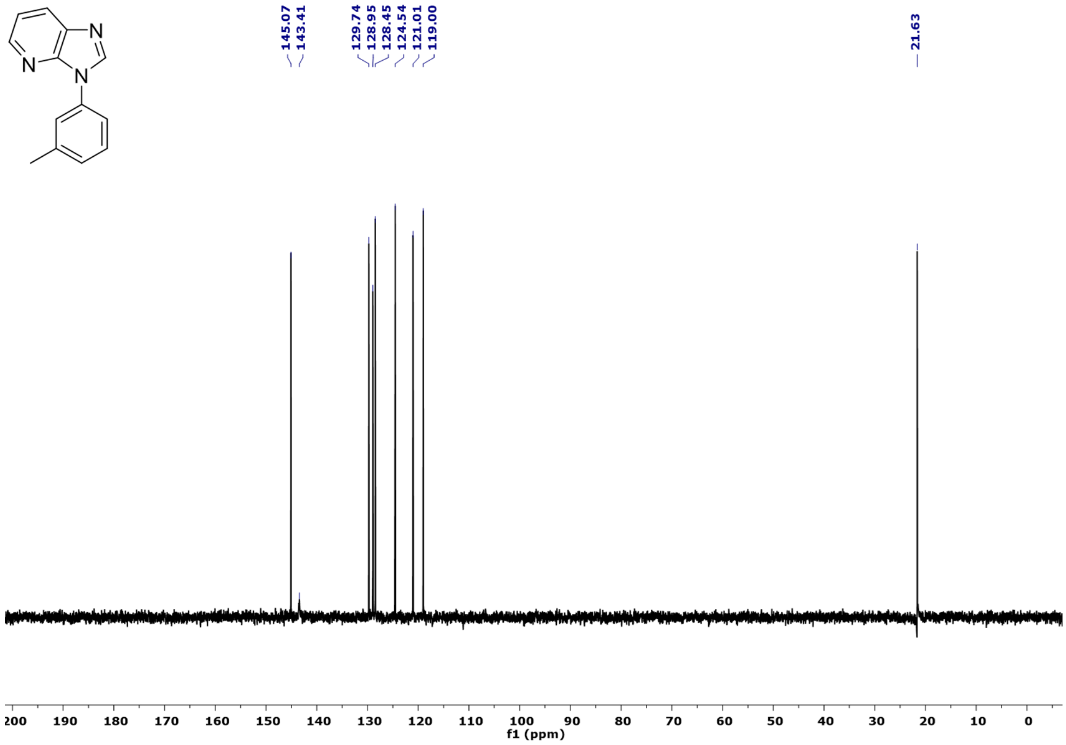

Figure S18: ^{13}C NMR (DEPT-135) spectrum (75 MHz) of **2b** in $CDCl_3$ at room temperature.

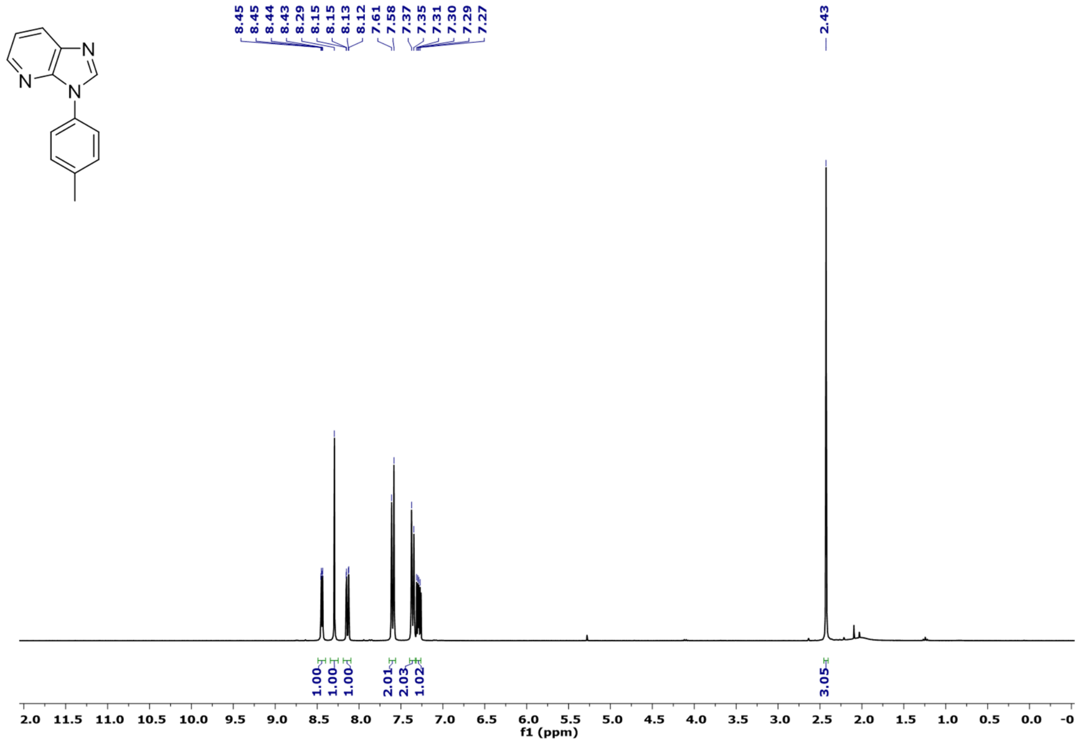

Figure S19: ^{1}H NMR spectrum (300 MHz) of **2c** in $CDCl_3$ at room temperature.

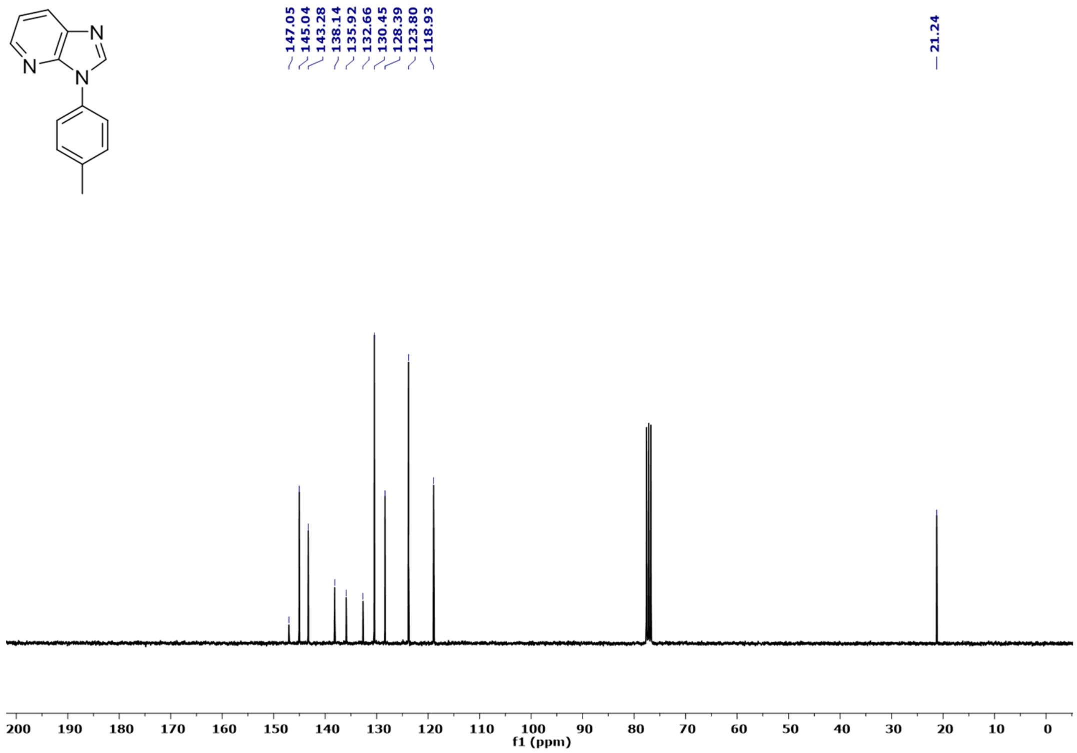

Figure S20: ^{13}C NMR spectrum (75 MHz) of **2c** in $CDCl_3$ at room temperature.

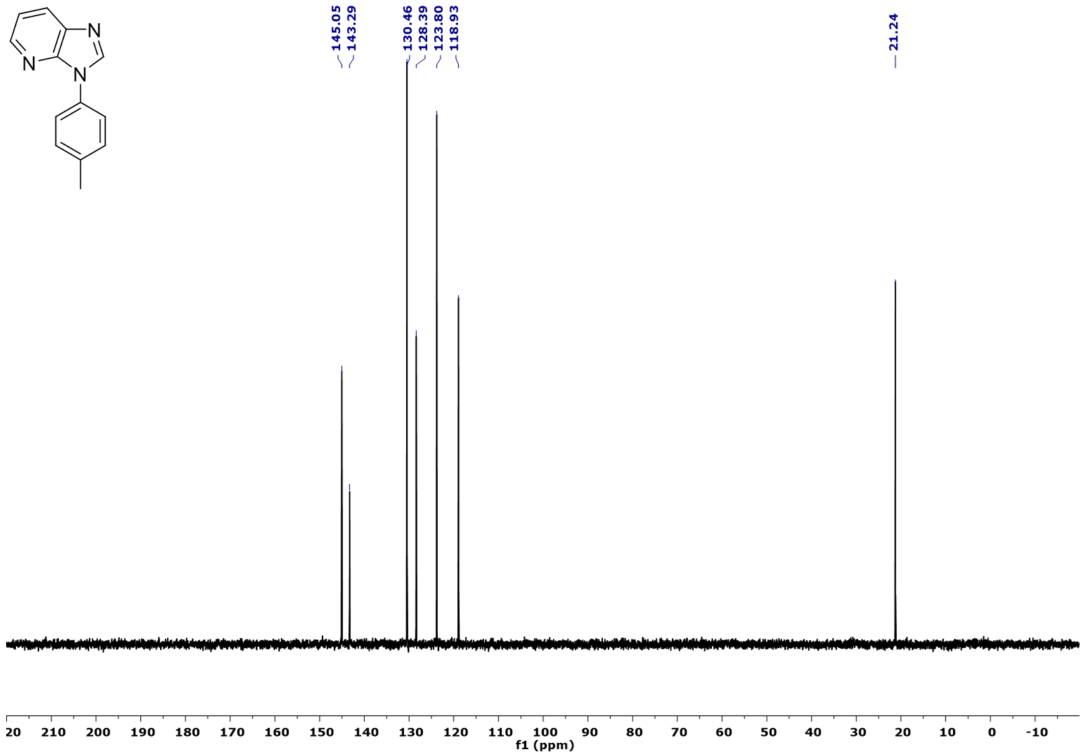

Figure S21: ^{13}C NMR (DEPT-135) spectrum (75 MHz) of **2c** in $CDCl_3$ at room temperature.

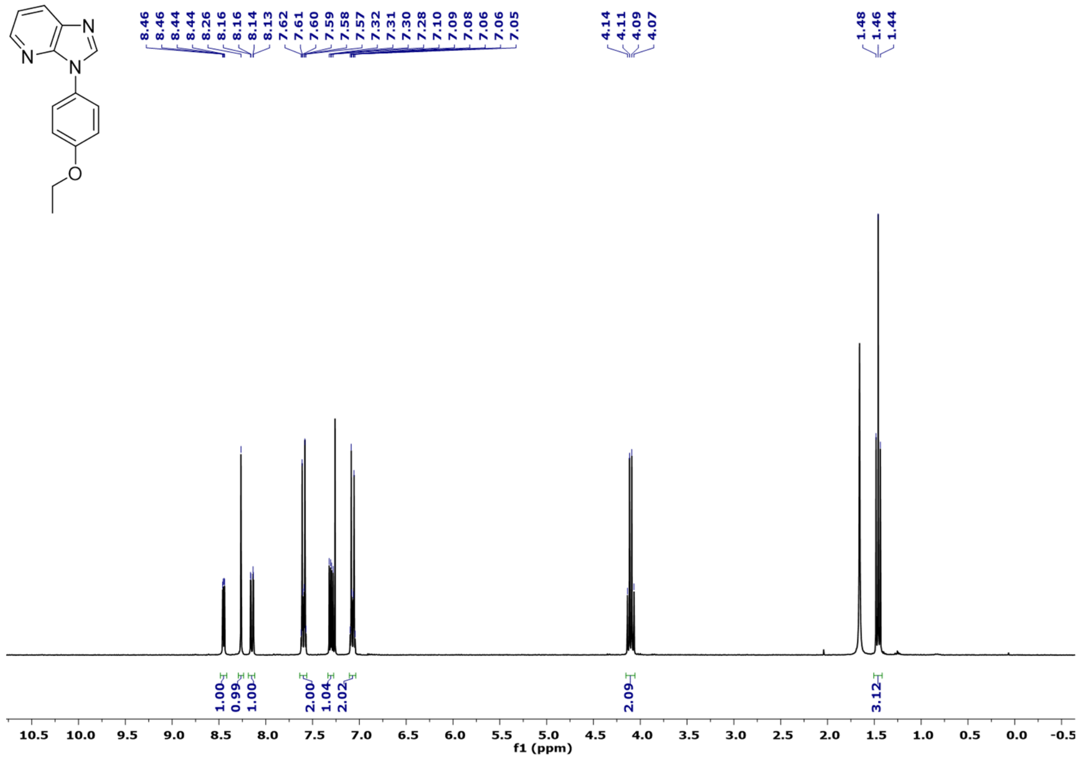

Figure S22: ^{1}H NMR spectrum (300 MHz) of **2d** in $CDCl_3$ at room temperature.

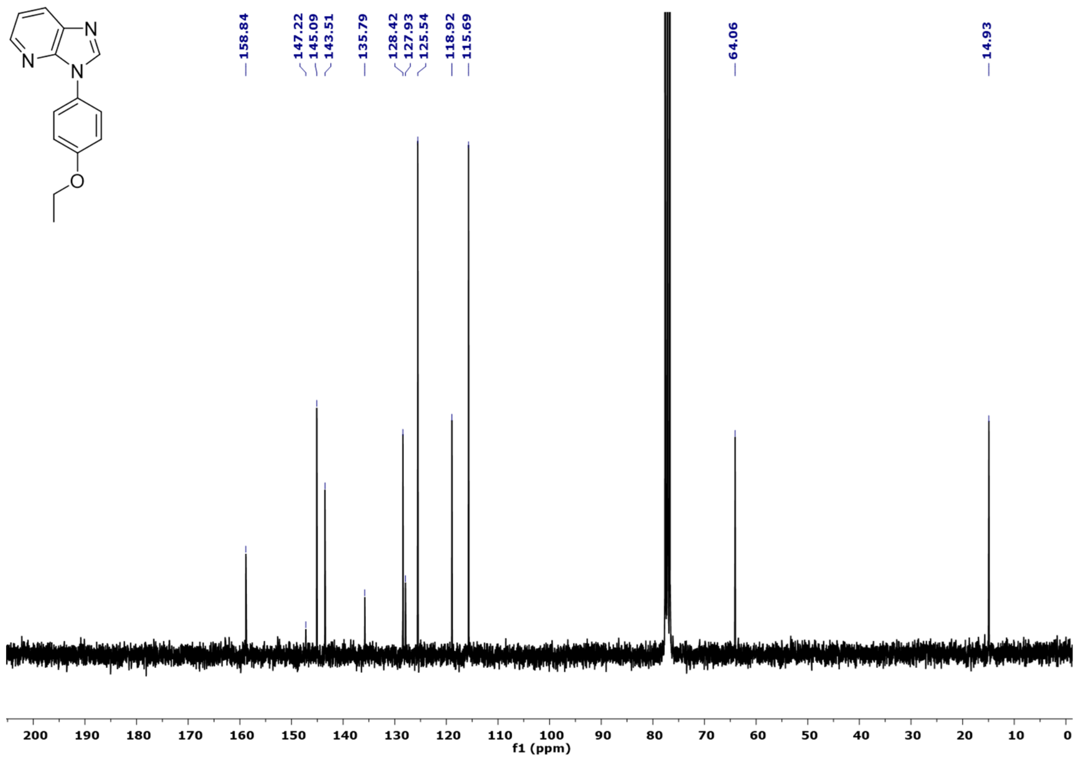

Figure S23: ^{13}C NMR spectrum (75 MHz) of **2d** in $CDCl_3$ at room temperature.

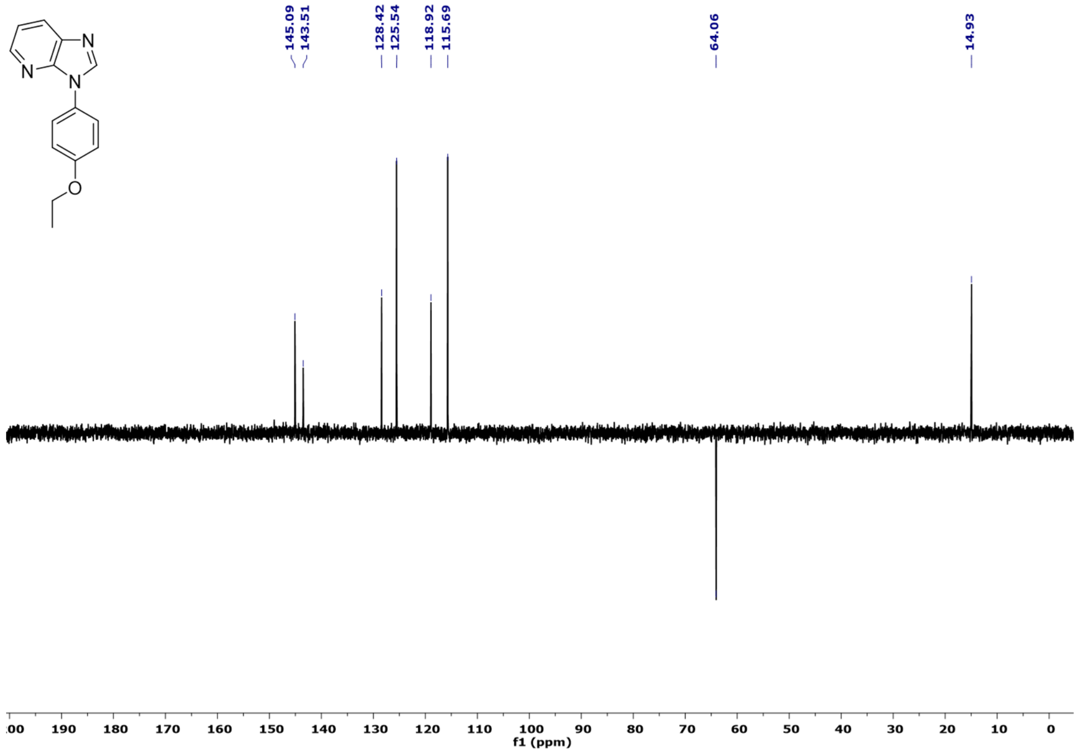

Figure S24: ^{13}C NMR (DEPT-135) spectrum (75 MHz) of **2d** in $CDCl_3$ at room temperature.

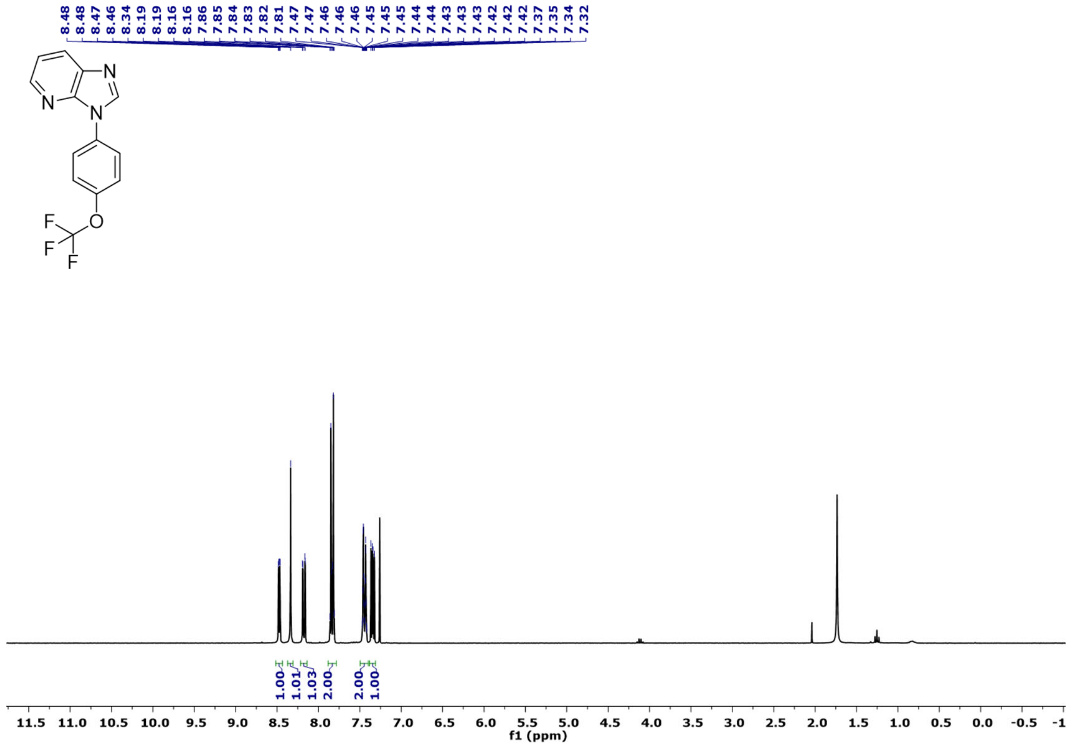

Figure S25: ^{1}H NMR spectrum (300 MHz) of **2e** in $CDCl_3$ at room temperature.

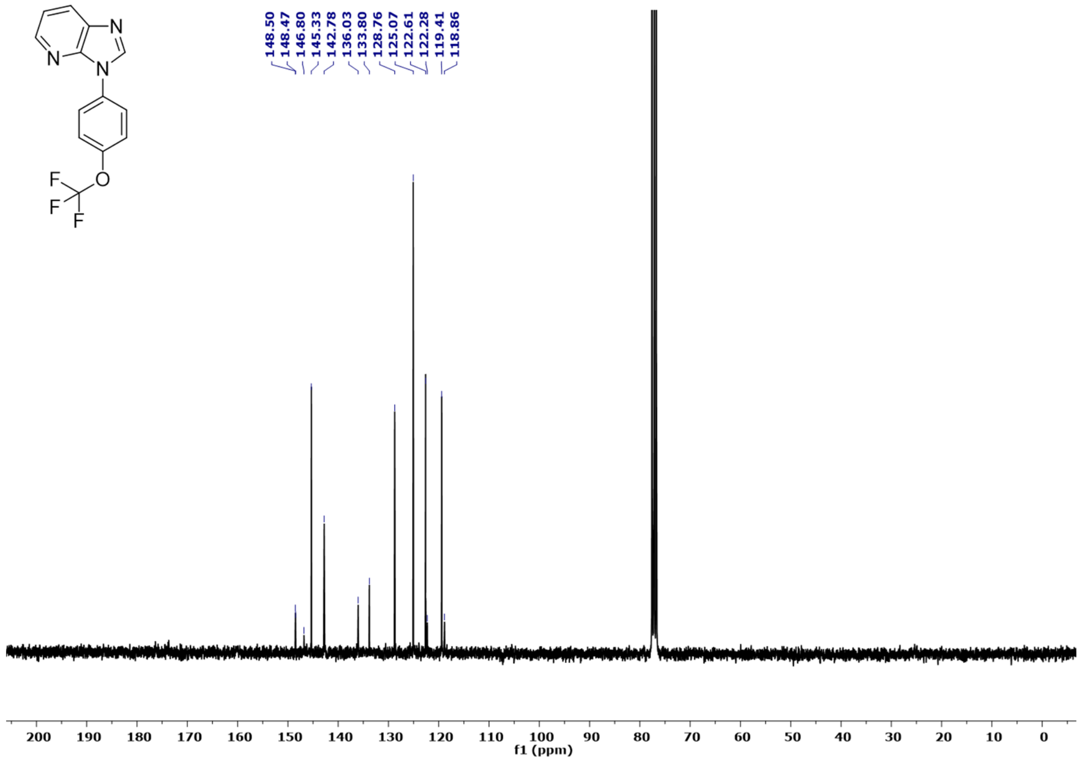

Figure S26: ^{13}C NMR spectrum (75 MHz) of **2e** in $CDCl_3$ at room temperature.

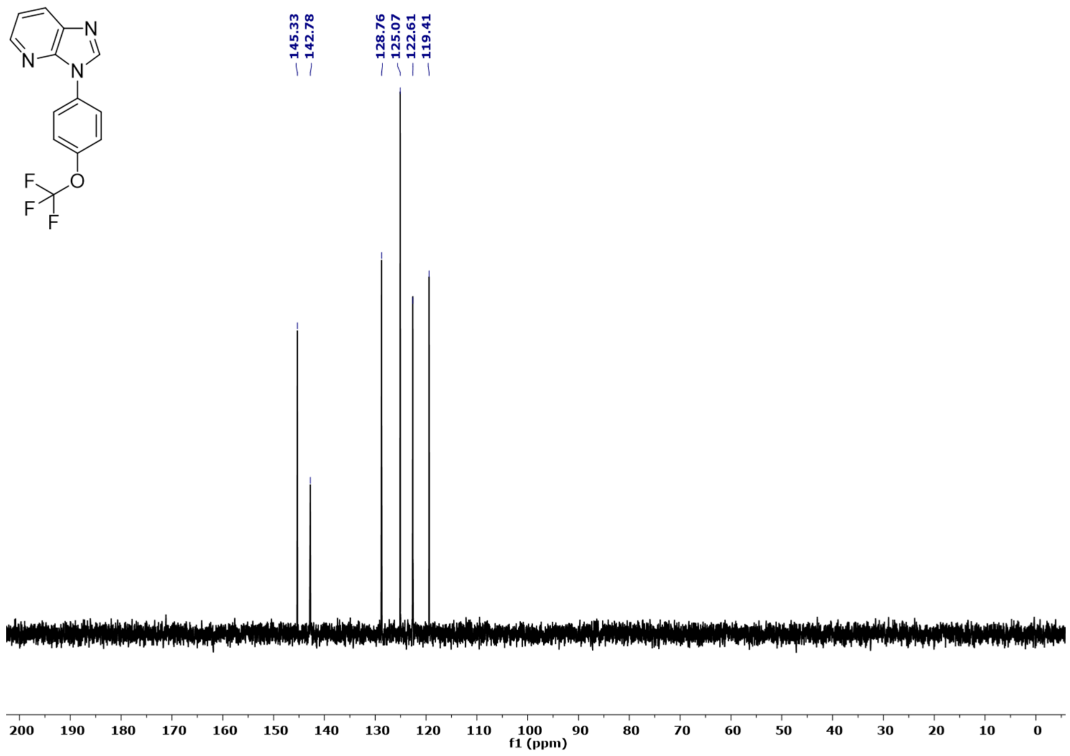

Figure S27: ^{13}C NMR (DEPT-135) spectrum (75 MHz) of **2e** in $CDCl_3$ at room temperature.

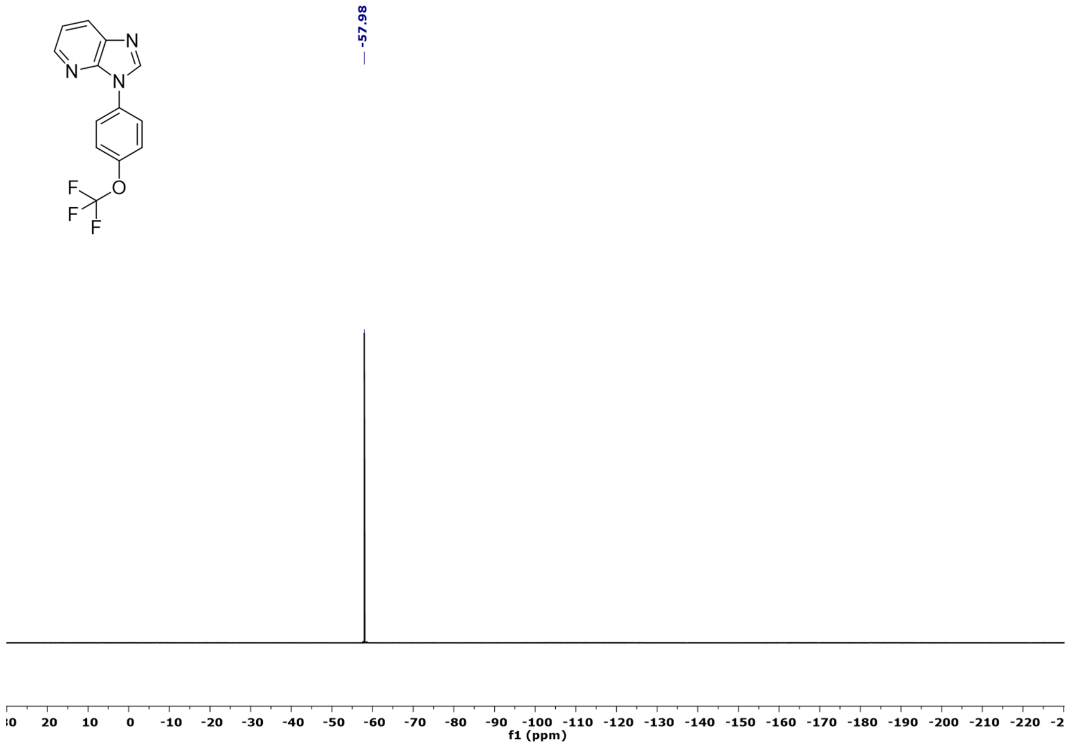

Figure S28: ^{19}F NMR spectrum (282 MHz) of **2e** in $CDCl_3$ at room temperature.

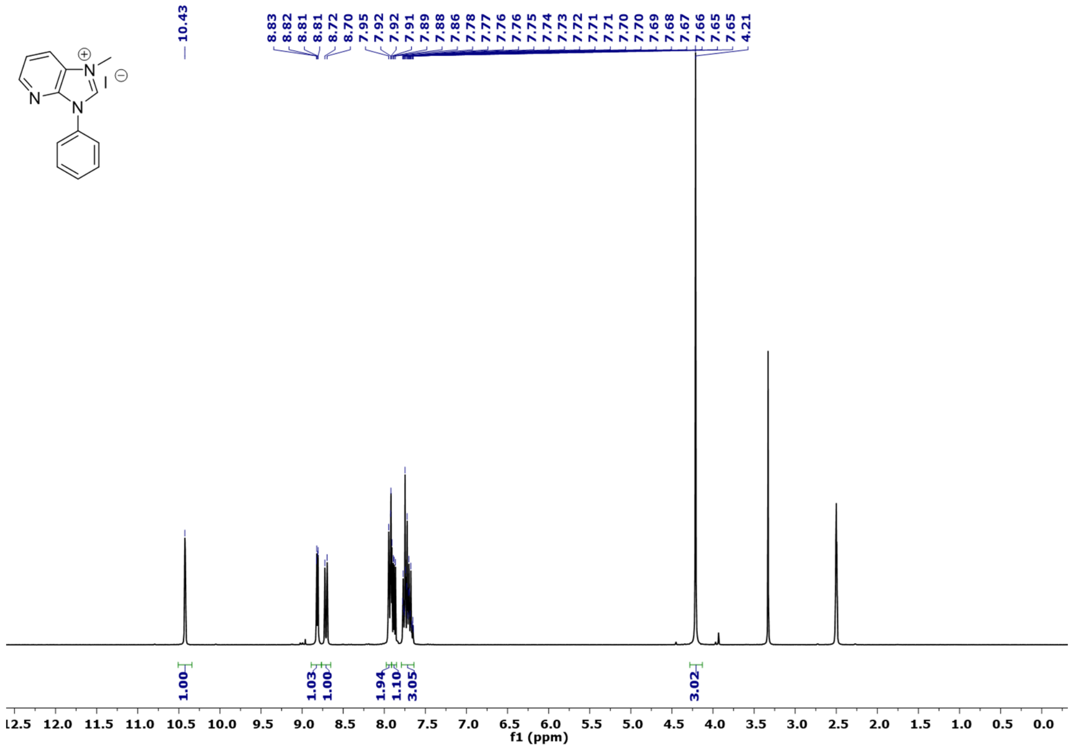

Figure S29: ^{1}H NMR spectrum (300 MHz) of **3a** in DMSO-d$_6$ at room temperature.

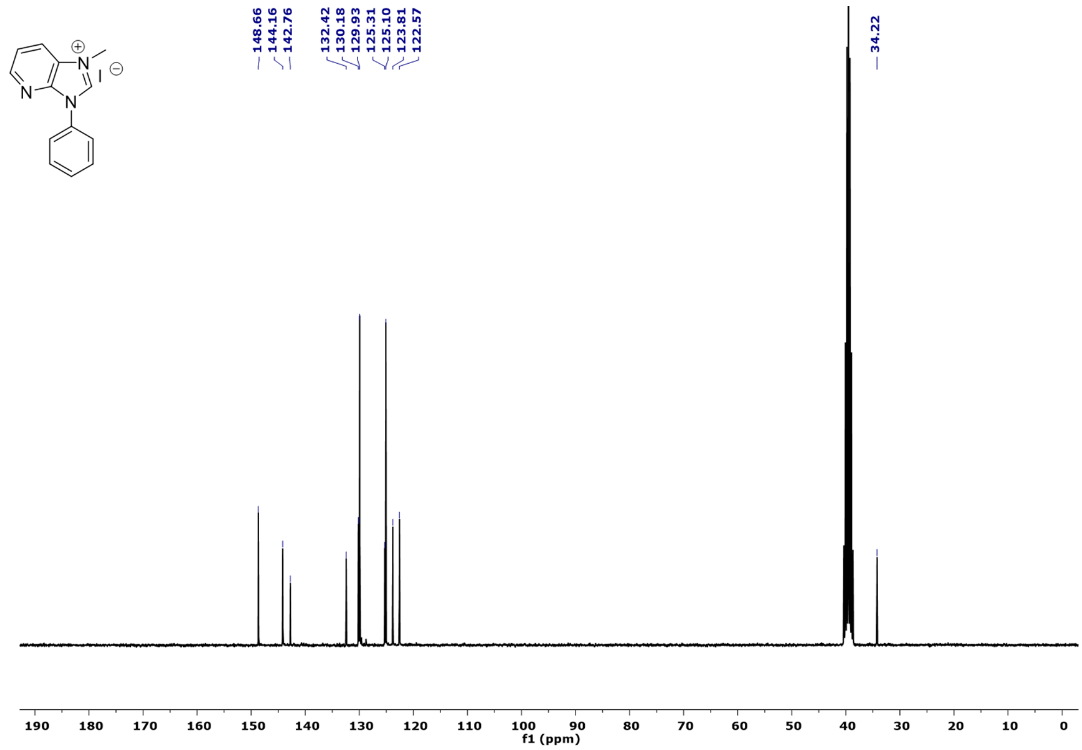

Figure S30: ^{13}C NMR spectrum (75 MHz) of **3a** in DMSO-d$_6$ at room temperature.

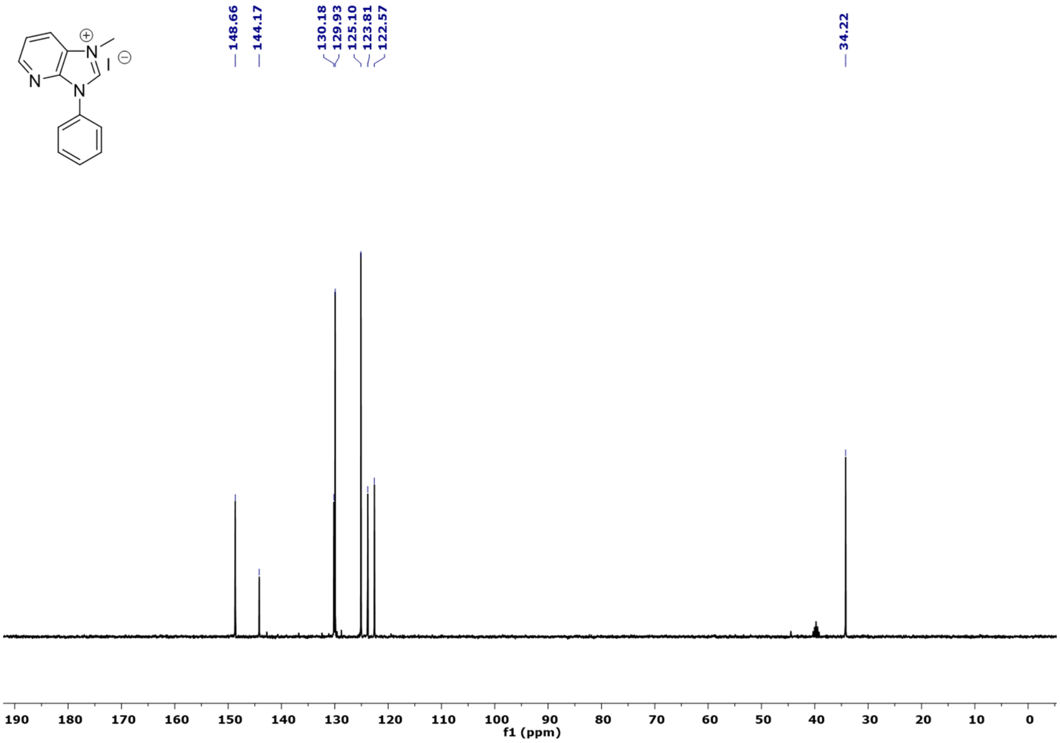

Figure S31: ^{13}C NMR (DEPT-135) spectrum (75 MHz) of **3a** in DMSO-d_6 at room temperature.

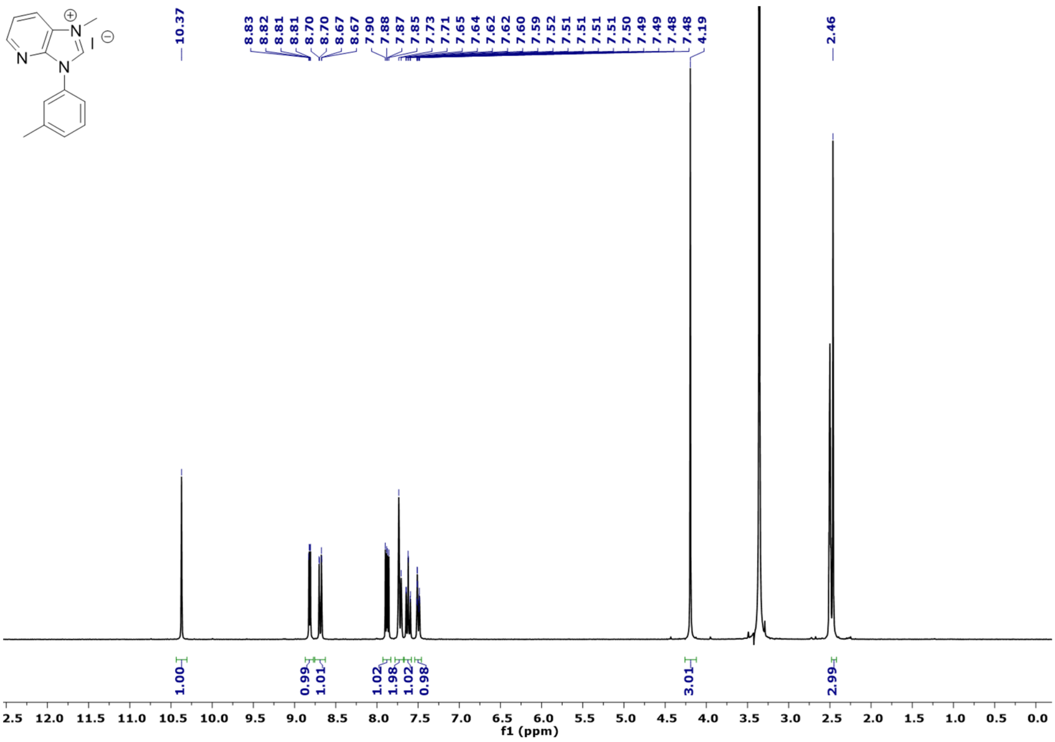

Figure S32: ^1H NMR spectrum (75 MHz) of **3b** in DMSO-d_6 at room temperature.

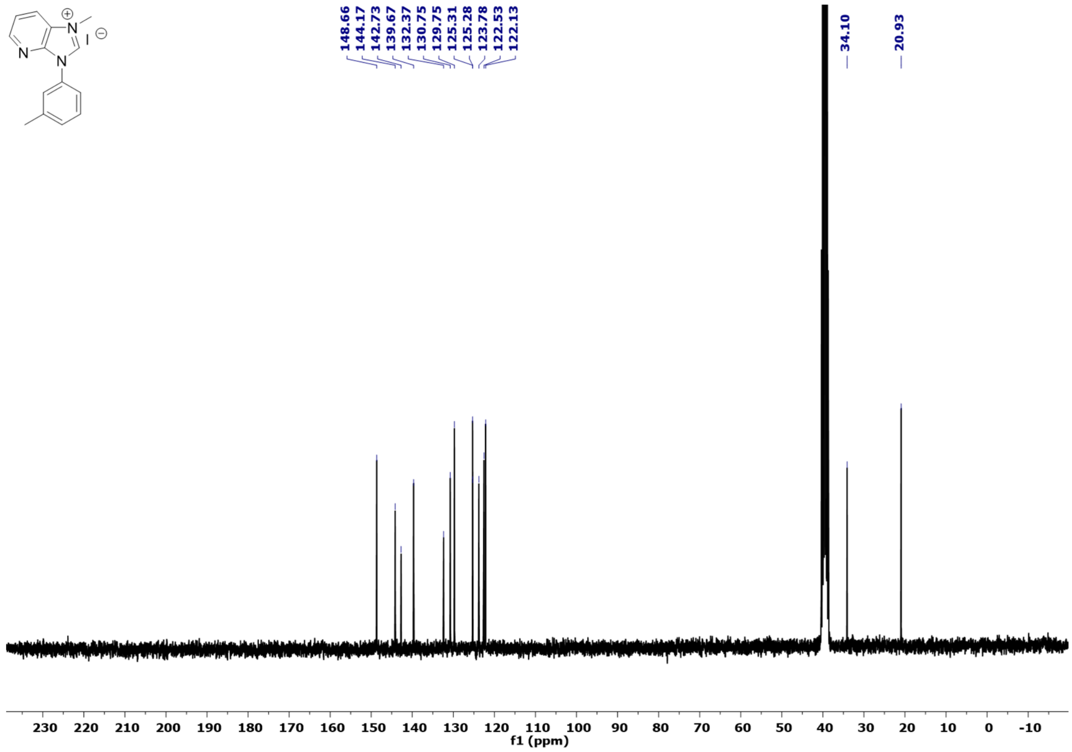

Figure S33: ^{13}C NMR spectrum (75 MHz) of **3b** in DMSO-d_6 at room temperature.

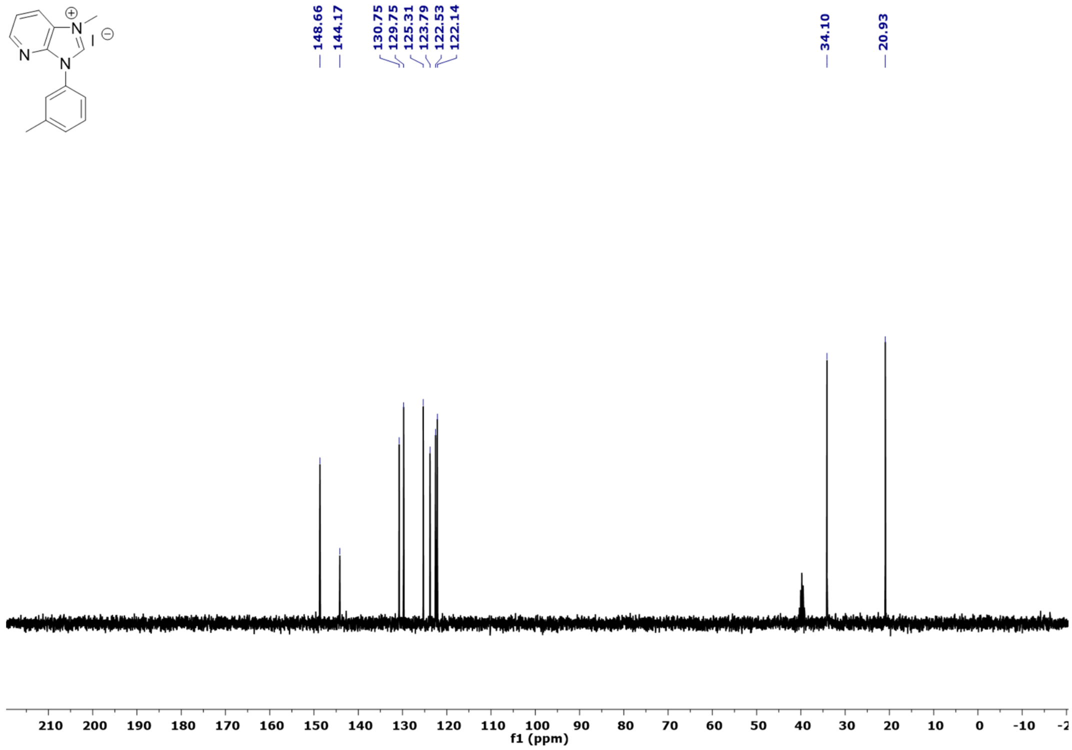

Figure S34: ^{13}C NMR (DEPT-135) spectrum (75 MHz) of **3b** in DMSO-d_6 at room temperature.

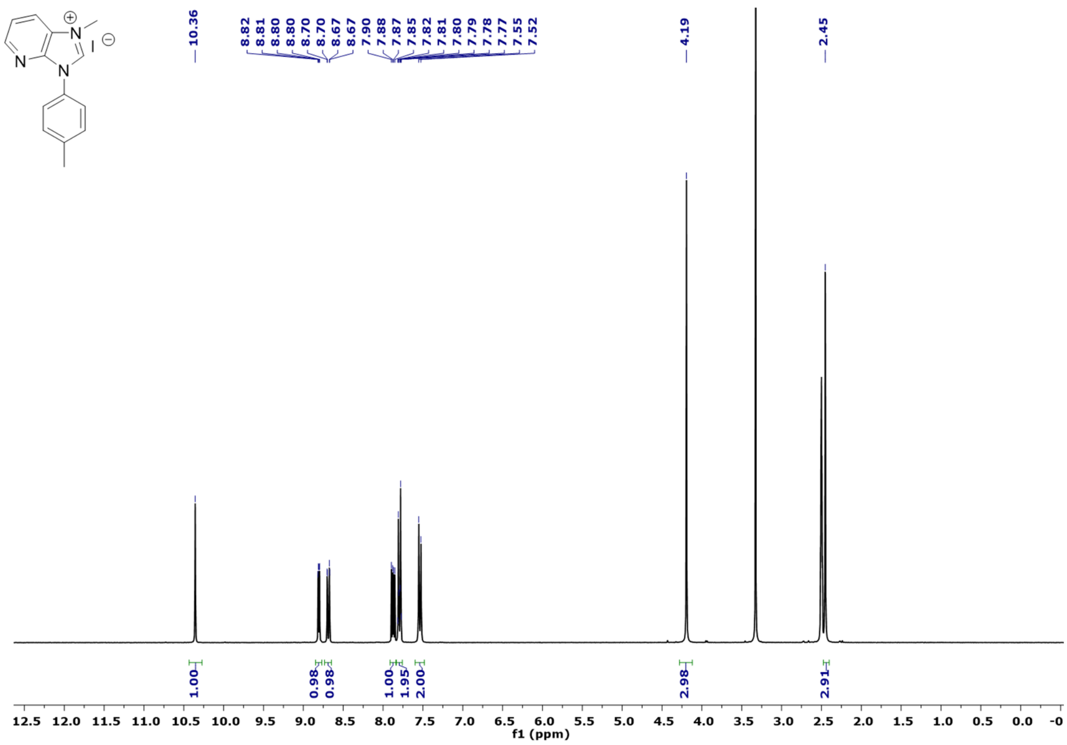

Figure S35: ^{1}H NMR spectrum (300 MHz) of **3c** in DMSO-d_6 at room temperature.

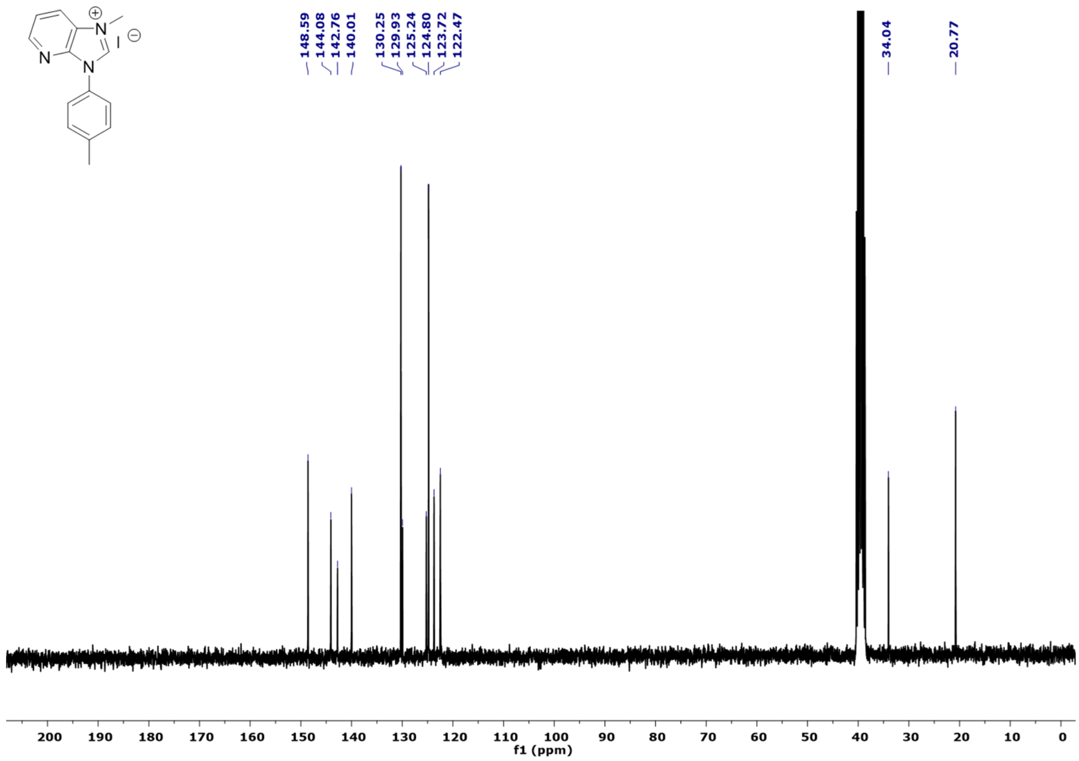

Figure S36: ^{13}C NMR spectrum (75 MHz) of **3c** in DMSO-d_6 at room temperature.

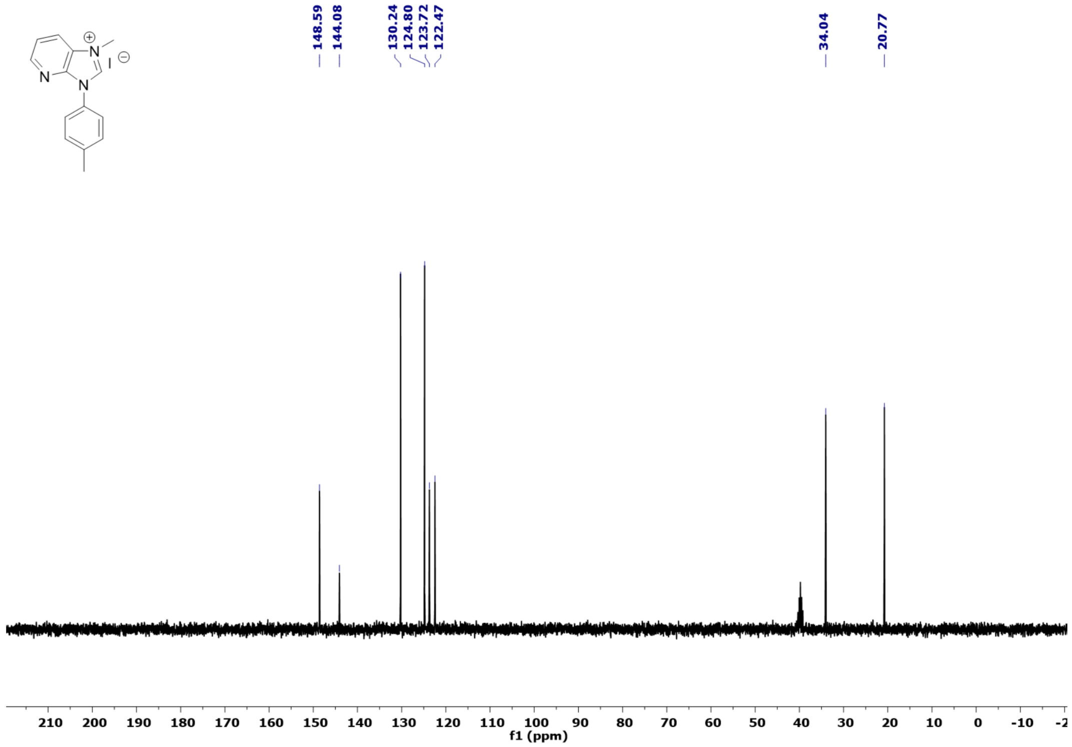

Figure S37: ^{13}C NMR (DEPT-135) spectrum (75 MHz) of **3c** in DMSO-d$_6$ at room temperature.

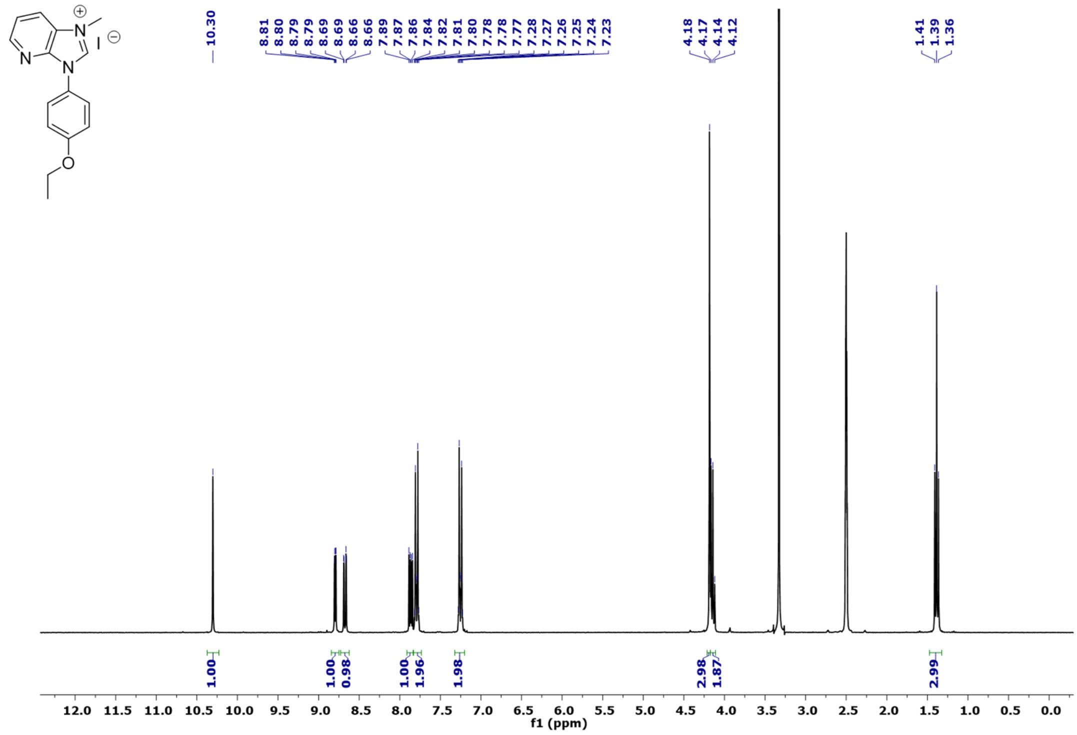

Figure S38: ^{1}H NMR spectrum (300 MHz) of **3d** in DMSO-d$_6$ at room temperature.

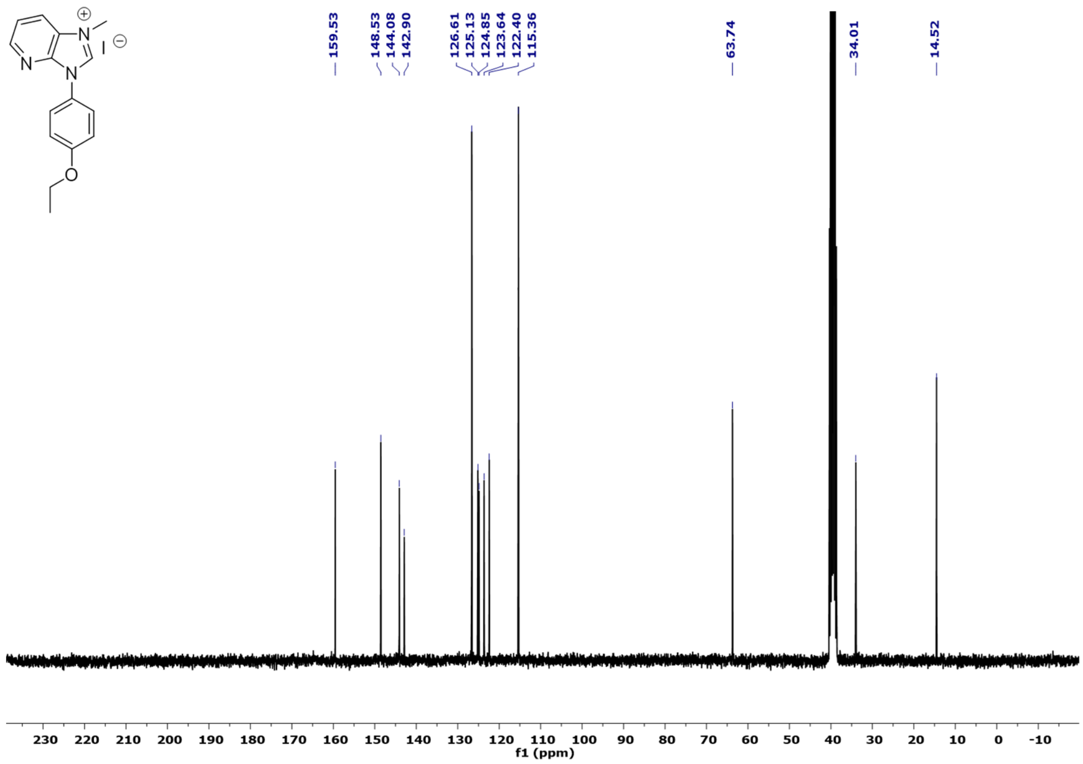

Figure S39: ^{13}C NMR spectrum (75 MHz) of **3d** in DMSO-d$_6$ at room temperature.

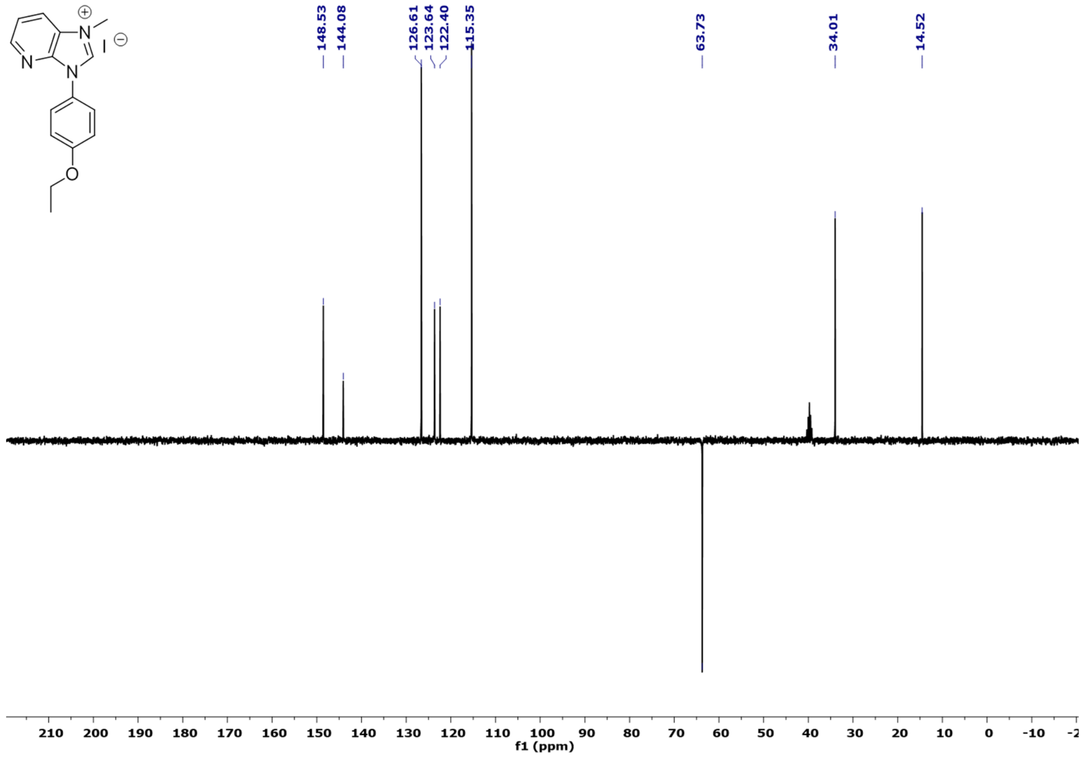

Figure S40: ^{13}C NMR (DEPT-135) spectrum (75 MHz) of **3d** in DMSO-d$_6$ at room temperature.

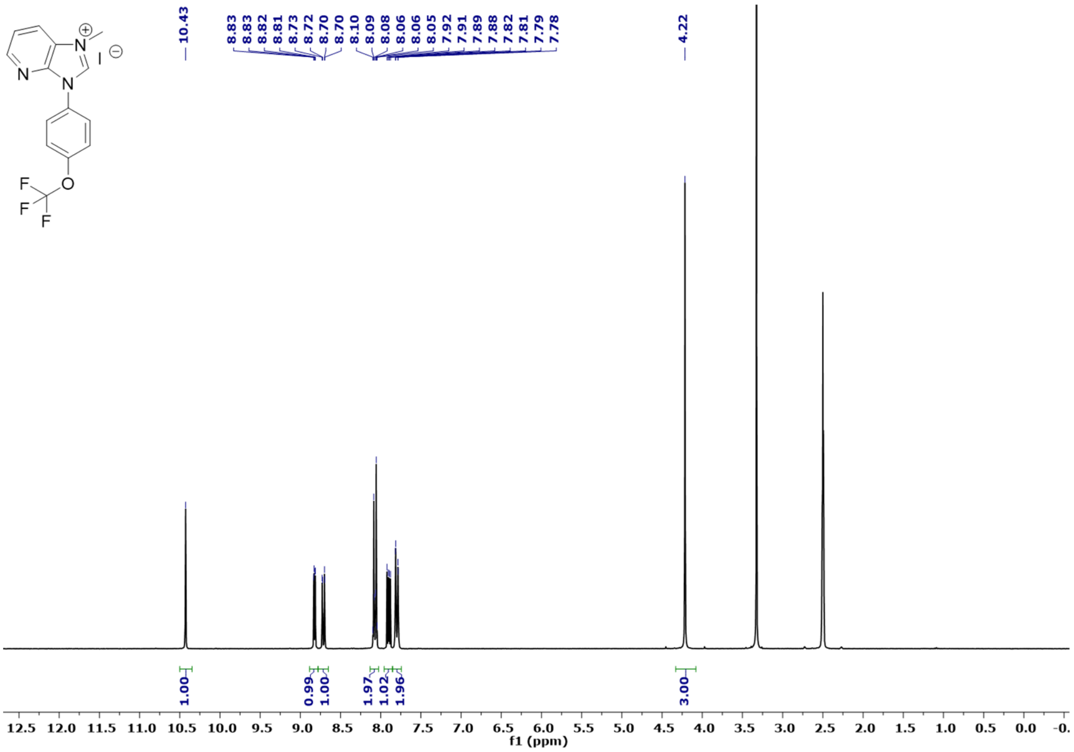

Figure S41: ^{1}H NMR spectrum (300 MHz) of **3e** in DMSO-d_6 at room temperature.

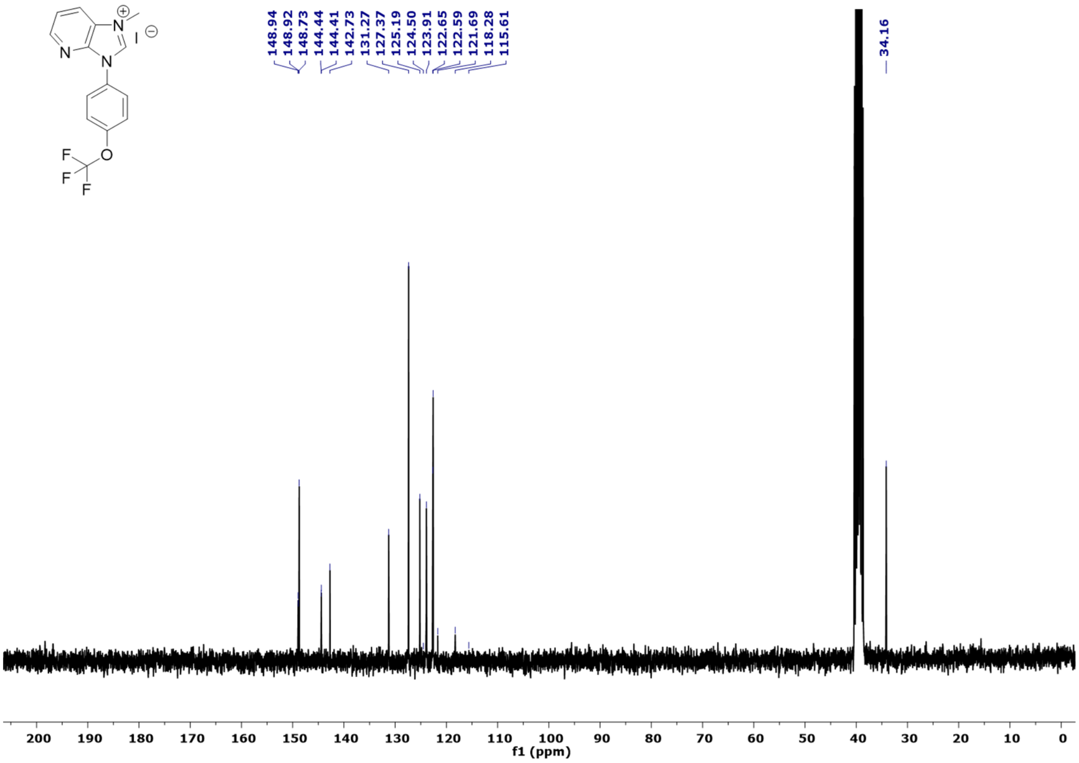

Figure S42: ^{13}C NMR spectrum (75 MHz) of **3e** in DMSO-d_6 at room temperature.

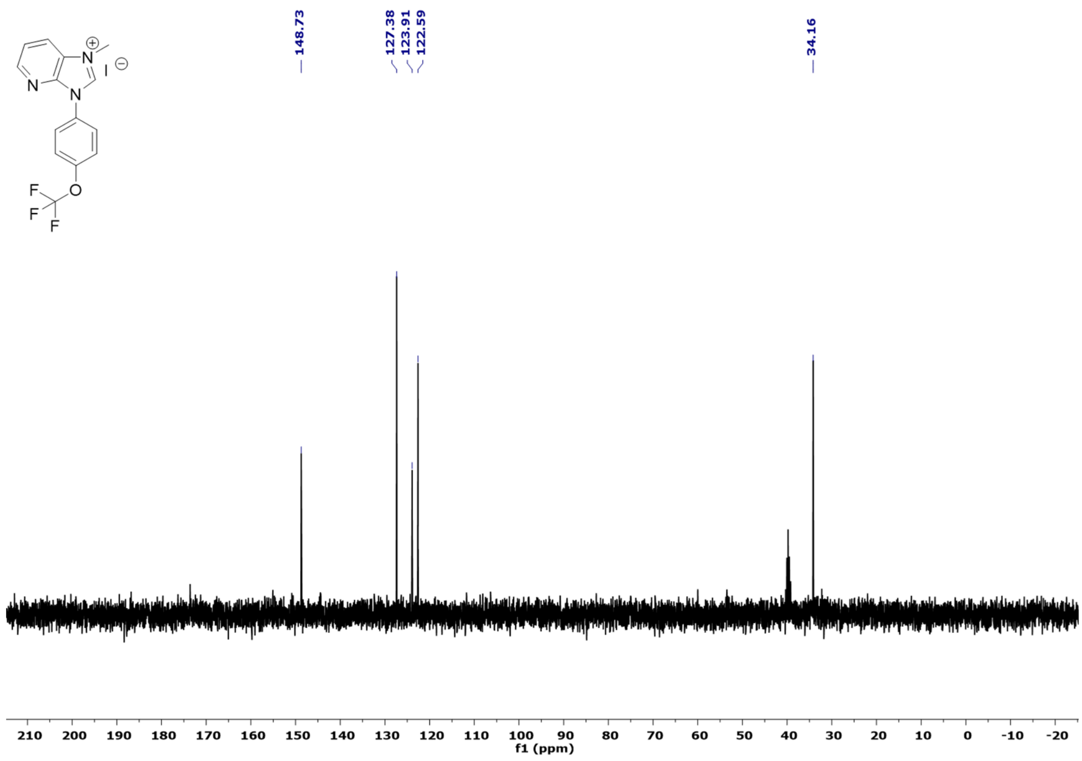

Figure S43: ^{13}C NMR (DEPT-135) spectrum (75 MHz) of **3e** in DMSO-d$_6$ at room temperature.

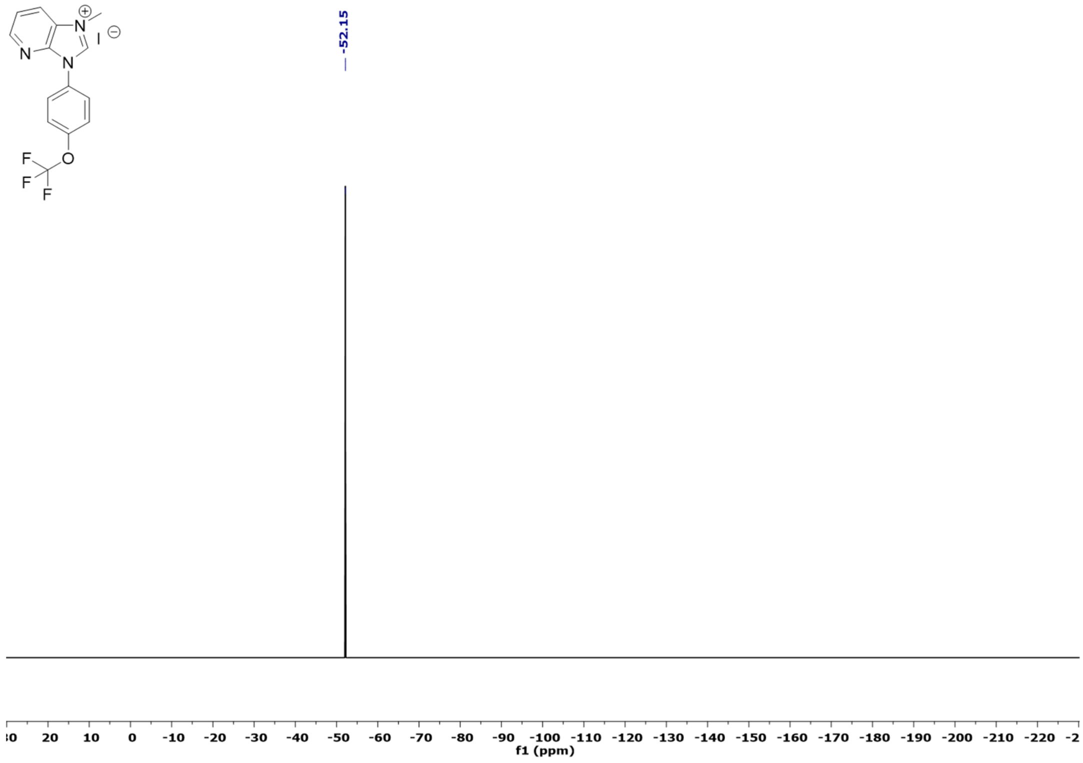

Figure S44: ^{19}F NMR spectrum (282 MHz) of **3e** in DMSO-d$_6$ at room temperature.

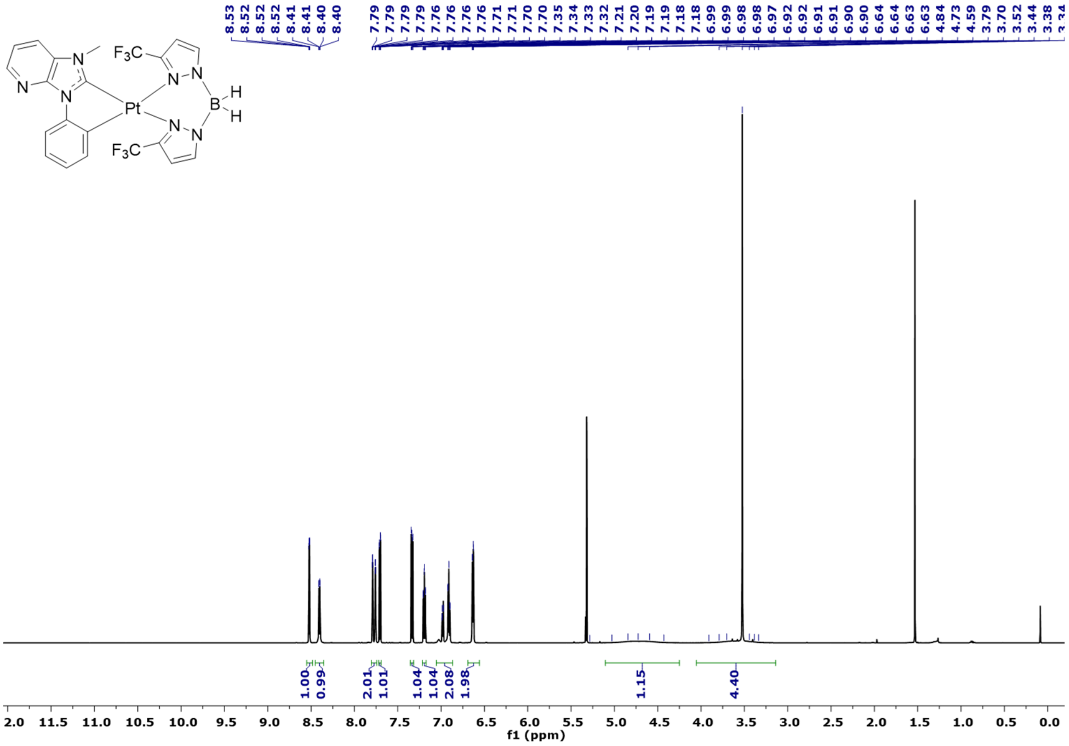

Figure S45: ^{1}H NMR spectrum (600 MHz) of **5a** in DCM-d_2 at room temperature.

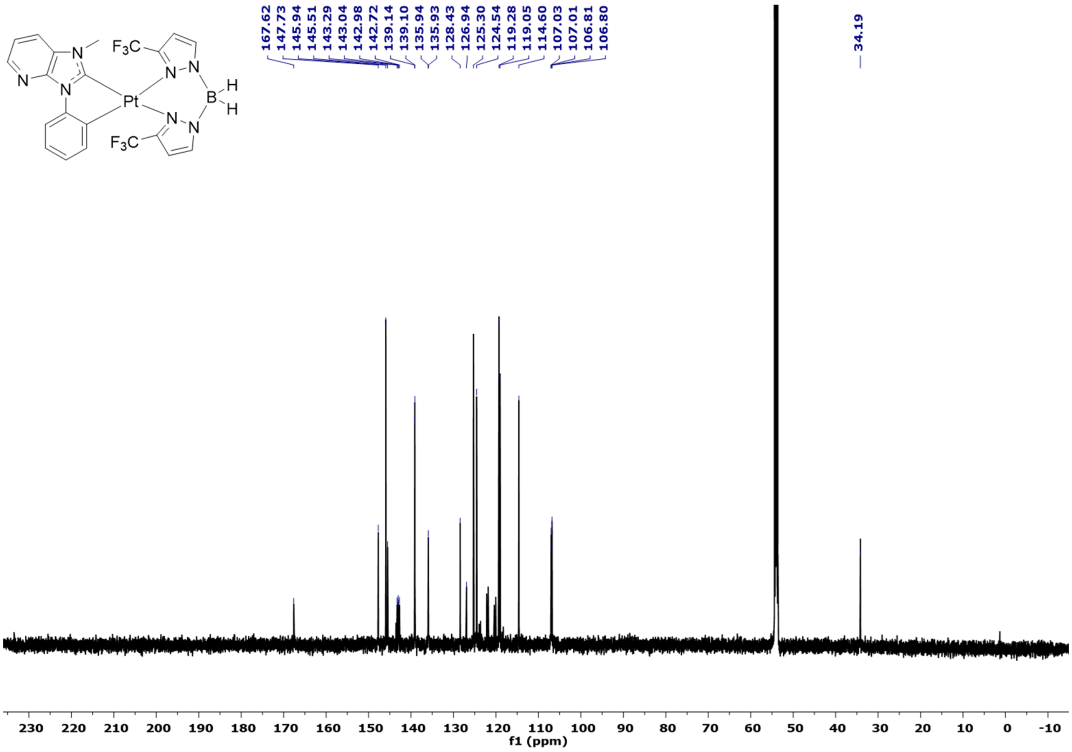

Figure S46: ^{13}C NMR spectrum (151 MHz) of **5a** in DCM-d_2 at room temperature.

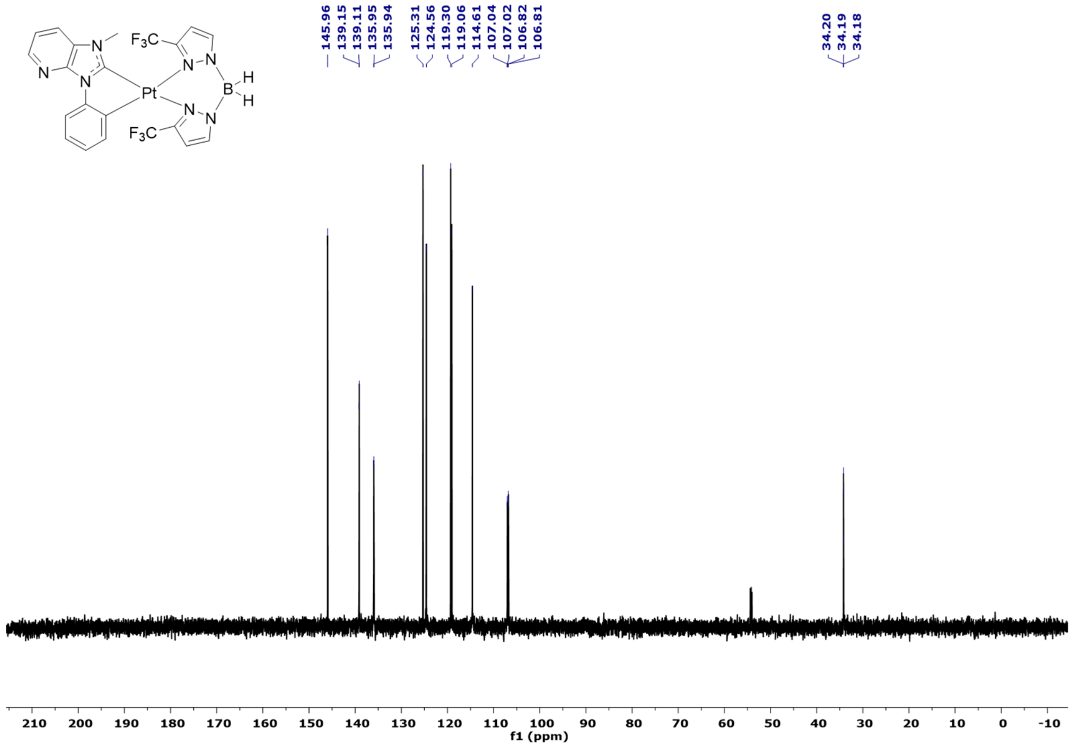

Figure S47: ^{13}C NMR (DEPT-135) spectrum (151 MHz) of **5a** in DCM-d$_2$ at room temperature.

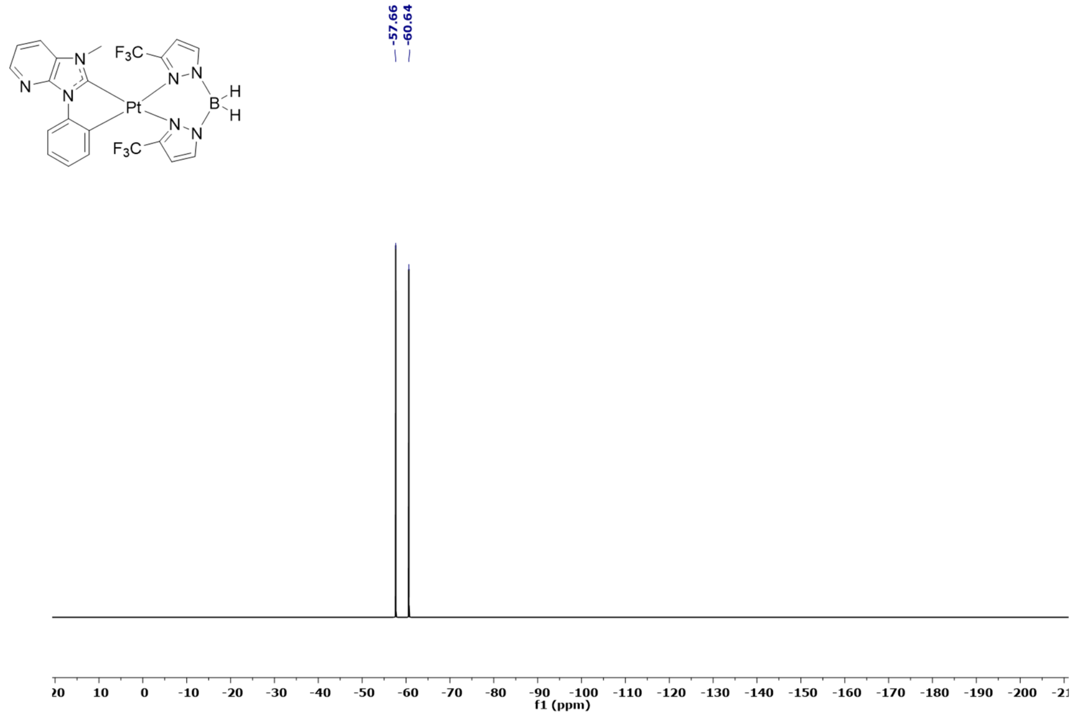

Figure S48: ^{19}F NMR spectrum (282 MHz) of **5a** in DCM-d$_2$ at room temperature.

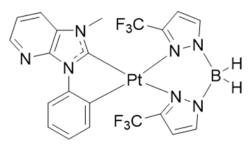

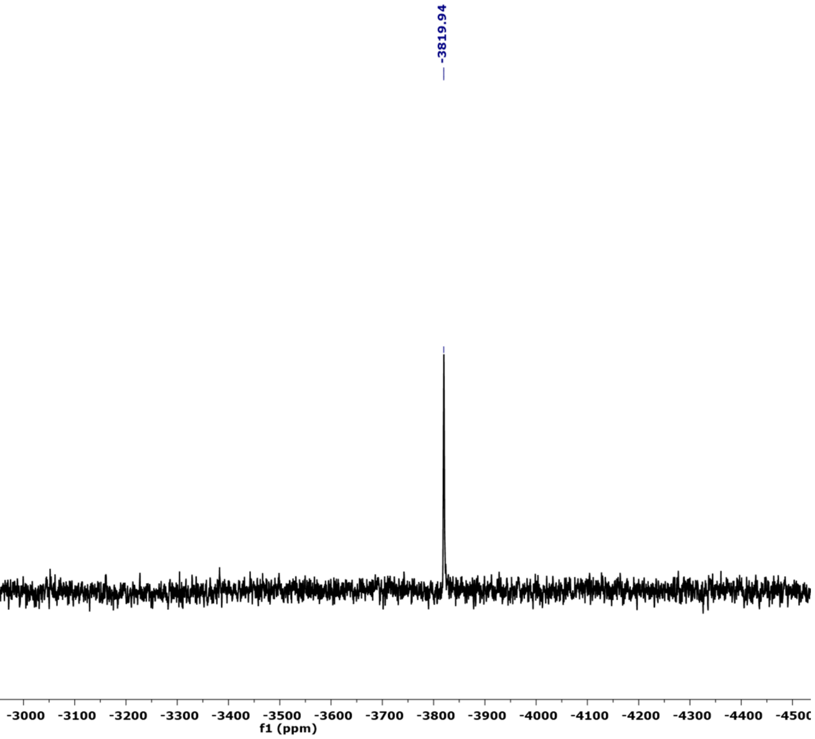

Figure S49: ^{195}Pt NMR spectrum (129 MHz) of **5a** in DCM-d_2 at room temperature.

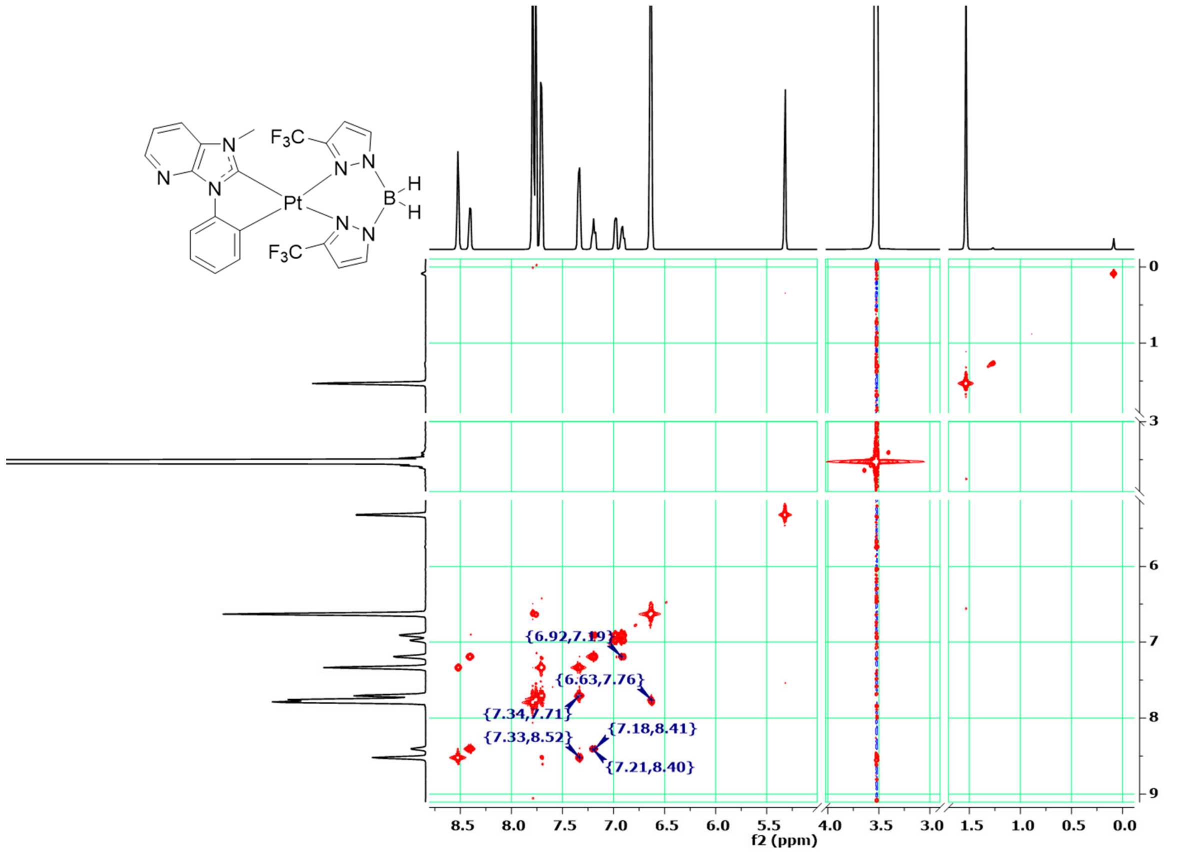

Figure S50: COSY of **5a** in DCM-d_2 at room temperature.

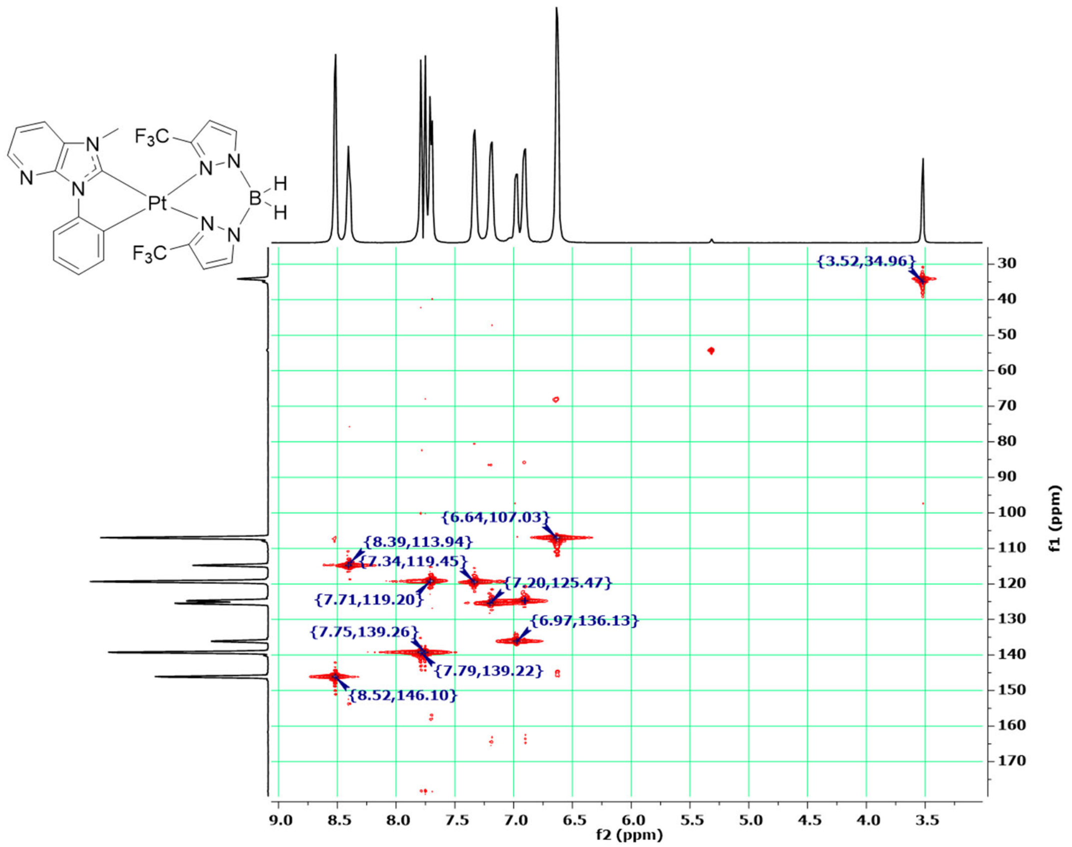

Figure S51: HSQC of **5a** in DCM-d_2 at room temperature.

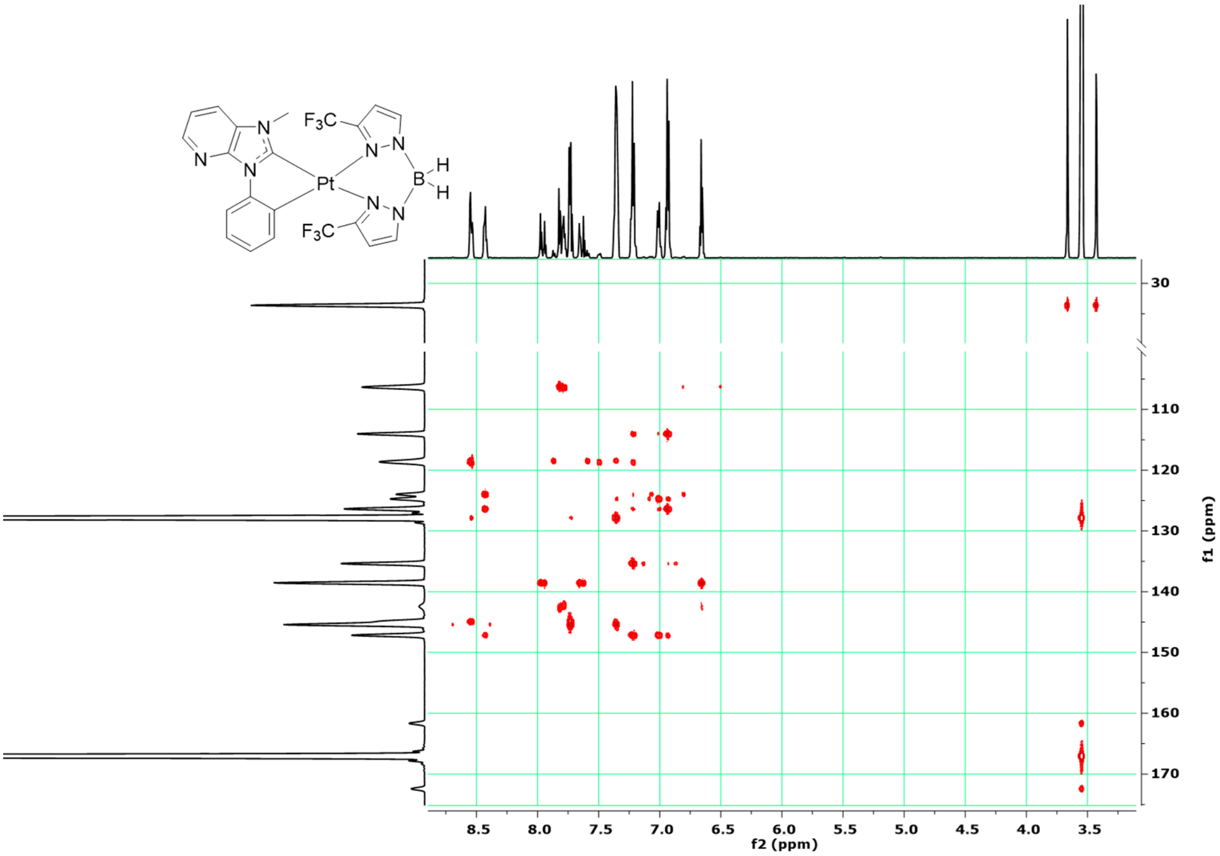

Figure S52: HMBC of **5a** in DCM-d_2 at room temperature.

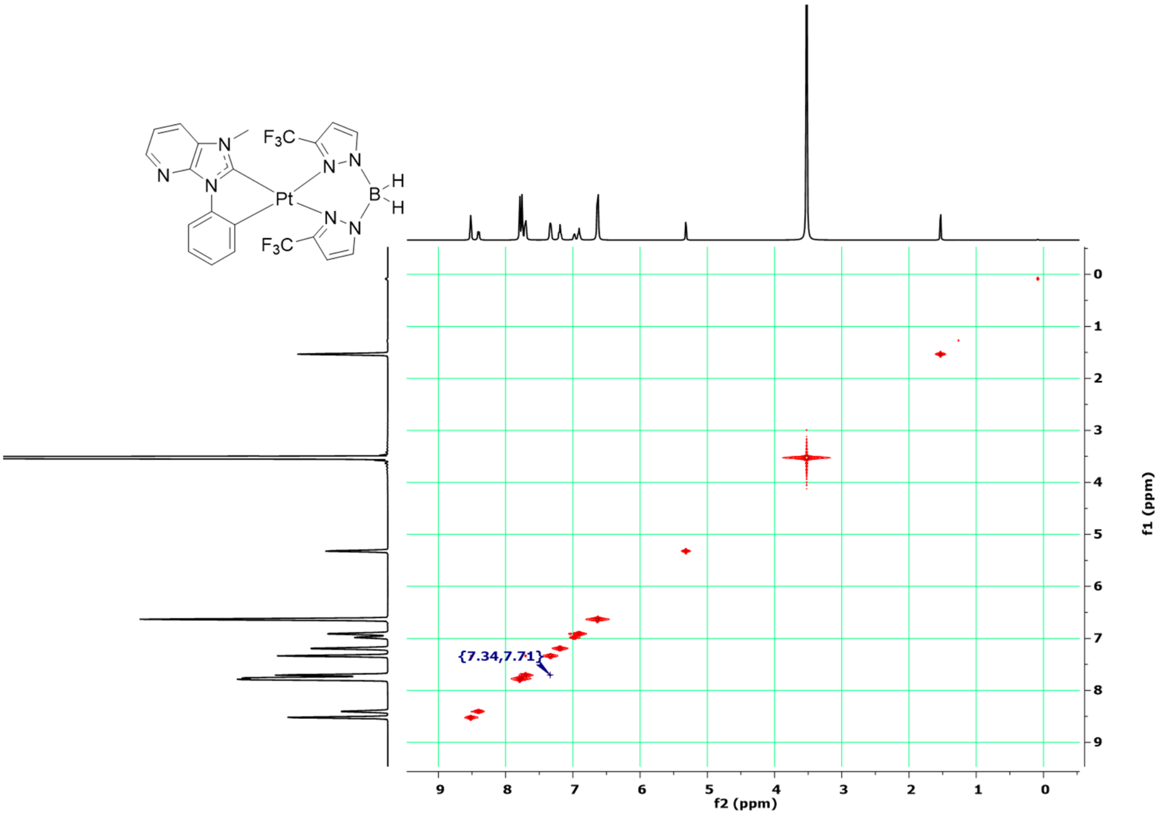

Figure S53: NOESY of **5a** in DCM-d$_2$ at room temperature.

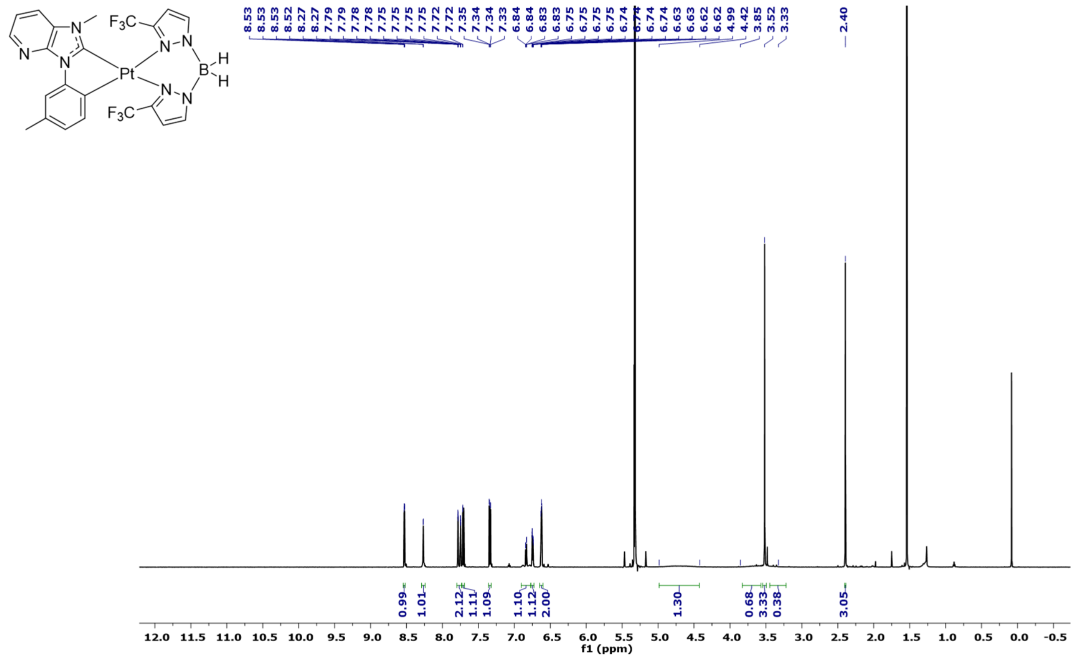

Figure S54: ^{1}H NMR (600 MHz) of **5b** in DCM-d_2 at room temperature.

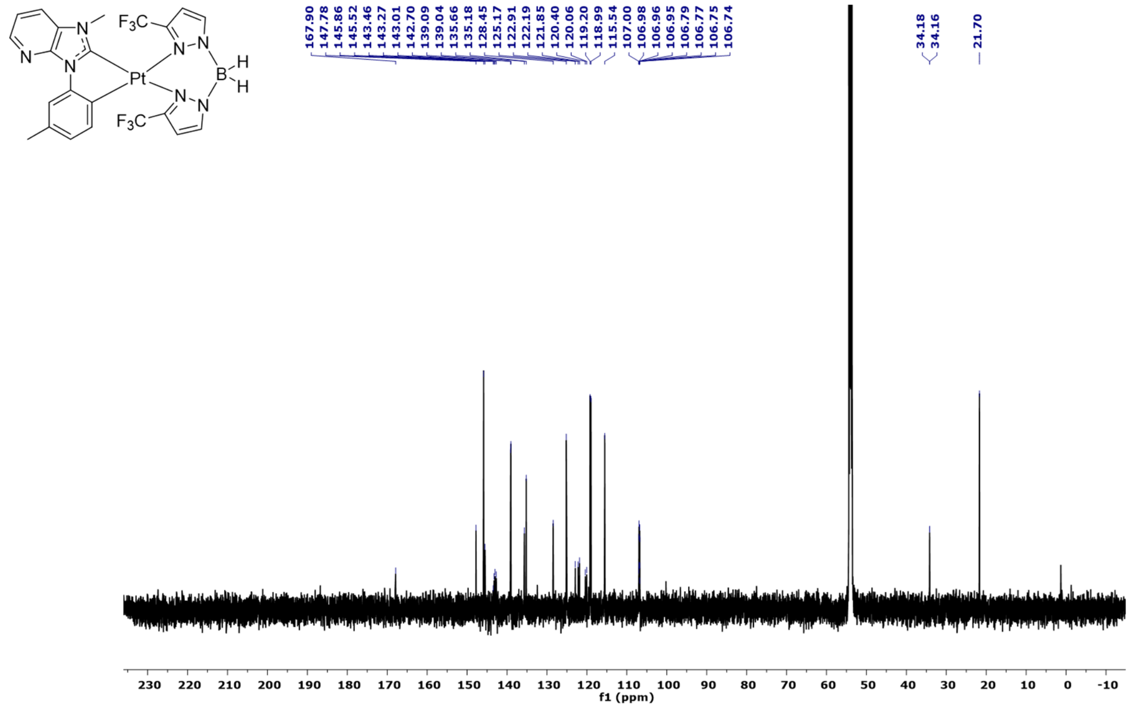

Figure S55: ^{13}C NMR (75 MHz) of **5b** in DCM-d_2 at room temperature.

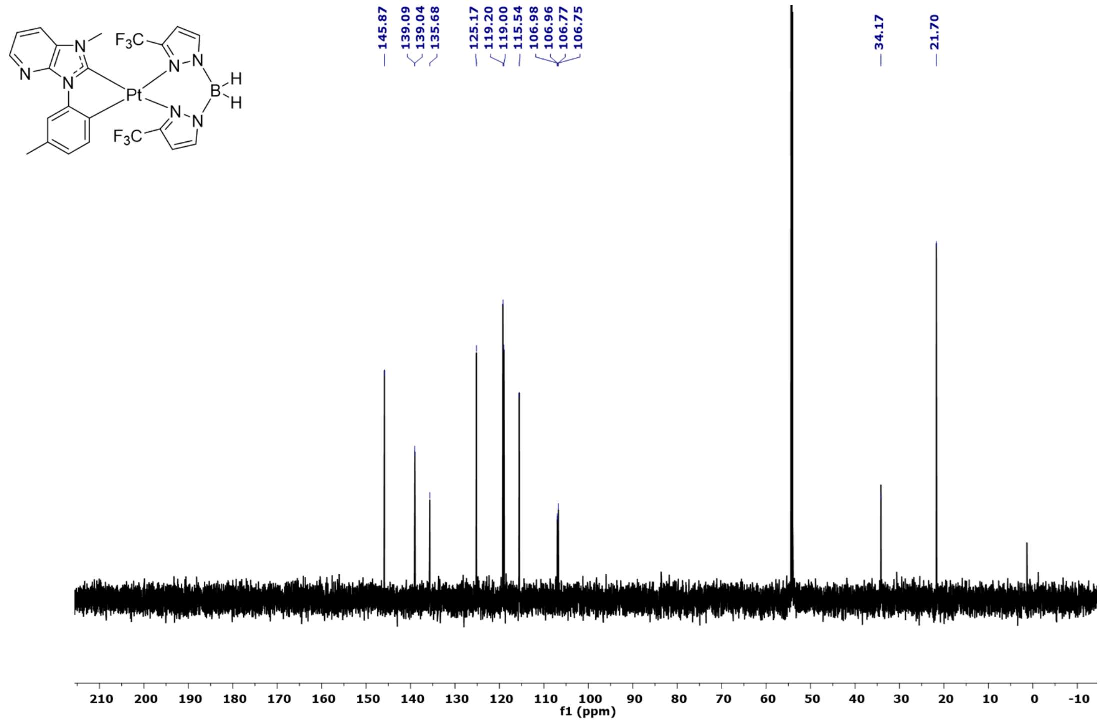

Figure S56: ^{13}C NMR (DEPT-135) (75 MHz) of **5b** in DCM-d$_2$ at room temperature.

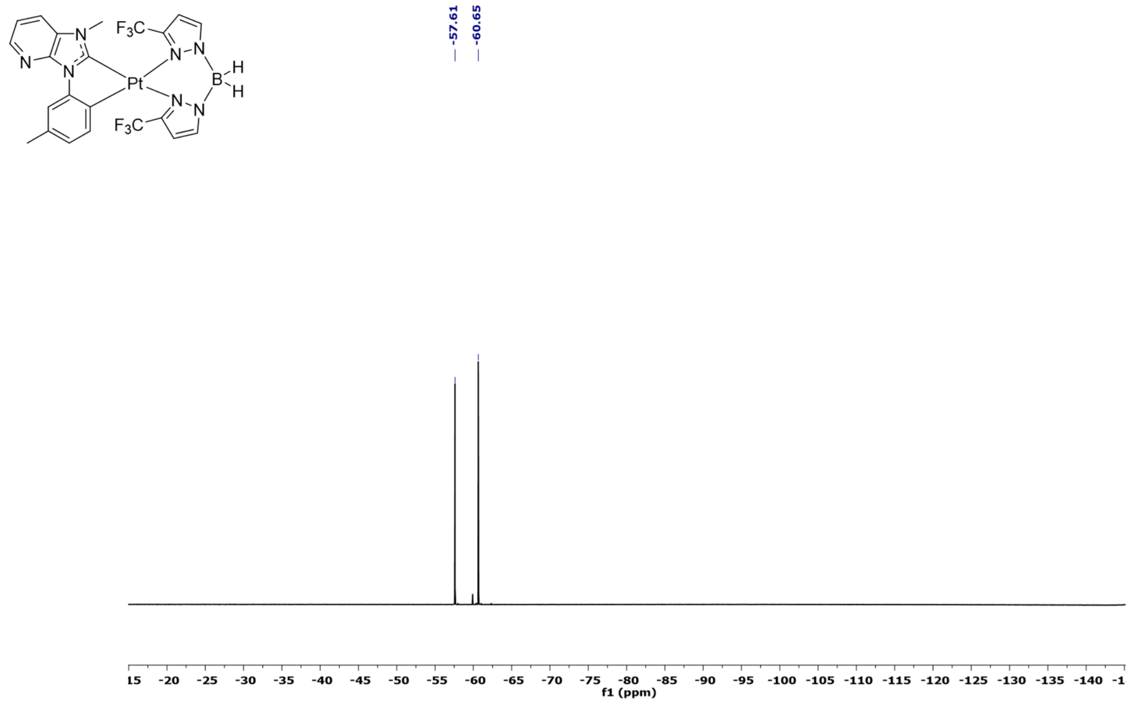

Figure S57: ^{19}F NMR (565 MHz) of **5b** in DCM-d$_2$ at room temperature.

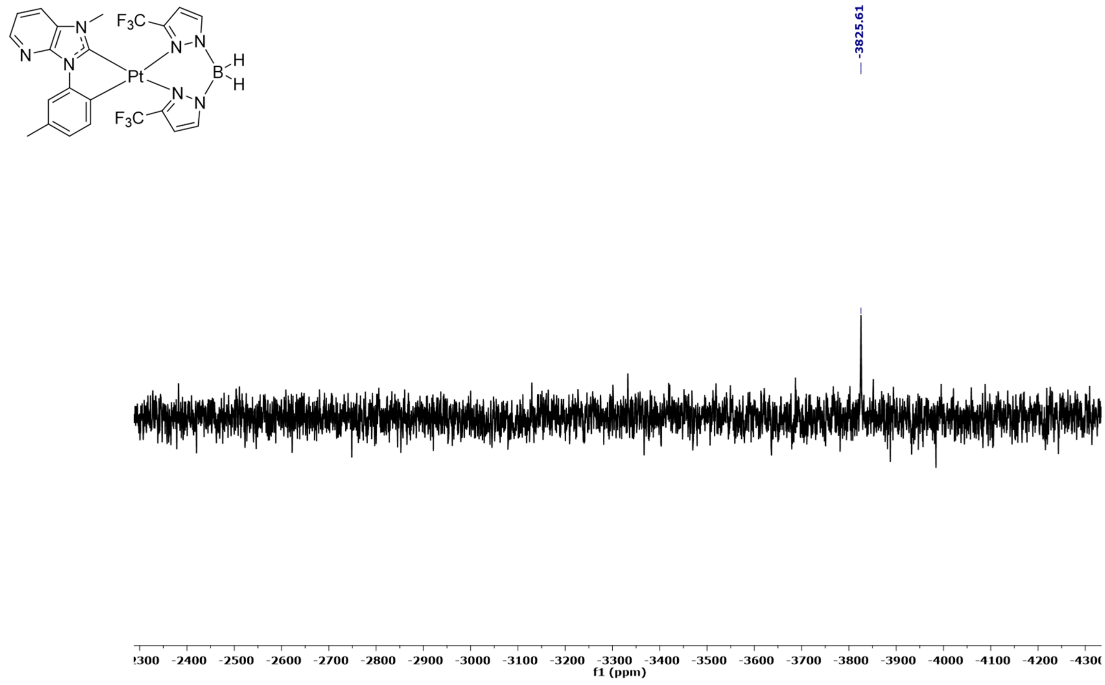

Figure S58: ^{195}Pt NMR (129 MHz) of **5b** in DCM-d_2 at room temperature.

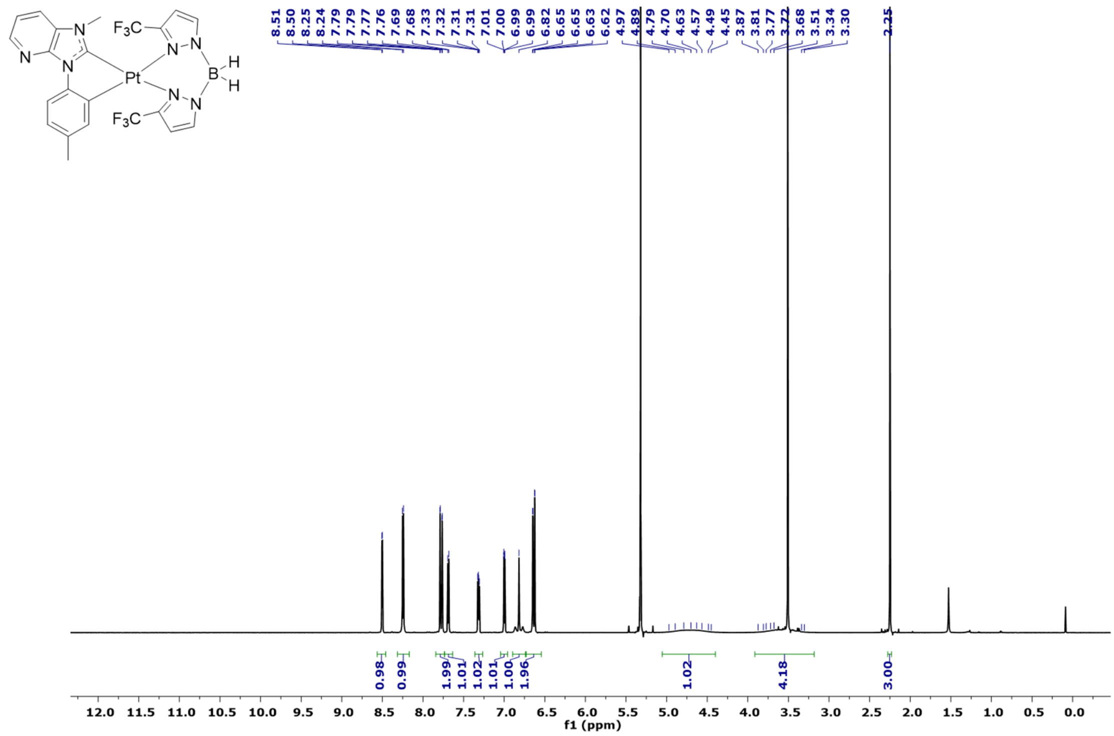

Figure S59: ^{1}H NMR (600 MHz) of **5c** in DCM-d_2 at room temperature.

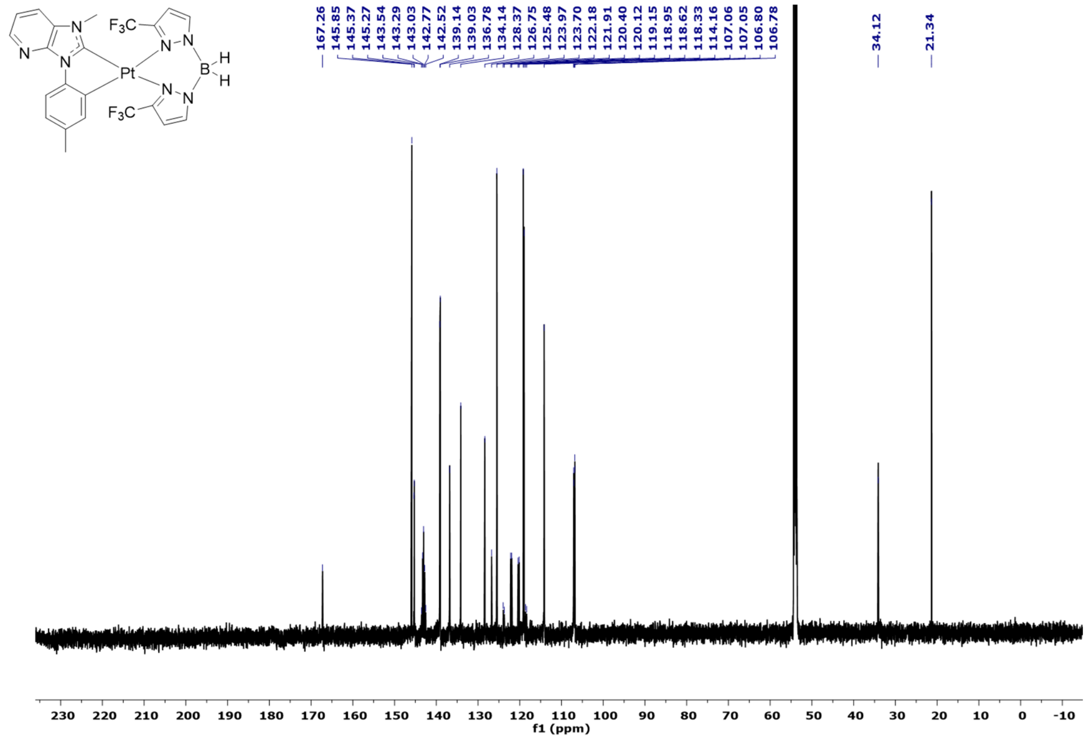

Figure S60: ^{13}C NMR (151 MHz) of **5c** in DCM-d_2 at room temperature.

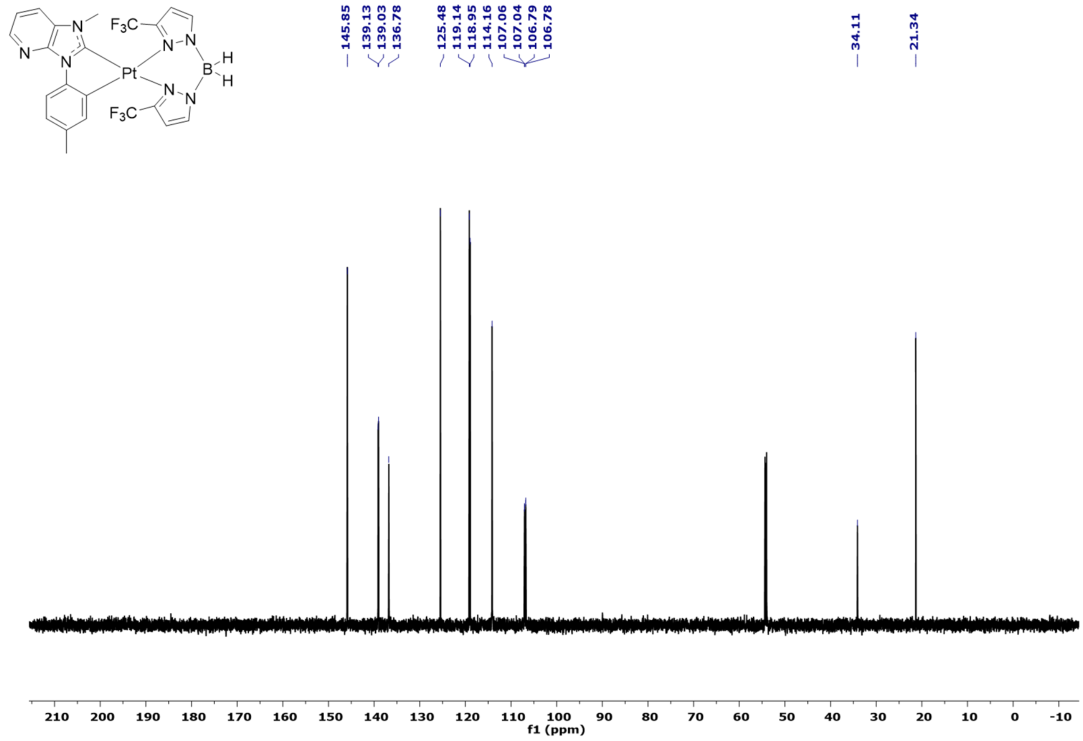

Figure S61: ^{13}C NMR (DEPT-135) (151 MHz) of **5c** in DCM-d_2 at room temperature.

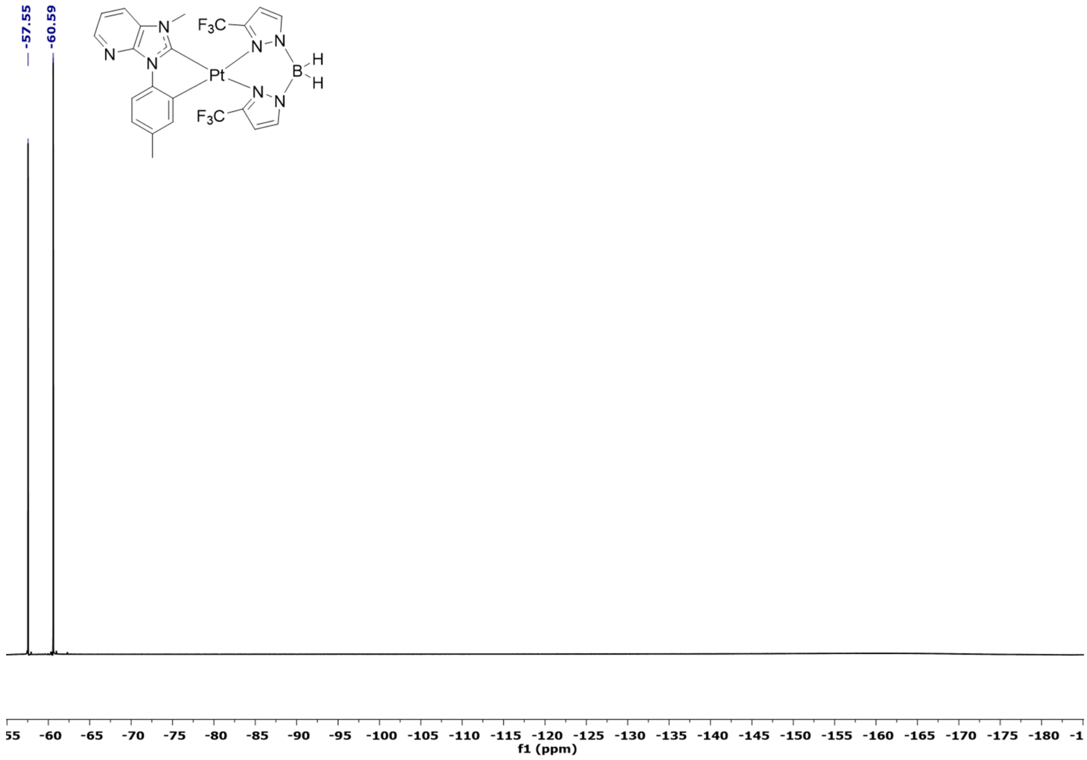

Figure S62: ^{19}F NMR (565 MHz) of **5c** in DCM-d_2 at room temperature.

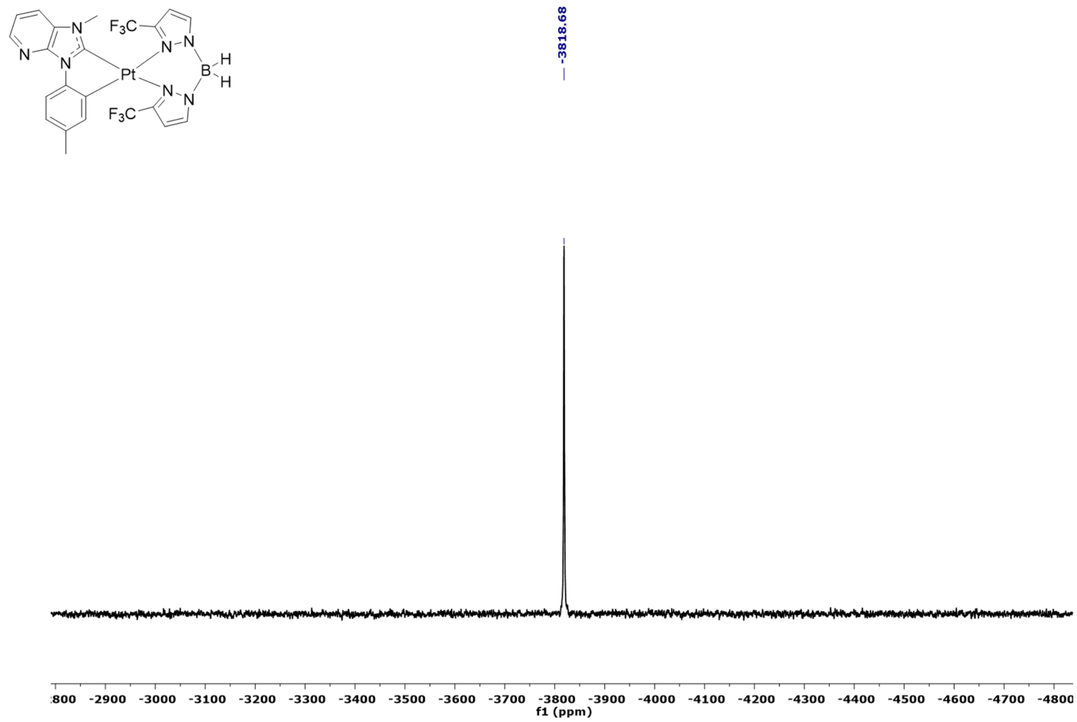

Figure S63: ^{195}Pt NMR (129 MHz) of **5c** in DCM-d_2 at room temperature.

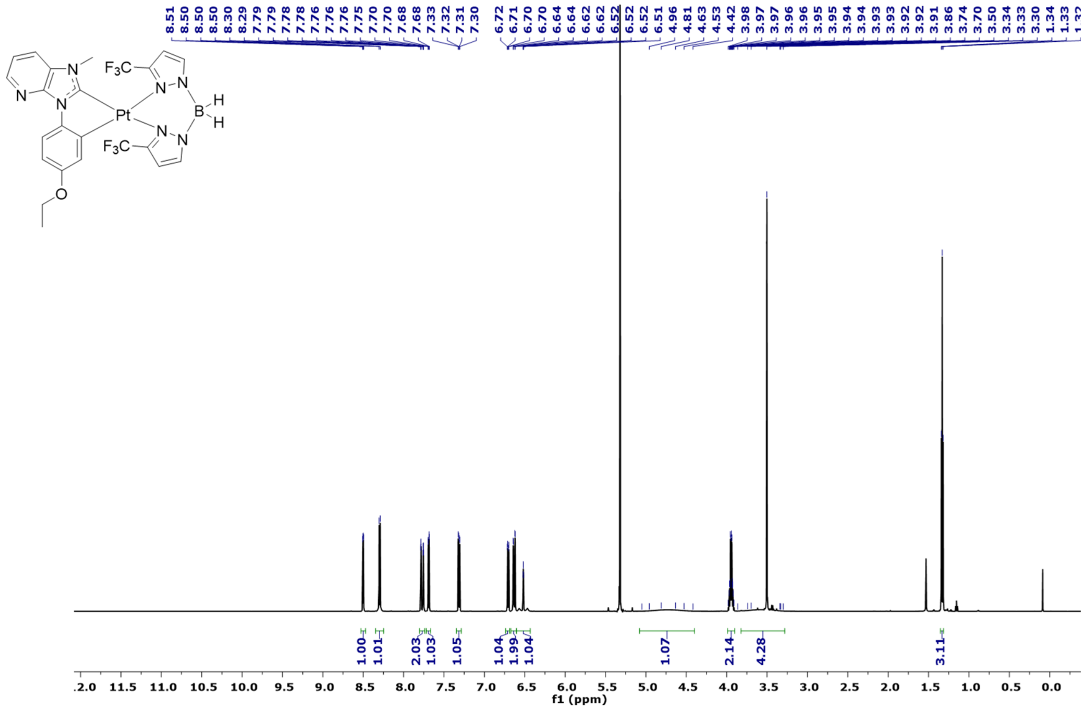

Figure S64: ^{1}H NMR (600 MHz) of **5d** in DCM-d_2 at room temperature.

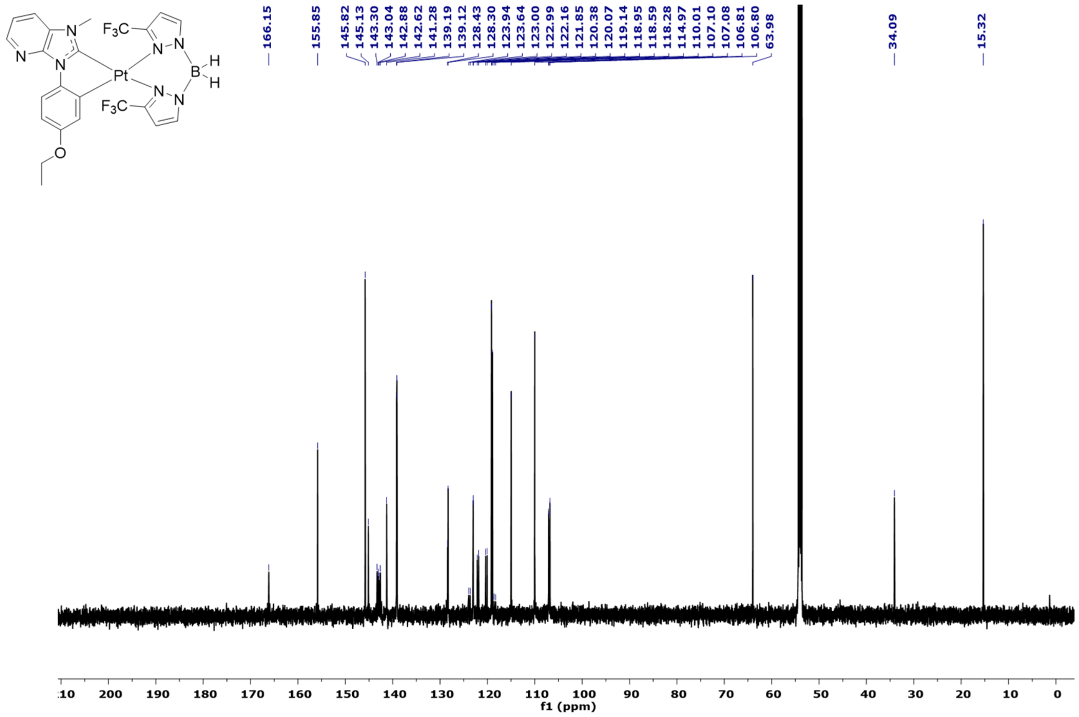

Figure S65: ^{13}C NMR (151 MHz) of **5d** in DCM-d_2 at room temperature.

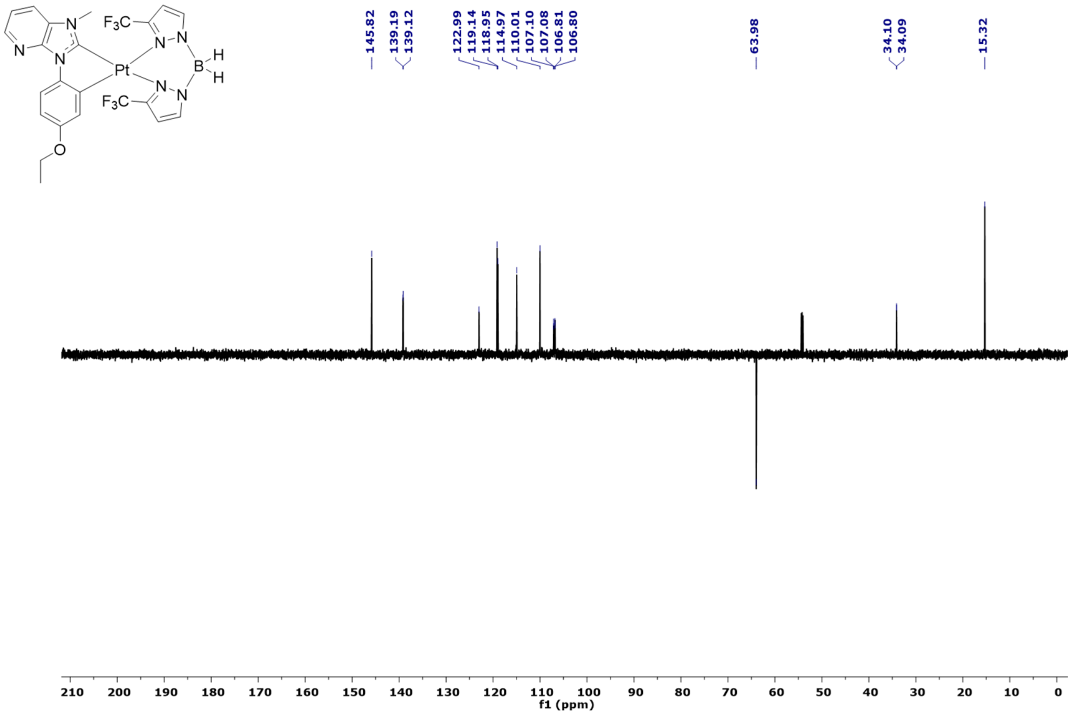

Figure S66: ^{13}C NMR (DEPT-135) (151 MHz) of **5d** in DCM-d_2 at room temperature.

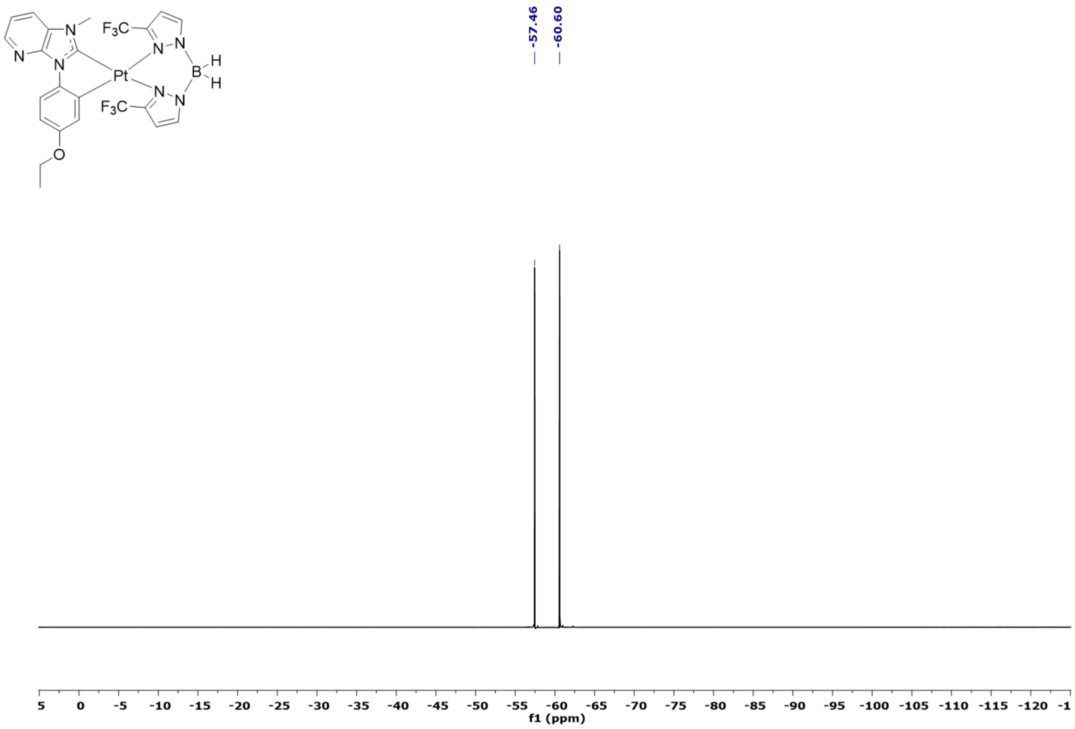

Figure S67: ^{19}F NMR (565 MHz) of **5d** in DCM-d_2 at room temperature.

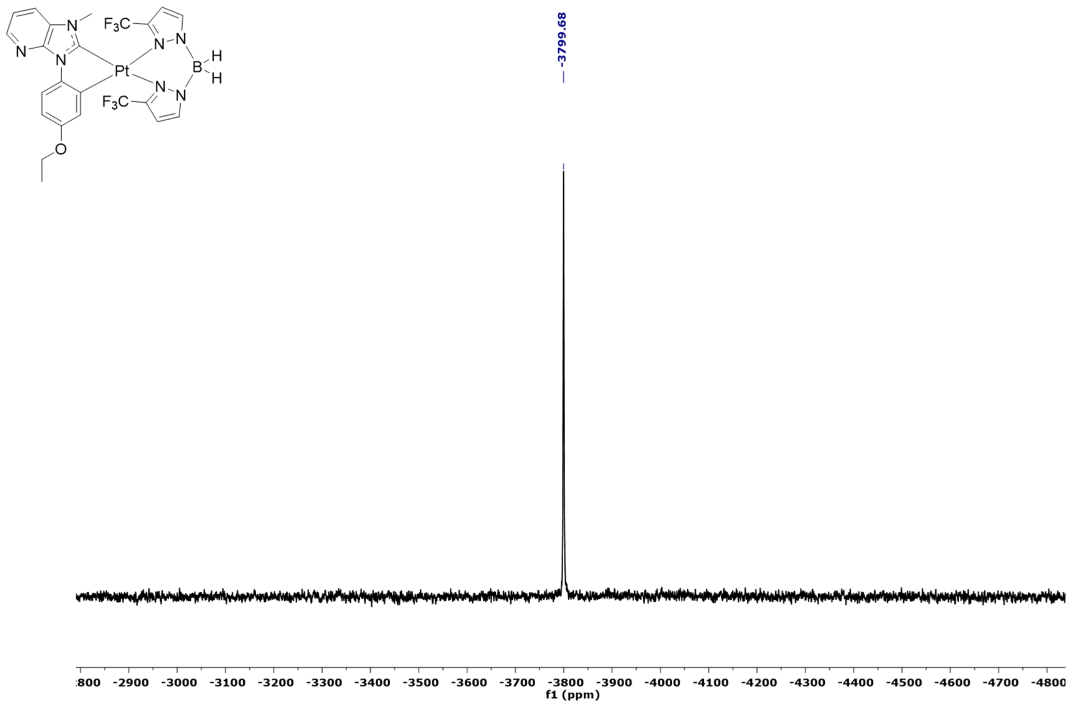

Figure S68: ^{195}Pt NMR (129 MHz) of **5d** in DCM-d$_2$ at room temperature.

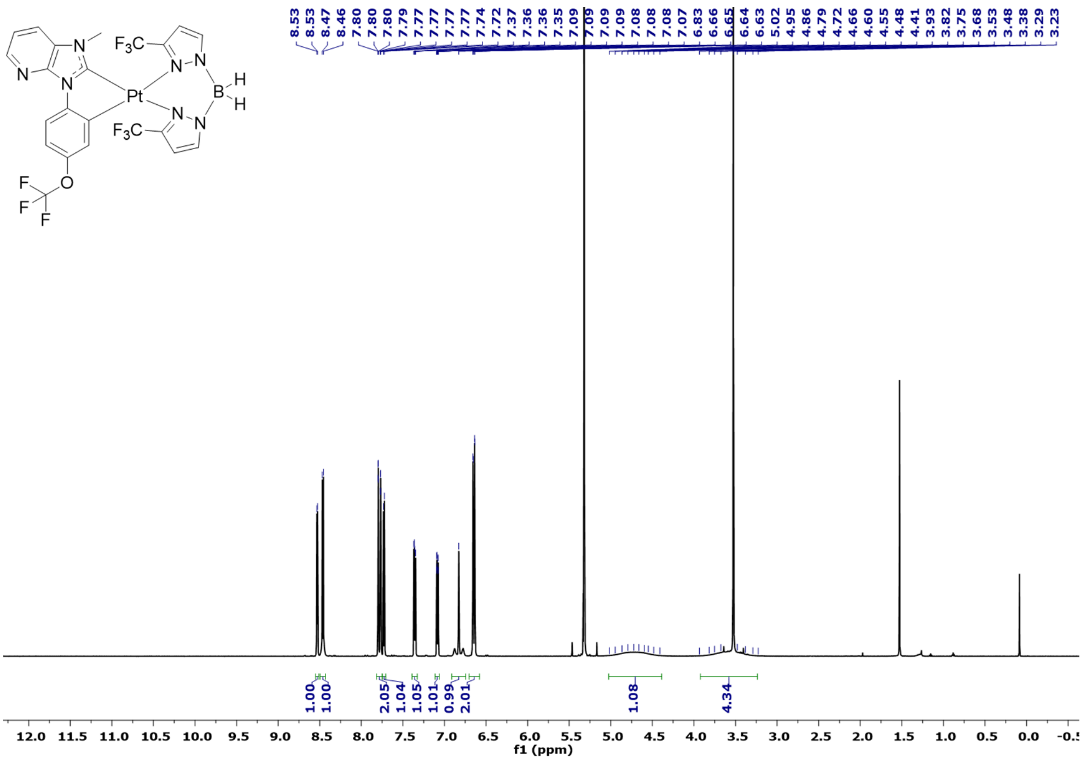

Figure S69: ^{1}H NMR (600 MHz) of **5e** in DCM-d$_2$ at room temperature.

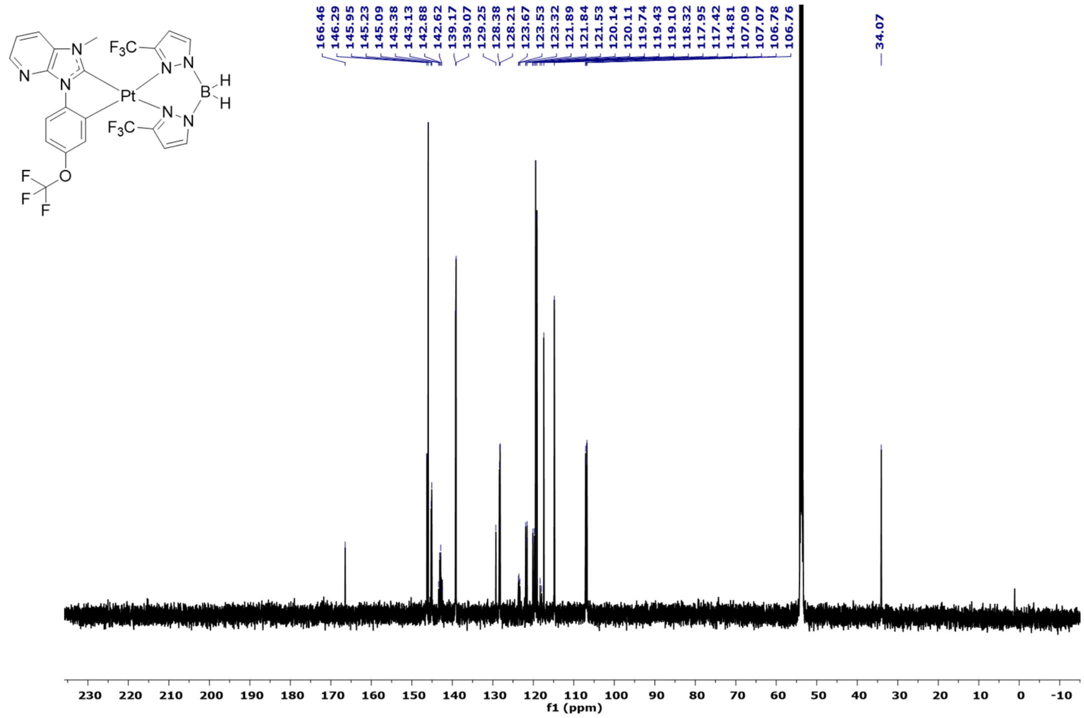

Figure S70: ^{13}C NMR (151 MHz) of **5e** in DCM-d$_2$ at room temperature.

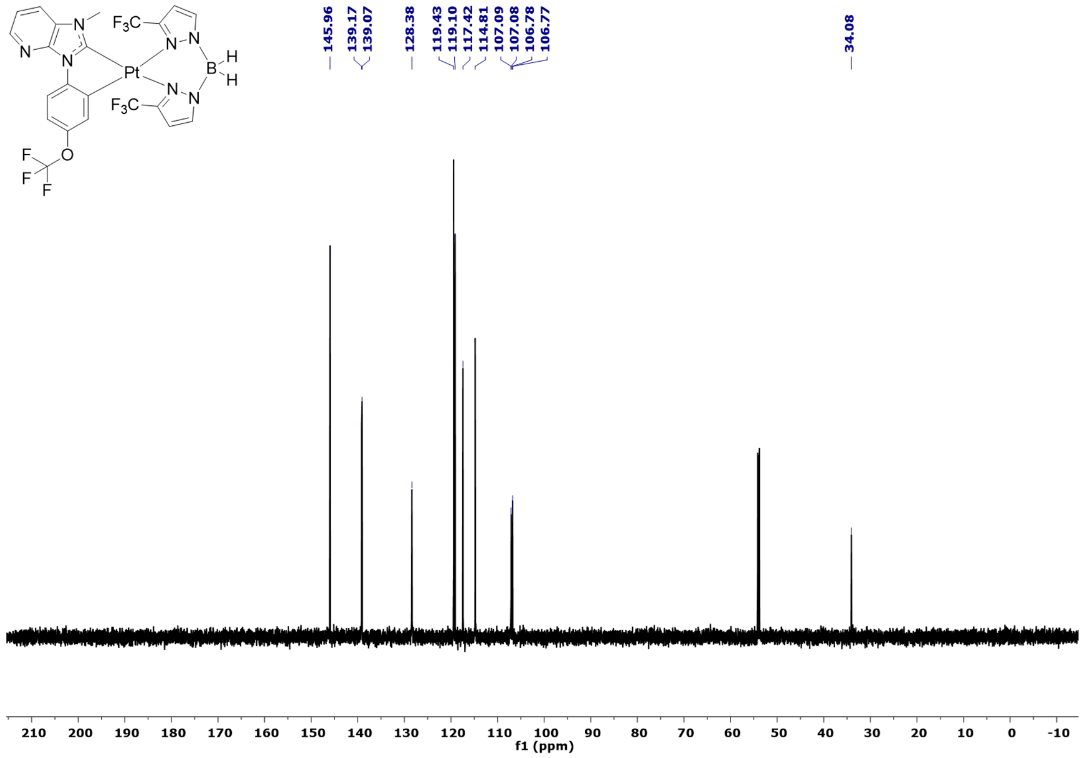

Figure S71: ^{13}C NMR (DEPT-135) (151 MHz) of **5e** in DCM-d$_2$ at room temperature.

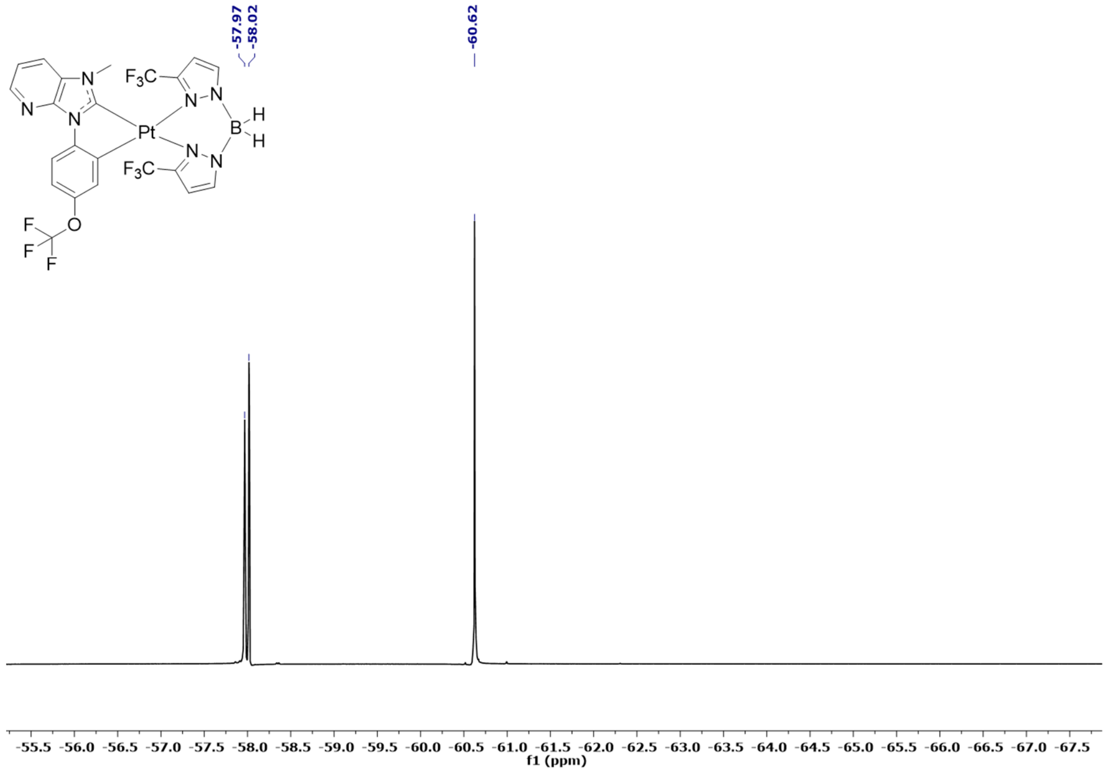

Figure S72: ^{19}F NMR (565 MHz) of **5e** in DCM-d_2 at room temperature.

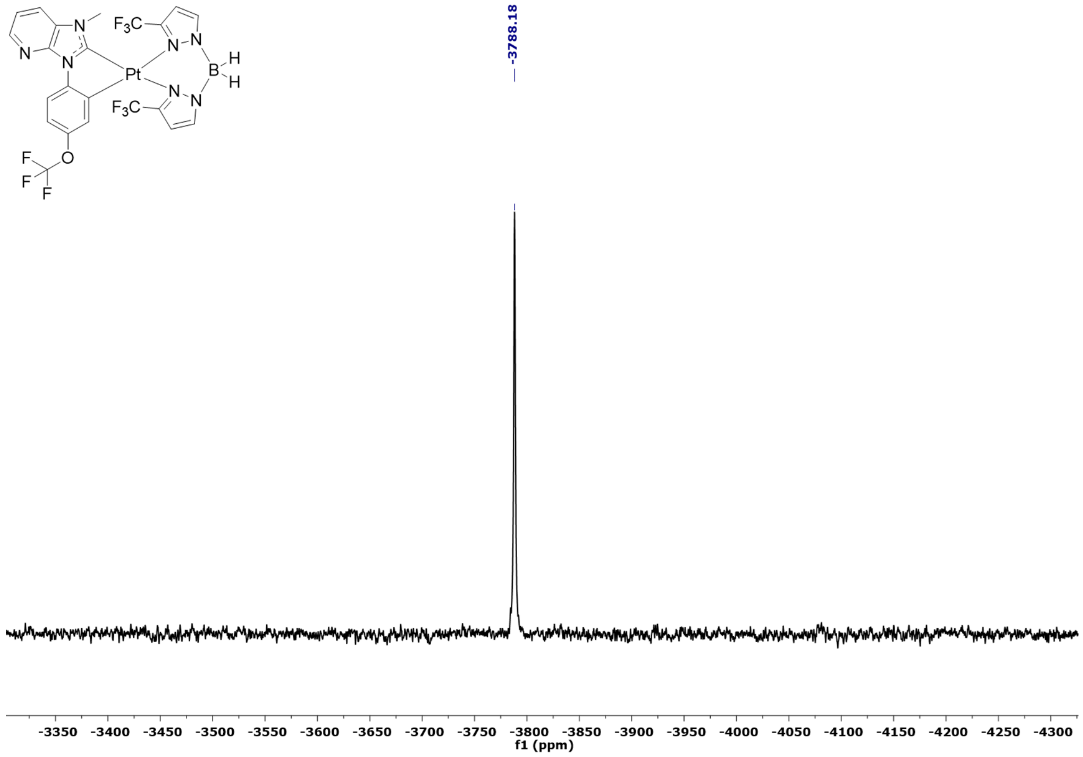

Figure S73: ^{195}Pt NMR (129 MHz) of **5e** in DCM-d_2 at room temperature.

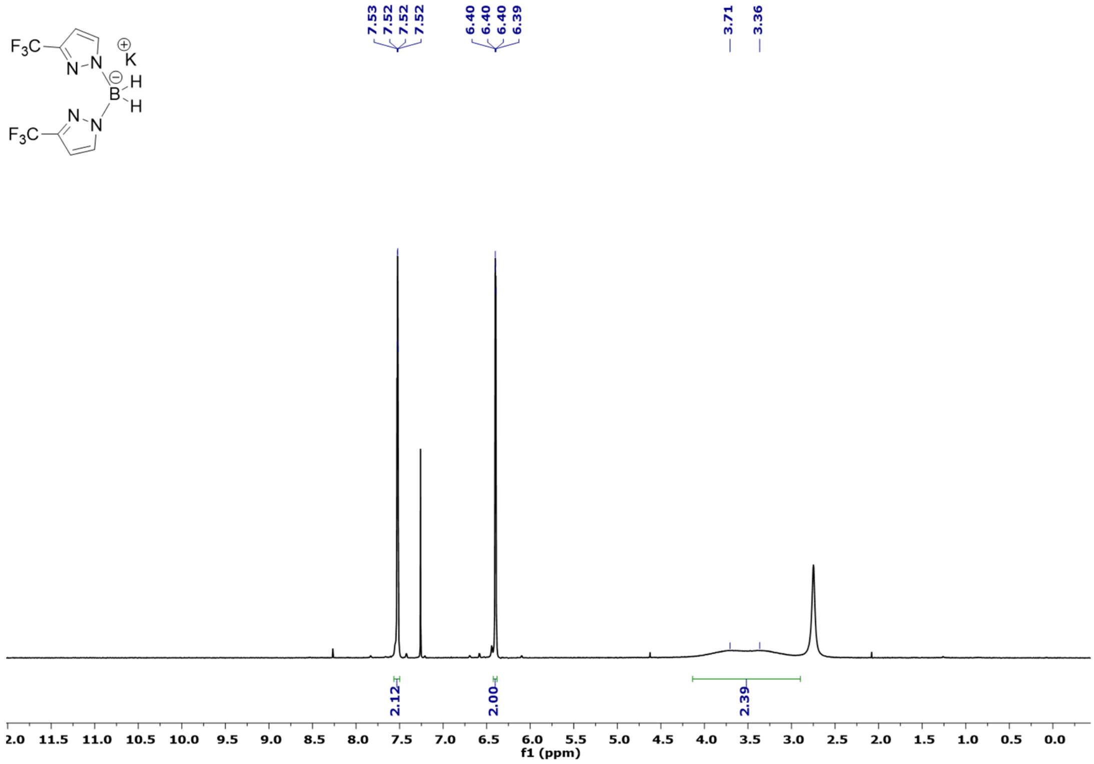

Figure S74: ^{1}H NMR spectrum (300 MHz) of **6** in $CDCl_3$ at room temperature.

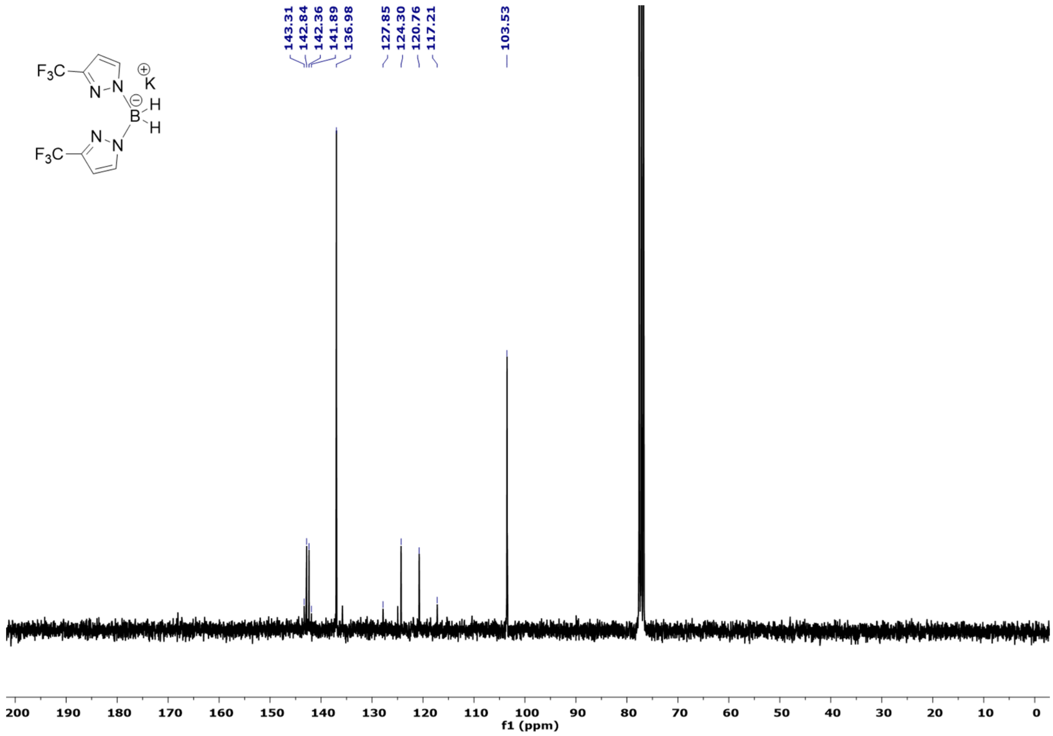

Figure S75: ^{13}C NMR spectrum (75 MHz) of **6** in $CDCl_3$ at room temperature.

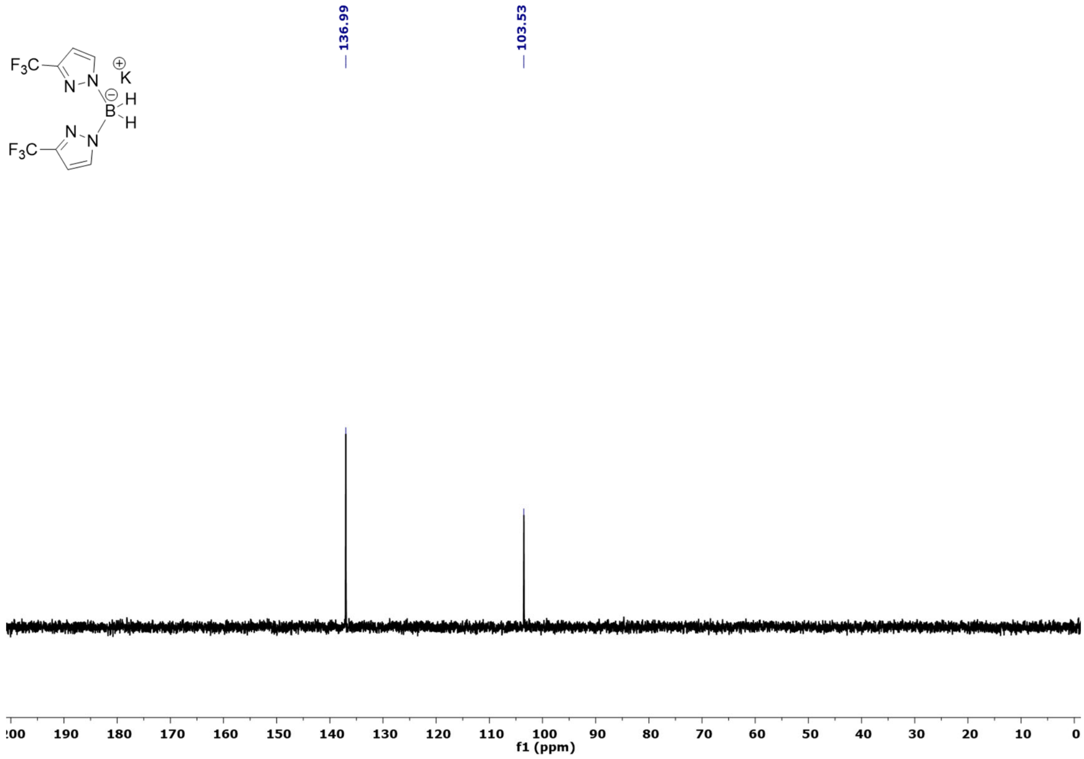

Figure S76: ^{13}C NMR (DEPT-135) spectrum (75 MHz) of **6** in $CDCl_3$ at room temperature.

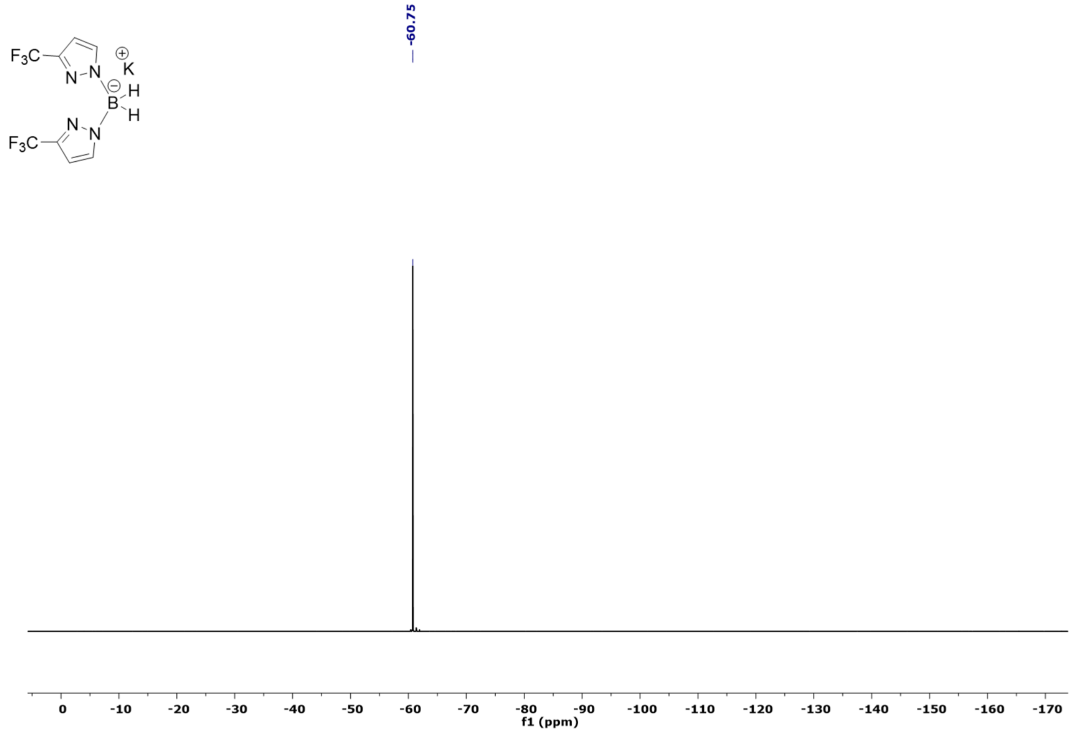

Figure S77: ^{19}F NMR spectrum (282 MHz) of **6** in $CDCl_3$ at room temperature.

Solid State Structures

Complex **5b**

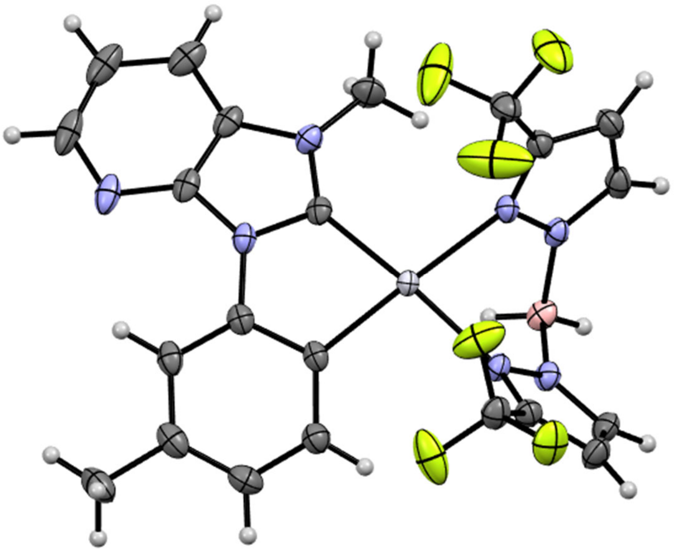

Figure S78: Crystal structure of complex **5b** (C2/c) with thermal ellipsoids at 50% probability.

Identification code	CCDC 2431633	
Empirical formula	C22 H18 B F6 N7 Pt	
Formula weight	700.33	
Temperature	150(2) K	
Wavelength	0.71073 Å	
Crystal system	Monoclinic	
Space group	C2/c	
Unit cell dimensions	a = 35.5712(19) Å	α = 90°.
	b = 9.3856(5) Å	β = 112.031(2)°.
	c = 14.7692(8) Å	γ = 90°.
Volume	4570.8(4) $Å^3$	
Z	8	
Density (calculated)	2.035 Mg/m^3	
Absorption coefficient	6.216 mm^{-1}	
F(000)	2688	
Crystal size	0.283 x 0.156 x 0.139 mm^3	
Theta range for data collection	3.526 to 27.500°.	

Index ranges	-46<=h<=46, -12<=k<=12, -19<=l<=19
Reflections collected	72871
Independent reflections	5237 [R(int) = 0.0421]
Completeness to theta = 25.242°	99.6 %
Absorption correction	Multi Scan
Max. and min. transmission	0.7457 and 0.5308
Refinement method	Full-matrix least-squares on F^2
Data / restraints / parameters	5237 / 0 / 336
Goodness-of-fit on F^2	1.163
Final R indices [I>2sigma(I)]	R1 = 0.0177, wR2 = 0.0413
R indices (all data)	R1 = 0.0191, wR2 = 0.0423
Extinction coefficient	n/a
Largest diff. peak and hole	0.673 and -1.090 e.Å^{-3}

Complex **5e**

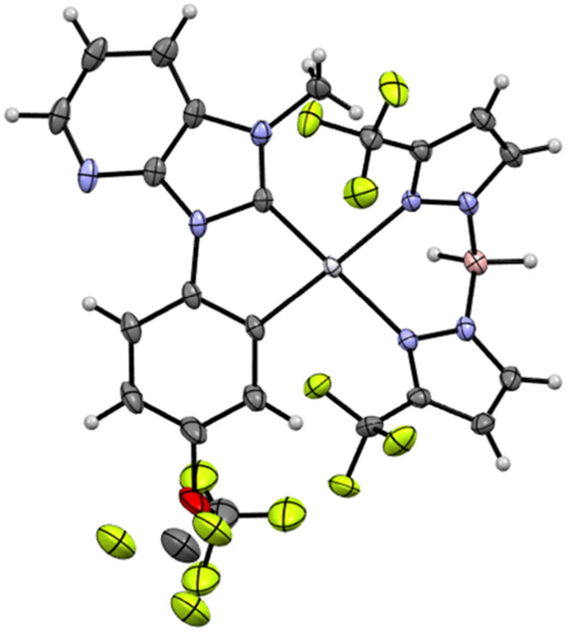

Figure S79: Crystal structure of complex 5e (P-1) with thermal ellipsoids at 50% probability. The disordered OCF_3-group was modeled over two positions with site occupancies refined to 51.6% and 48.4%. Equivalent anisotropic displacement parameters (EADP) were applied to corresponding atoms in both conformations.

Identification code	CCDC 2431632	
Empirical formula	C22 H15 B F9 N7 O Pt	
Formula weight	770.31	
Temperature	100(2) K	
Wavelength	0.71073 Å	
Crystal system	Triclinic	
Space group	P-1	
Unit cell dimensions	a = 7.0836(5) Å	α = 102.744(2)°.
	b = 12.8363(9) Å	β = 98.410(2)°.
	c = 15.6136(10) Å	γ = 99.622(2)°.
Volume	1340.26(16) $Å^3$	
Z	2	
Density (calculated)	1.909 Mg/m^3	
Absorption coefficient	5.328 mm^{-1}	
F(000)	736	

Crystal size	0.396 x 0.303 x 0.182 mm^3
Theta range for data collection	3.090 to 28.375°.
Index ranges	-9<=h<=9, -17<=k<=17, -20<=l<=20
Reflections collected	59509
Independent reflections	6686 [R(int) = 0.0406]
Completeness to theta = 25.242°	99.4 %
Absorption correction	Multi Scan
Max. and min. transmission	0.7457 and 0.5722
Refinement method	Full-matrix least-squares on F^2
Data / restraints / parameters	6686 / 0 / 385
Goodness-of-fit on F^2	1.091
Final R indices [I>2sigma(I)]	R1 = 0.0237, wR2 = 0.0612
R indices (all data)	R1 = 0.0247, wR2 = 0.0621
Extinction coefficient	n/a
Largest diff. peak and hole	1.890 and -1.060 e.$Å^{-3}$

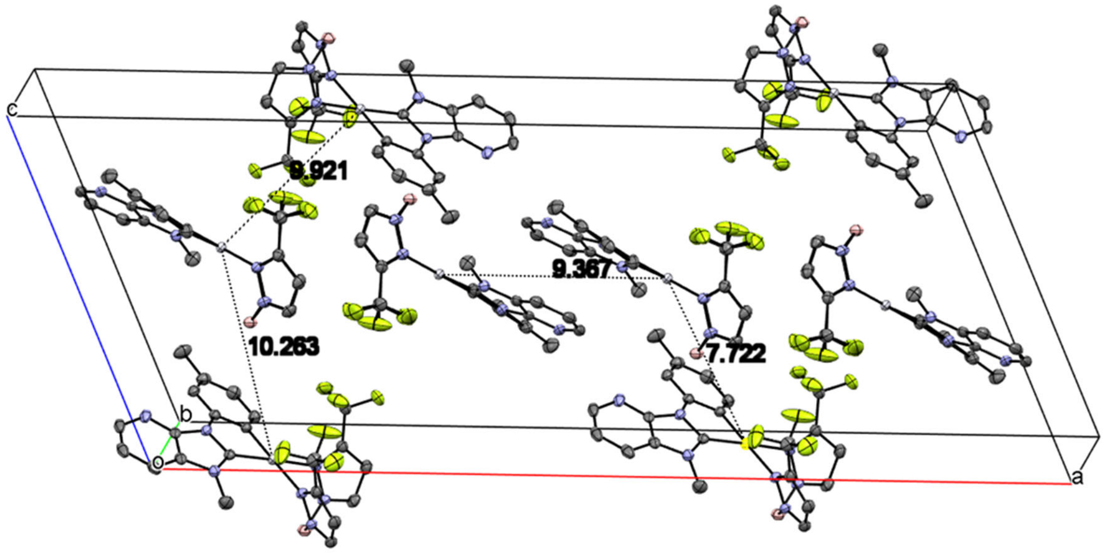

Figure S80: Solid state packing of **5b**. Hydrogen atoms are omitted for clearance.

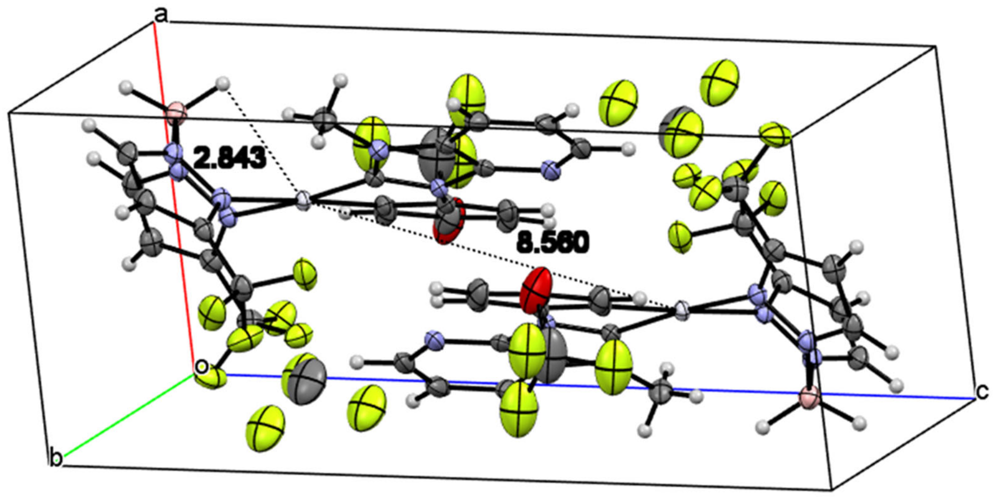

Figure S81: Solid state packing of **5e**. Hydrogen atoms are omitted for clearance.

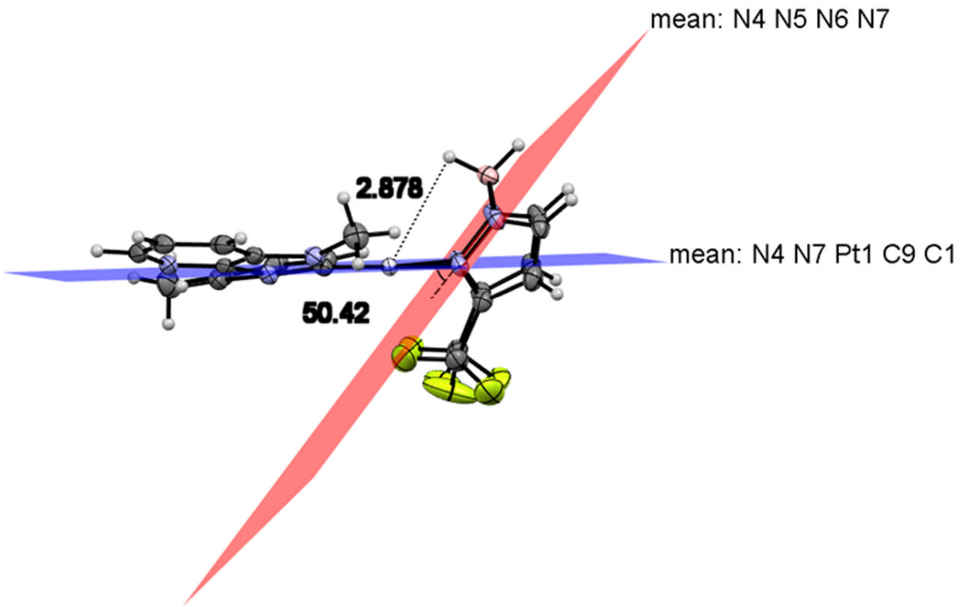

Figure S82: Crystal structures of complex **5b** with intersection planes spanned by the C^C* ligand bound to the metal center and the borate ligand. Hydrogen atoms are omitted for better clarity.

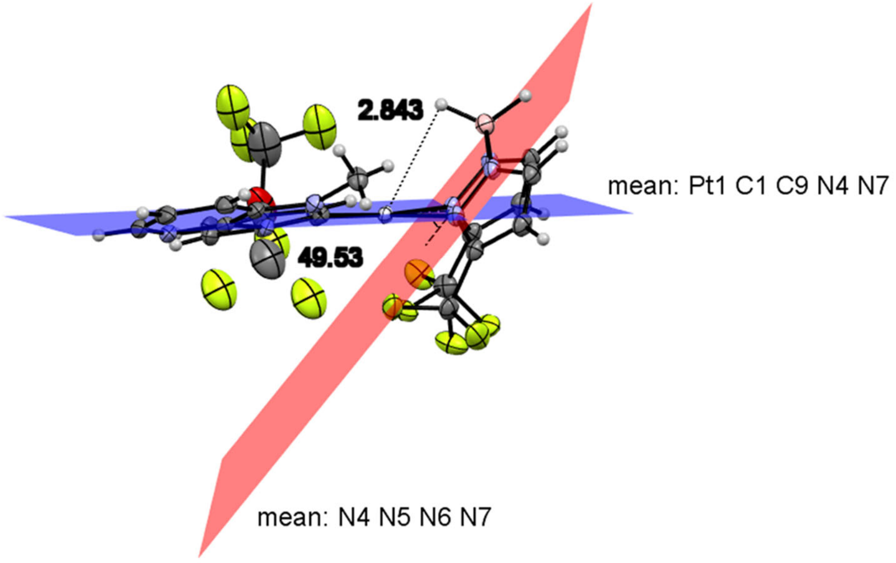

Figure S83: Crystal structures of complex **5e** with intersection planes spanned by the C^C* ligand bound to the metal center and the borate ligand. Hydrogen atoms are omitted for better clarity.

Frontier Molecular Orbitals

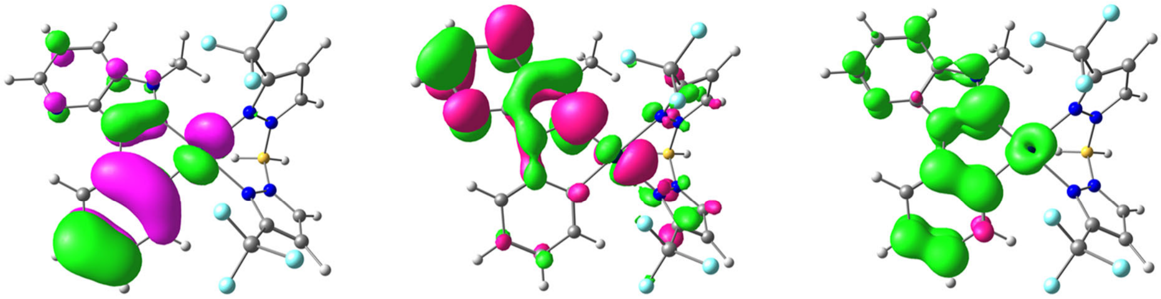

HOMO LUMO Spindensity

Figure S84: Optimized ground-state geometry with HOMO and LUMO distribution plot (isovalue = 0.03) of complex **5a**. Right. Optimized excited-state geometry and spin density distribution plot (isovalue = 0.004) for the lowest-lying triplet state of complex **5a**.

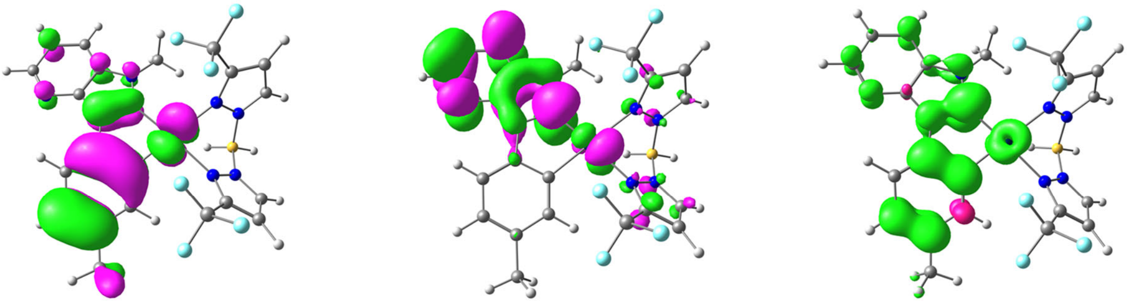

HOMO LUMO Spindensity

Figure S85: Optimized ground-state geometry with HOMO and LUMO distribution plot (isovalue = 0.03) of complex **5c**. Right. Optimized excited-state geometry and spin density distribution plot (isovalue = 0.004) for the lowest-lying triplet state of complex **5c**.

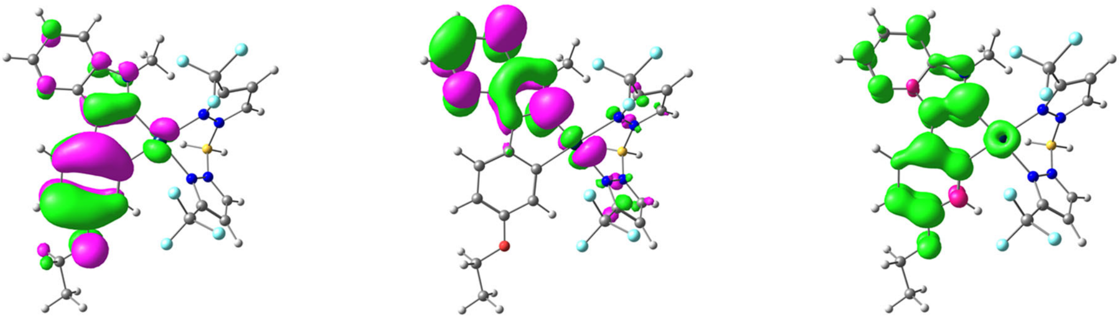

HOMO LUMO Spindensity

Figure S86: Optimized ground-state geometry with HOMO and LUMO distribution plot (isovalue = 0.03) of complex **5d**. Right. Optimized excited-state geometry and spin density distribution plot (isovalue = 0.004) for the lowest-lying triplet state of complex **5d**.

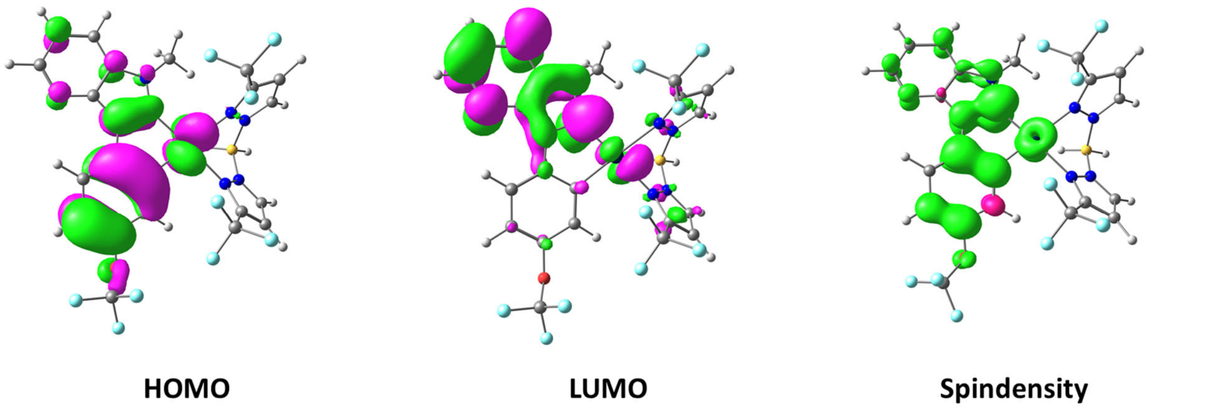

Figure S87: Optimized ground-state geometry with HOMO and LUMO distribution plot (isovalue = 0.03) of complex **5e**. Right. Optimized excited-state geometry and spin density distribution plot (isovalue = 0.004) for the lowest-lying triplet state of complex **5e**.

NTO-analysis of complex 5d

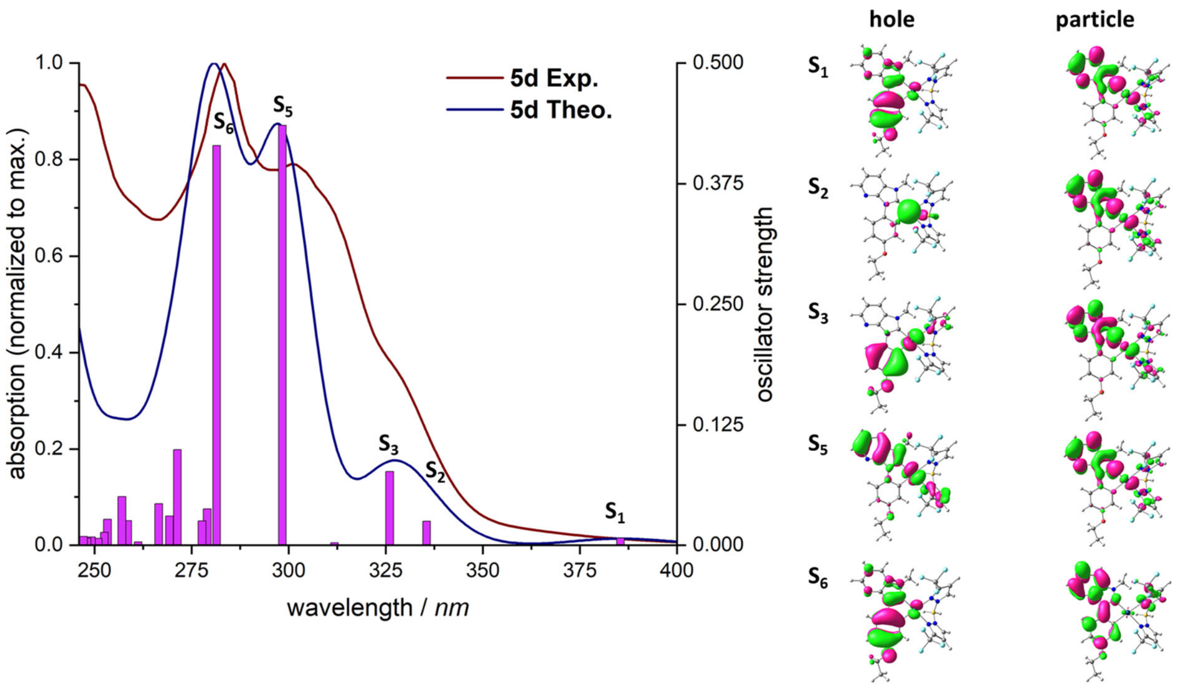

Figure S88: NTO-analysis of complex **5d**.
